# 177 Saponins, Including 11 New Compounds in Wild Ginseng Tentatively Identified via HPLC-IT-TOF-MS^n^, and Differences among Wild Ginseng, Ginseng under Forest, and Cultivated Ginseng

**DOI:** 10.3390/molecules26113371

**Published:** 2021-06-02

**Authors:** Chao-Qun Wang, Li-Wei Yi, Lin Zhao, Yu-Zhen Zhou, Fang Guo, Yu-Shu Huo, Da-Qing Zhao, Feng Xu, Xuan Wang, Shao-Qing Cai

**Affiliations:** 1 Department of Chemical Biology, School of Pharmaceutical Sciences, Peking University Health Science Center, Beijing 100191, China; wangchaoqun8676@live.cn (C.-Q.W.); ylwgloria@126.com (L.-W.Y.); 15201304075@163.com (L.Z.); zhouyuzhen@bjmu.edu.cn (Y.-Z.Z.); gf_0820@163.com (F.G.); 2 University of Texas Health Science Center at San Antonio, San Antonio, TX 78229, USA; yushuh@yahoo.com; 3 School of Pharmaceutical Sciences, Changchun University of Chinese Medicine, Changchun 130117, China; zhaodaqing1963@163.com; 4 State Key Laboratory of Natural and Biomimetic Drugs, School of Pharmaceutical Sciences, Peking University Health Science Center, Beijing 100191, China

**Keywords:** wild ginseng, ginseng under forest, cultivated ginseng, chromatographic fingerprint, saponins contents, HPLC-IT-TOF-MS^n^

## Abstract

Wild ginseng (W-GS), ginseng under forest (F-GS, planted in mountain forest and growing in natural environment), and cultivated ginseng (C-GS) were compared via HPLC-DAD and HPLC-IT-TOF-MS^n^. A total of 199 saponins, including 16 potential new compounds, were tentatively identified from 100 mg W-GS (177 saponins in W-GS with 11 new compounds), F-GS (56 saponins with 1 new compound), and C-GS (60 saponins with 6 new compounds). There were 21 saponins detected from all the W-GS, F-GS, and C-GS. Fifty saponins were only detected from W-GS, including 23 saponins found in ginseng for the first time. Contents of ginsenosides Re (12.36–13.91 mg/g), Rh_1_ (7.46–7.65 mg/g), Rd (12.94–12.98 mg/g), and the total contents (50.52–55.51 mg/g) of Rg_1_, Re, Rf, Rb_1_, Rg_2_, Rh_1_, and Rd in W-GS were remarkably higher than those in F-GS (Re 1.22–3.50 mg/g, Rh_1_ 0.15–1.49 mg/g, Rd 0.19–1.49 mg/g, total 5.69–18.74 mg/g), and C-GS (Re 0.30–3.45 mg/g, Rh_1_ 0.05–3.42 mg/g, Rd 0.17–1.68 mg/g, total 2.99–19.55 mg/g). Contents of Re and Rf were significantly higher in F-GS than those in C-GS (*p* < 0.05). Using the contents of Re, Rf, or Rb_1_, approximately a half number of cultivated ginseng samples could be identified from ginseng under forest. Contents of Rg_1_, Re, Rg_2_, Rh_1_, as well as the total contents of the seven ginsenosides were highest in ginseng older than 15 years, middle–high in ginseng between 10 to 15 years old, and lowest in ginseng younger than 10 years. Contents of Rg_1_, Re, Rf, Rb_1_, Rg_2_, and the total of seven ginsenosides were significantly related to the growing ages of ginseng (*p* < 0.10). Similarities of chromatographic fingerprints to W-GS were significantly higher (*p* < 0.05) for F-GS (median: 0.824) than C-GS (median: 0.745). A characteristic peak pattern in fingerprint was also discovered for distinguishing three types of ginseng. Conclusively, wild ginseng was remarkably superior to ginseng under forest and cultivated ginseng, with ginseng under forest slightly closer to wild ginseng than cultivated ginseng. The differences among wild ginseng, ginseng under forest, and cultivated ginseng in saponin compositions and contents of ginsenosides were mainly attributed to their growing ages.

## 1. Introduction

**Figure 1:**
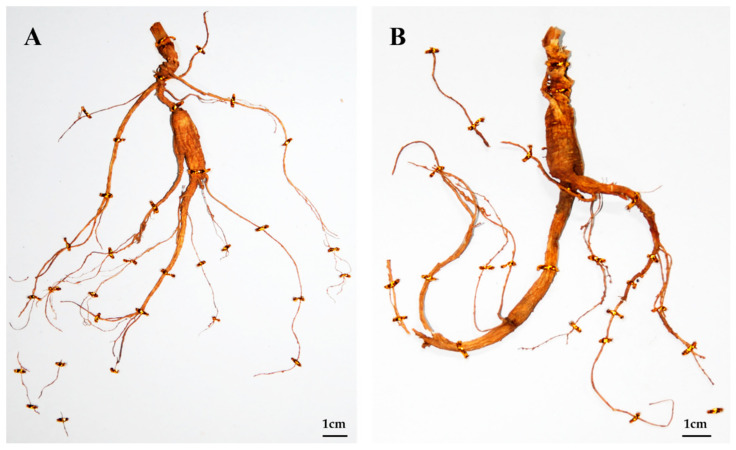
Photographs of two ginseng under forest preserved specimens (**A**) 25-year-old specimen; (**B**) 20-year-old specimen; both were collected in August 2013 and preserved in the Herbarium of Pharmacognosy, School of Pharmaceutical Sciences, Peking University Health Science Center.

Ginseng is a famous and precious traditional Chinese medicine (TCM) derived from the dried root or rhizome of *Panax ginseng* C.A. Mey, which has been used for supplement of primordial *Qi* (the body’s resistance to pathogenic microorganisms and the ability to adjust and adapt [1]) for thousands of years in China. There are two kinds of ginseng recorded in Chinese Pharmacopeia (ver. 2020), which are cultivated ginseng (C-GS), and ginseng under forest (F-GS; ginseng planted in the mountain forest and growing in natural environment similar to wild ginseng (W-GS), and also called mountain cultivated ginseng, Figure 1).

Ginsenosides are the major active constituents in ginseng, which have been demonstrated to have great contributions to the therapeutic effects of ginseng, such as anti-diabetes, cardiovascular effects, neuroprotection, anti-cancer, and so on [2]. Up to now, more than 300 saponins have been identified from cultivated ginseng via HPLC-MS [3,4] and 100 saponins identified from ginseng under forest via UPLC-Q-TOF/MS [4], whereas only 15 saponins (ginsenosides Rg_1_, Re, Rb_1_, Rb_2_, Rf, Rh_1_, Rc, Rd, F_1_, F_2_, F_3_, Ro, and malonyl-Rb_1_, -Rb_2_, and -Rc) [5,6,7] were reported for wild ginseng with the other saponins unknown.

Several methods, including microscopy [8], FT-IR [9], UPLC-QTOF-MS/MS [10], and non-targeted metabolomic analysis [11], have been established for differentiation of F-GS from C-GS. F-GS is usually grown at least 10 years [9,11], whereas C-GS is not older than 6 years. Commonly, F-GS was considered as a substitution for W-GS [11] due to the scarcity and high-demand of W-GS. However, the differences in chromatographic fingerprints or ginsenosides’ contents among W-GS, F-GS, and C-GS are still unclear.

Therefore, in the present study, a simple and applicable strategy, including saponins identification via HPLC-IT-TOF-MS^n^, quantitative analysis of seven ginsenosides by “quantitative analysis of multi-component with single marker” (QAMS) method, fingerprints and characteristic chromatographic analysis via HPLC-DAD, was proposed to discover the differences among W-GS, F-GS, and C-GS in saponin compositions, ginsenosides’ contents and chromatographic features.

## 2. Results

### 2.1. Identification of Saponins in Wild Ginseng, Ginseng under Forest and Cultivated Ginseng with HPLC-IT-TOF-MS^n^

**Figure 2:**
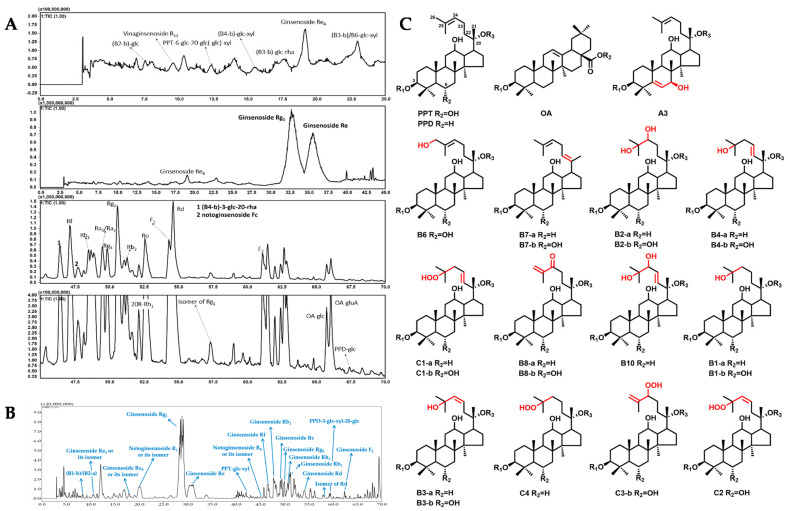
TIC chromatogram of wild ginseng (no. 20) obtained by HPLC-IT-TOF-MS^n^ (**A**), TIC chromatogram of ginseng under forest (no. 18) with 18 of the 21 saponins which could be detected from all three types of ginseng ((**B**); 3 saponins could not be directly marked in TIC chromatogram due to the extremely low intensity), as well as the 15 structure types of saponins tentatively identified from wild ginseng (nos. 19 and 20), ginseng under forest (no. 18), and cultivated ginseng (no. 25; (**C**); R_1_, R_2_, and R_3_ represented sugar residues or substituent groups when not specified under each structure type).

In order to deeply understand the saponin compositions of W-GS and F-GS, the extracts of 100 mg W-GS or F-GS samples were analyzed with HPLC-IT-TOF-MS^n^. A total of 199 saponins were tentatively identified from only 100 mg each of W-GS (no. 19 and no. 20), F-GS (no. 18), and C-GS (no. 25; Table 1), including 36 protopanaxadiol (PPD)-type, 46 protopanaxatriol (PPT)-type, 10 oleanolic acid (OA)-type, and 107 other types of saponins (see Figure 2, Table S1), by comparing MS data with our previous research [12] and other published data [3,4,11,13,14,15,16,17,18,19,20,21].

As shown in Table 1, among the 199 saponins identified, a total of 177 saponins were detected from W-GS type including 161 from 100-year-old W-GS (no. 20) and 112 saponins from 50-year-old W-GS (no. 19). A total of 56 saponins were detected from 25-year-old F-GS (no. 18), with 60 saponins detected from C-GS (no. 25). For W-GS, 162 of the 177 saponins identified were found in W-GS for the first time, with only 15 ginsenosides (Rg_1_, Re, Rb_1_, Rb_2_, Rf, Rh_1_, Rc, Rd, F_1_, F_2_, F_3_, Ro, and malonyl-Rb_1_, -Rb_2_, -Rc) previously reported [5,6,7]. Additionally, 16 potential new compounds (neither found in SciFinder nor previously reported) were discovered (Table 2), including seven detected in W-GS no. 20, 10 detected from W-GS no. 19, one detected from F-GS no. 18, and six detected from C-GS no. 25, which needed to be further confirmed by separation of high-purity substances. Moreover, 21 of the 199 saponins were commonly detected from all of the three ginseng types (W-GS, F-GS, and C-GS).

**Figure 3:**
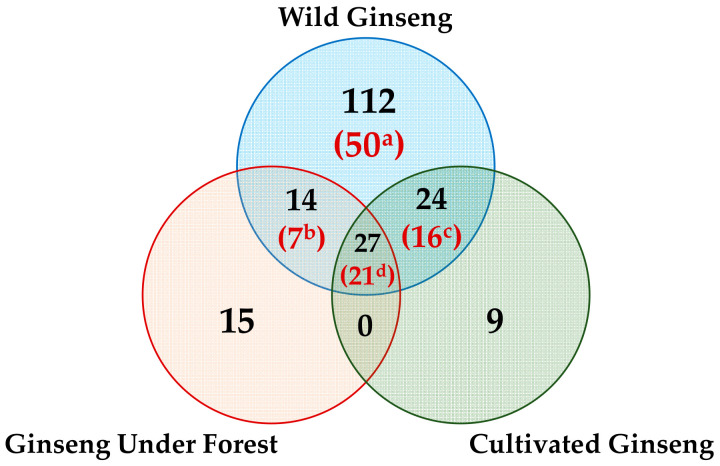
Saponins identified from each ginseng type only, two types of ginseng, or all the three types of ginseng via HPLC-IT-TOF-MS^n^ (wild ginseng was samples nos. 19 and 20 combined, ginseng under forest was sample no. 18, and cultivated ginseng was sample no. 25; a, 112 saponins detected from at least one of the two W-GS with 50 saponins detected from both the two W-GS; b, 14 saponins detected from F-GS and at least one of the two W-GS with seven saponins detected from F-GS and both the two W-GS; c, 24 saponins detected from C-GS and at least one of the two W-GS with 16 saponins detected from C-GS and both the two W-GS; d, 27 saponins detected from C-GS, F-GS, and at least one of the two W-GS with 21 detected from C-GS, F-GS, and both the two W-GS).

As shown in Figure 3, there were 112, 15, and 9 saponins that could only be detected from W-GS (no. 20 and/or no. 19), F-GS (no. 18), and C-GS (no. 25), respectively. There were 50 saponins detected from both the two W-GS but not from F-GS or C-GS, including 23 saponins found in ginseng for the first time, which might imply that these 23 saponins only generated in ginseng over 50 years old and might be the characteristics for ginseng older than 50 years to distinguish from ginseng younger than 25 years.

Almost all the saponins identified via HPLC-IT-TOF-MS^n^ were glucosides. A total of 48 rhamnosides, including 39 from W-GS no. 20, 29 from W-GS no. 19, eight from F-GS no. 18, and 11 from C-GS no. 25, were identified. Similarly, a total of 42 xylosides, including 39 from W-GS no. 20, 27 from W-GS no. 19, 17 from F-GS no. 18, and 16 from C-GS no. 25, were identified (Table 1). Only three saponins (two from both the two W-GS and one from W-GS no. 20) were found to be both rhamnosides and xylosides.

### 2.2. Potential New Compounds Found in Wild Ginseng, Ginseng under Forest and Cultivated Ginseng via HPLC-IT-TOF-MS^n^

Sixteen potential new compounds were tentatively identified from ginseng via HPLC-IT-TOF-MS^n^ by comparing MS^n^ data to previous publications [3,4,11,12,13,14,15,16,17,18,19,20,21] and searching possible structures in SciFinder.

Potential new compound with retention time of 4.147 min in C-GS no. 25 (identification no. 2 in Table 2) showed [M − H]^−^ at *m/z* 1027.5105 with its molecular formula tentatively predicted as C_51_H_80_O_21_ (Diff: −1.36 ppm). The fragment ion at *m/z* 847.4492 was generated from *m/z* 1027.5105 by neutral loss of 180.0613 Da [C_6_H_12_O_6_, a glucose (glu), Diff: 11.60 ppm]. In MS^3^, the fragment ion at *m/z* 701.3909 was generated from *m/z* 847.4492 by neutral loss of 146.0583 Da [a rhamnosyl (rha), Diff: 2.68 ppm], and *m/z* 521.3270 was generated from *m/z* 701.3909 by neutral loss of 180.0639 Da [a glucose (glu), Diff: 2.84 ppm]. The fragment ion at *m/z* 521.3270 was predicted as C_33_H_45_O_5_ (Diff: −0.38 ppm), which was the [M − H]^−^ of the aglycone dehydrated by 2 H_2_O. The aglycone of this potential new compound was tentatively predicted as C_33_H_50_O_7_, which was a potential new aglycone for ginseng, with this compound tentatively predicted as C_33_H_50_O_7_-glc-glc-rha (Figure S7 in Supplemental Materials).

Potential new saponin with retention time of 41.082 min in W-GS no. 20 (identification no. 14 in Table 2) showed [M − 2H]^2−^ at *m/z* 561.2898 with its molecular formula tentatively predicted as C_54_H_92_O_24_ (Diff: −3.39 ppm). In MS^2^, the fragment ion at *m/z* 961.5367 should be generated from [M − H]^−^ (*m/z* 1123.5874) neutral loss of 162.0507 Da [a glucosyl (glc), Diff: 13.10 ppm], *m/z* 799.4831 was generated from *m/z* 961.5367 by neutral loss of 162.0536 Da (a glc, Diff: 4.79 ppm), *m/z* 781.4794 was generated from *m/z* 961.5367 by neutral loss of 180.0573 Da (a glu, Diff: 33.81 ppm), *m/z* 637.4221 was generated from *m/z* 799.4831 by neutral loss of 162.0610 Da (a glc, Diff: 50.46 ppm), *m/z* 475.3830 was generated from *m/z* 637.4221 by neutral loss of 162.0391 Da (a glc, Diff: 84.68 ppm), and *m/z* 375.2964 was generated from *m/z* 475.3830 by neutral loss of 100.0866 Da (C_6_H_12_O, the side chain at C20 of the aglycone, Diff: 22.13 ppm). The neutral loss of 100 Da was the characteristic neutral loss of aglycone B4-a in Figure 2C [3]. Therefore, it was tentatively identified as (B4-a)-6-glc-20-glc(-glc)-glc (Figure S17 in Supplemental Materials), the locations and orders of sugar residues were not certain. MS^n^ data of the other 14 potential new saponins could be found in Table 2.

**Table 2:** Sixteen potential new compounds tentatively identified from wild ginseng (W-GS), ginseng under forest (F-GS), and cultivated ginseng (C-GS) via HPLC-IT-TOF-MS^n^.

	RT (min)	Molecular Formula	Measured (*m/z*)	Predicted (*m/z*)	Error (ppm)	Ion	n-ESI-MS^n^ Data	Identification	Source
1	3.938	C_52_H_88_O_23_	1079.5628	1079.5644	−1.48	[M − H]^−^	MS^2^[1079.5627]: 962.5320(100), 961.5308(76.47), 781.4758(11.26) MS^3^[1079.5627→961.5361]: 799.4771(4.42), 781.4756(100), 635.4042(23.49), 617.4012(5.15)	(B3-b)-glc-glc-rha-C_4_H_6_O_4_	W-GS (no. 19)
2	4.147	C_51_H_80_O_21_	1027.5105	1027.5119	−1.36	[M − H]^−^	MS^2^[1027.5104]: 847.4492(100) MS^3^[1027.5104→847.4492]: 701.3909(100), 521.3270(57.60), 491.2823(5.26)	Unknown(C_33_H_50_O_7_)-glc-glc-rha	C-GS (no. 25)
3	6.350	C_48_H_80_O_20_	1021.5112	1021.5225	−11.06	[M + HCOO]^−^	MS^2^[1021.5113]: 975.5109(100), 815.4821(11.98) MS^3^[1021.5113→975.5104]: 815.4704(47.05), 669.4099(100) MS^4^[1021.5113→975.5104→669.4099]: 507.3565(100)	(C3-b)/(C1-b)/(B10-b)-glc-C_6_H_8_O_5_-rha	W-GS (no. 19 and 20)
4	6.970	C_45_H_68_O_16_	863.4456	863.4435	2.43	[M + HCOO]^−^	MS^2^[863.4454]: 717.3812(100), 537.3186(31.79), 391.2789(14.69)	Unknown(C_33_H_48_O_7_)-glc-rha	C-GS (no. 25)
5	9.168	C_45_H_70_O_17_	881.4549	881.4540	1.02	[M + HCOO]^−^	MS^2^[881.4550]: 823.4218(17.73), 735.4004(35.88), 677.3571(97.45), 555.3343(15.43), 515.3014(8.70), 497.2947(100)	Unknown(C_30_H_44_O_7_)-glc-rha-C_3_H_6_O	C-GS (no. 25)
6	9.447	C_35_H_40_O_7_	617.2671	617.2756	−13.77	[M + HCOO]^−^	MS^2^[617.2656]: 571.2617(100) MS^3^[617.2656→571.2617]: 439.1954(100), 277.1775(100)	Unknown(C_30_H_32_O_3_)-xyl	W-GS (no. 19) and F-GS (no. 18)
7	11.687	C_54_H_84_O_26_	573.2556	573.2553	0.52	[M − 2H]^2−^	MS^2^[573.2557(2)]: 985.4663(100) MS^3^[573.2557(2)→985.4663]: 643.3430(100), 463.2941(23.54)	Unknown(C_30_H_44_O_6_)-glc-glc-glc-glc	C-GS (no. 25)
8	15.098	C_45_H_70_O_16_	865.4600	865.4591	1.04	[M − H]^−^	MS^2^[865.4600]: 719.3996(93.02), 539.3384(100), 509.2889(5.33)	Unknown(C_33_H_50_O_7_)-glc-rha	C-GS (no. 25)
9	19.317	C_48_H_80_O_18_	989.5305	989.5327	−2.22	[M + HCOO]^−^	MS^2^[989.5299]: 943.5149(100) MS^3^[989.5299→943.5148]: 763.4574(100), 617.3911(15.50)	PPD-gluA-glc-rha	W-GS (no. 19)
10	20.045	C_36_H_64_O_10_	701.4468	701.4482	−2.00	[M + HCOO]^−^	MS^2^[701.4473]: 655.4324(100), 493.3847(58.09) MS^3^[701.4473→654.4171]: 493.3859(100), 392.2902(25.12)	(B1-b)-glc	W-GS (no. 19, 20) and C-GS (no. 25)
11	20.270	C_42_H_74_O_14_	843.4735	843.4748	−1.54	[M + HCOO]^−^	MS^2^[843.4733]: 799.4767(100), 617.4006(18.51) MS^3^[843.4733→799.4764]: 653.4222(44.40), 635.4042(44.40), 491.3685(88.79), 391.2842(100)	Unknown(C_31_H_52_O_7_)-glc-rha	W-GS (no. 19 and 20)
12	26.555	C_38_H_66_O_13_	775.4551	775.4485	8.51	[M + HCOO]^−^	MS^2^[775.4549]: 729.4399(100) MS^3^[775.4549→730.4454]: 583.3904(33.33), 421.3375(100)	Unknown(C_26_H_46_O_4_)-glc-rha	W-GS (no. 19)
13	40.932	C_41_H_68_O_13_	813.4639	813.4642	−0.37	[M + HCOO]^−^	MS^2^[813.4639]: 767.4578(100), 635.4058(34.42), 489.3553(4.17)	(B8-b)-rha-xyl	W-GS (no. 19 and 20)
14	41.082	C_54_H_92_O_24_	561.2898	561.2917	−3.39	[M − 2H]^2−^	MS^2^[561.2900(2)]: 961.5367(89.98), 799.4831(88.49), 781.4794(100), 637.4221(54.76), 475.3830(47.19), 375.2964(7.86)	(B4-a)-6-glc-20-glc(-glc)-glc	W-GS (no. 20)
15	44.095	C_39_H_68_O_14_	805.4600	805.4591	1.12	[M + HCOO]^−^	MS^2^[805.4597]: 759.4501(100) MS^3^[805.4597→759.4501]: 597.4013(31.25), 435.3427(100), 375.2780(7.99) MS^4^[805.4597→759.4501→597.4014]: 435.3394(100)	Unknown(C_27_H_48_O_4_)-glc-glc	W-GS (no. 19 and 20)
16	45.002	C_38_H_64_O_14_	743.4226	743.4223	0.40	[M − H]^−^	MS^2^[743.4225]: 597.3617(70.84), 435.3087(100), 417.2935(7.98)	Unknown(C_26_H_44_O_5_)-glc-rha	W-GS (no. 19)

RT, retention time; glc, glucosyl; rha, rhamnosyl; xyl, xylosyl; more detailed HPLC-MS information were provided as Supplemental Materials Table S1.

### 2.3. Quantitative Analysis of Seven Ginsenosides in Ginseng with “Quantitative Analysis of Multi-Component with Single Marker” (QAMS) Method

Using the QAMS method established in our previous study [22], the contents of seven ginsenosides, including ginsenosides Rg_1_, Re, Rf, Rb_1_, Rg_2_, Rh_1_, and Rd, were determined referring to Rg_1_ and compared with *t*-test. As a result, the contents of the seven ginsenosides as well as their total contents (i.e., the summation of contents of the seven ginsenosides) were the highest in W-GS (55.51 mg/g for no. 20 and 50.52 mg/g for no.19), which were remarkably higher than those in F-GS (ranging from 5.56 mg/g to 18.74 mg/g) and C-GS (ranging from 2.99 mg/g to 19.55 mg/g; see Table 3 and Table 4). Only contents of Re and Rf in F-GS with Re ranging from 1.22–3.50 mg/g and Rf ranging from 0.29–1.19 mg/g were higher than those (Re: 0.03–3.45 mg/g; Rf: 0.14–0.95 mg/g) in C-GS (*p* < 0.05).

**Figure 4:**
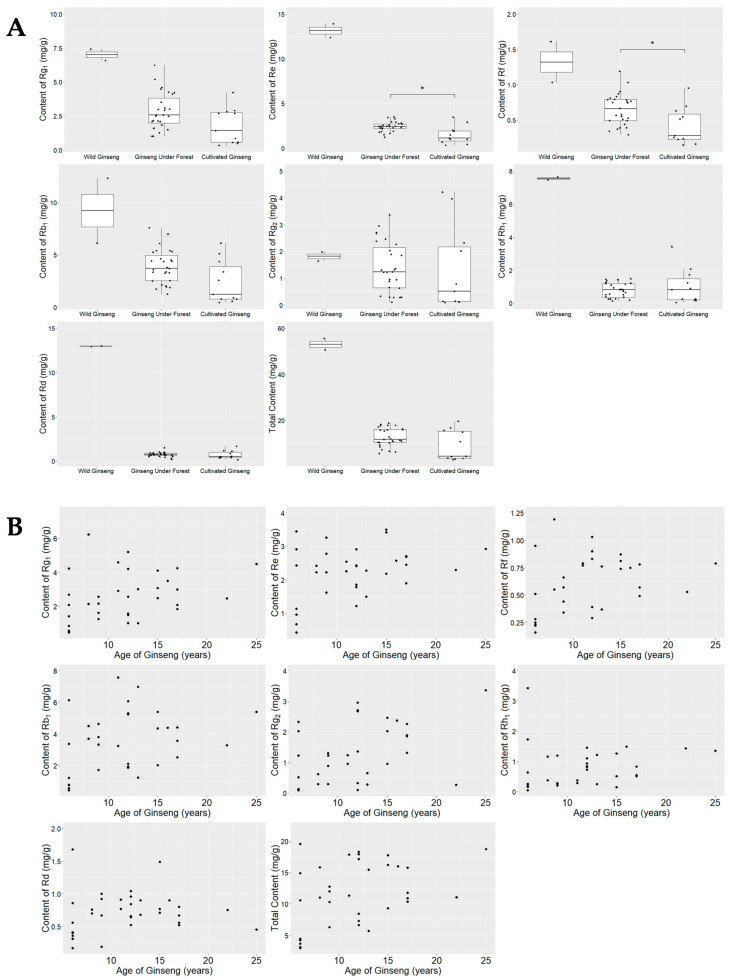
Comparison of the contents of seven ginsenosides and their total content in different types of ginseng samples using *t*-test ((**A**); wild ginseng *n* = 2, ginseng under forest *n* = 27, cultivated ginseng *n* = 11; * *p* < 0.05), and the scattered plots for contents of the seven ginsenosides and their total content in ginseng under forest and cultivated ginseng with certain growing ages ((**B**); *n* = 34; contents of the seven ginsenosides in six samples—nos. 18, 19, 36–39 were not plotted due to the uncertainty of growing ages).

As shown in Table 4 and Figure 4A, the content ranges of Rg_1_, Re, Rf, Rh_1_, Rd, and the total content of all seven ginsenosides for C-GS samples were remarkably lower than the contents in the two W-GS samples. Additionally, the median contents of Rb_1_ and Rg_2_ for C-GS samples were much lower than the contents of Rb_1_ and Rg_2_ for W-GS. These results indicated that there were remarkable difference between C-GS and W-GS in contents of these ginsenosides. It was also found that the contents of Rg_1_, Re, Rh_1_, Rd and the total content of the seven ginsenosides for F-GS samples were remarkably lower than those in W-GS samples, besides the median contents of Rf, Rb_1_, and Rg_2_ for F-GS were much lower than the contents in W-GS samples, which indicated that F-GS fell in between W-GS and C-GS and were much closer to C-GS in the contents of these ginsenosides. The median contents of Re, Rf, Rb_1_, and the median total contents for C-GS samples were lower than the lower limits of those for F-GS samples, which meant that those contents of a half number C-GS samples were remarkably lower than the lower limits of the content ranges for F-GS. This result implied that there were some degree of difference between F-GS and C-GS in the contents of Re, Rf, Rb_1_ and total contents of seven ginsenosides, which could be applied to distinguish C-GS from F-GS when those contents were remarkably below the corresponding content lower limits of F-GS (Re 1.22 mg/g, Rf 0.29 mg/g or Rb_1_ 1.24 mg/g).

As shown in Figure 4B, the lowest contents of Rg_1_, Re, Rf, Rb_1_, and the total contents of the seven ginsenosides were all from 6-year-old ginseng. As shown in Table 5, among all the F-GS and C-GS samples with known growing ages (Table 8), the mean total contents of the seven ginsenosides (and the range) were 13.79 mg/g (9.31–18.74 mg/g) for F-GS with ages over 15 years, 13.02 mg/g (5.69–18.30 mg/g) for F-GS with ages between 10 years and 15 years, and 9.41 mg/g (2.99–19.55 mg/g) for F-GSs and C-GSs with ages under 10 years with the one C-GS sample (no. 25) with extremely high contents (total contents 19.55 mg/g; Table 3). Using the correlation analysis (rcorr function) in R with hmisc package, it was found that the contents of Rg_2_, Re, and the total contents of the seven ginsenosides were related to the growing ages of ginseng with correlation coefficients (and *p*-value) at 0.45 (0.0082), 0.37 (0.0297), and 0.38 (0.0247) for Rg_2_, Re, total contents, respectively (Table 6). The contents of Rg_1_, Rf, and Rb_1_ were found to be related to the growing ages with relatively weak correlations (correlation coefficients 0.29–0.33, *p* < 0.10). The results indicated that older ages might lead to higher ginsenoside accumulation.

### 2.4. Fingerprints Comparison of Wild Ginseng, Ginseng under Forest, and Cultivated Ginseng

**Figure 5:**
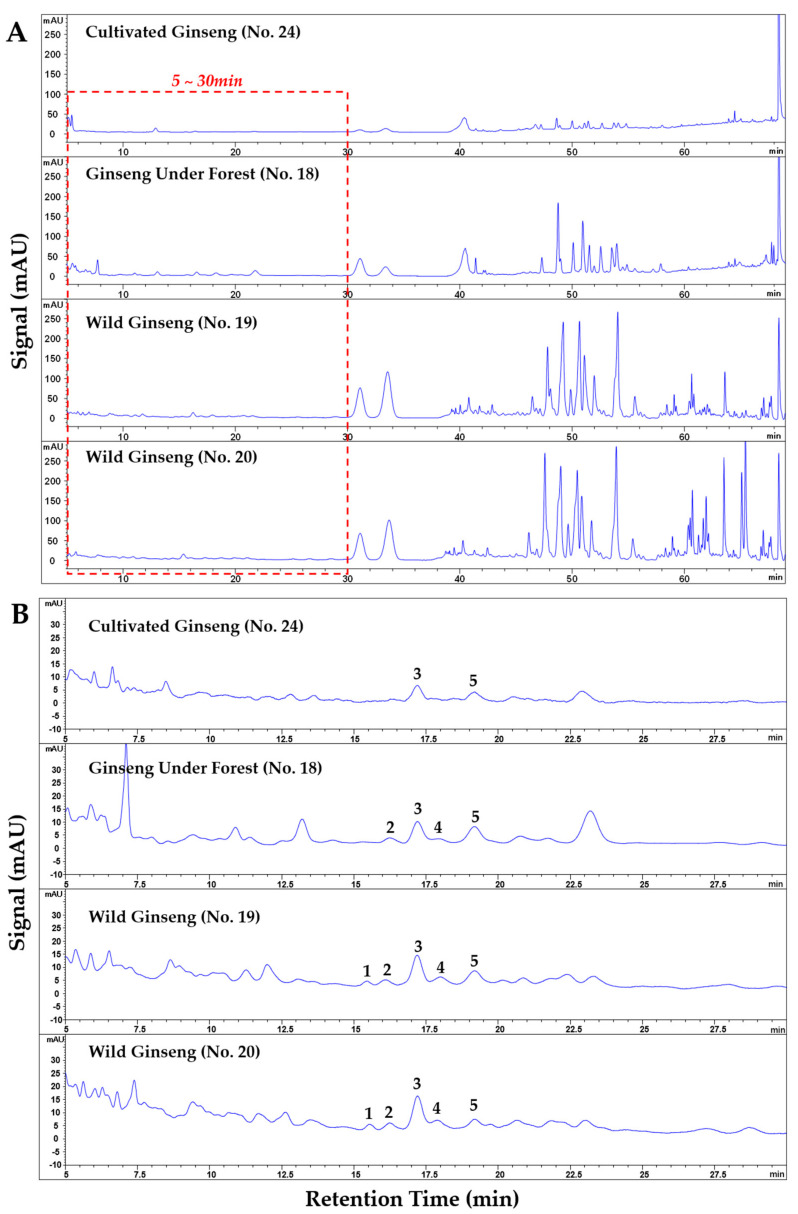
Chromatograms (retention time: 5–70 min) of cultivated ginseng (no. 24), ginseng under forest (no. 18), wild ginseng (no. 19, ~50 years old; no. 20, ~100 years old; (**A**)), as well as their zoomed-in chromatograms (5–30min) labeled with the five characteristic peaks in Section 2.5 [(**B**); 1, (B4-b)-glc-xyl; 2, (B3-b)-glc-rha; 3, ginsenoside Re_4_ (or its isomer); 4, 25-hydroxy-ginsenoside Rg_2_; 5, (B3-b)/B6-glc-xyl tentatively identified via HPLC-IT-TOF-MS^n^].

**Figure 6:**
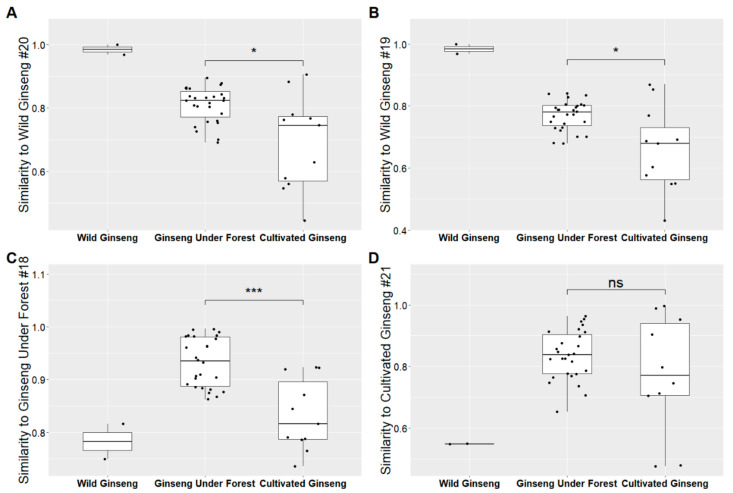
Similarities of HPLC-DAD fingerprints of 40 ginseng samples to wild ginseng (no. 20, **A**; no. 19, **B**), ginseng under forest (no. 18, **C**), and cultivated ginseng (no. 21, **D**; ns—no significance, * *p* < 0.05, *** *p* < 0.001 between groups).

The fingerprints of all the forty ginseng samples (see Figure 5A) were obtained by HPLC-DAD with our previous method [23]. The retention time of all the fingerprints were normalized according to 10 common peaks (including ginsenoside Rg_1_, Re, Rf, Rb_1_, Rc, Rg_2_, and the other four unknown peaks) via Similarity Evaluation System (Figure S26 in Supplemental Materials). The similarities of chromatographic fingerprints of the forty samples were analyzed within the retention time window of 5–70 min using W-GS (no. 20, about 100 years old), W-GS (no. 19, about 50 years old), F-GS (no. 18, 25 years old), and C-FS (no. 21, 6 years old) as references, respectively. The results showed that, the similarities of fingerprints of F-GS samples to both W-GS samples were significantly higher than those of C-GS samples (*p* < 0.05), with the median similarities of 0.824 for F-GS samples, 0.745 for C-GS samples to W-GS no. 20, and 0.781 for F-GS samples, 0.68 for C-GS samples to W-GS no. 19, respectively. When referring to a F-GS sample (no. 18, 25 years old), the similarities of fingerprints of F-GS samples were still significantly higher than those of C-GS samples, with the median similarities of 0.935 for F-GS samples and 0.816 for C-GS samples (see Figure 6).

### 2.5. Differentiation of Wild Ginseng, Ginseng under Forest and Cultivated Ginseng with Characteristic Peak Pattern of Chromatogram

**Figure 7:**
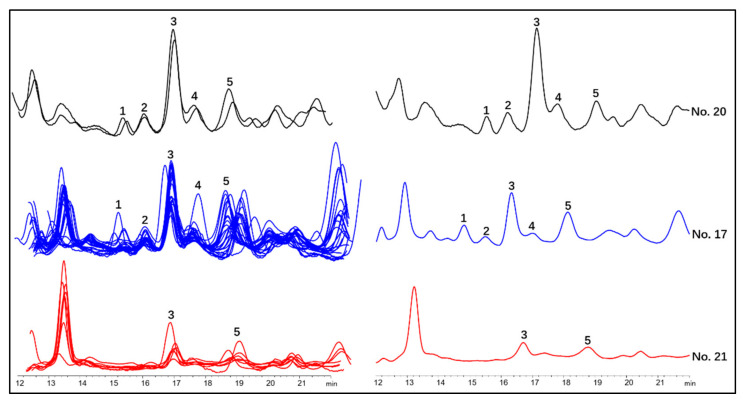
The chromatographic features of cultivated ginseng (red), ginseng under forest (blue), and wild ginseng (black) between the retention time 14–20 min. (Left: the overlapped graphs of all the cultivated, ginseng under forest, and wild ginseng samples; right: the chromatograms of cultivated ginseng no. 21, ginseng under forest no. 17, and wild ginseng no. 20. Characteristic peaks were tentatively identified via HPLC-IT-TOF-MS^n^ as: 1, (B4-b)-glc-xyl; 2, (B3-b)-glc-rha; 3, ginsenoside Re_4_ (or its isomer); 4, 25-hydroxy ginsenoside Rg_2_; and 5, (B3-b)/(B6)-glc-xyl).

For further discovering characteristic patterns of chromatographic fingerprint and distinguishing F-GS from C-GS, the correlation between peak area of each single chromatographic peak within the retention time of 5–70 min with the similarity of chromatographic fingerprint of each ginseng sample to two W-GS samples (no. 19 and no. 20) was analyzed using R with hmisc package. The results showed that there were strong correlations (correlation coefficient > 0.4 and *p* < 0.05) between similarities of chromatographic fingerprints and the peak areas of nine peaks (Table 7). Within retention time of 32–50 min, four of the nine peaks were tentatively identified as Rg_1_, Re, Rf, Rb_1_ with standard substances, with other two tentatively identified as notoginsenoside Fc and ginsenoside F_3_ by HPLC-IT-TOF-MS^n^. The other three peaks, within retention time 15–18 min, were tentatively identified as (B4-b)-glc-xyl, (B3-b)-glc-rha, and Ginsenoside Re_4_ (or its isomer). This result implied that there might be featured pattern within 15–18 min. The results of the further direct observation from chromatograms showed that between retention time 14 min to 20 min, there was a characteristic peak group consisting of five peaks, including (B4-b)-glc-xyl, (B3-b)-glc-rha, ginsenoside Re_4_ (or its isomer), 25-hydroxy-ginsenoside Rg_2_, and (B3-b)/(B6)-glc-xyl (Figure 4B and Figure 7). When defining peaks with signal-to-noise ratio over 10 as detected, both the 100-year-old and 50-year-old W-GS samples contained all the five peaks in the group; most the F-GS samples (23/27, except F-GS nos. 8, 29, 34, and 35 in Table 8) contained at least four peaks in the characteristic group; C-GS only contained three peaks at most (see Table S3). This characteristic peak pattern could be used for identifying F-GS from C-GS.

## 3. Discussion

It has been reported that 334 saponins were identified from 300 g of *Panax ginseng* sample by LC-MS [3], which is not even applicable to F-GS or W-GS due to their rarity. Therefore, a semi-micro sample preparation method was used in the present study, which was established for the analysis of *Panax notoginseng* in our pervious study [24]. Finally, 199 saponins were tentatively identified from only 100 mg (as 1/3000 as a sample amount in the above publication [3]) of W-GS, F-GS, and C-GS samples with 16 potential new compounds discovered. The present study also reported 177 saponins from wild ginseng, including 162 saponins reported from wild ginseng for the first time, and 11 potential new compounds. For ginsenosides besides glucosides, 41 rhamnosides and 41 xylosides were detected from W-GS (nos. 19 and 20 combined), in comparison to eight rhamnosides and 17 xylosides from F-GS (no. 18), and 11 rhamnosides and 16 xylosides from C-GS (no. 25), respectively. The results implied that all types of ginsenosides (including glucosides, rhamnosides, and xylosides) gradually accumulated along with the prolongation of growing ages. Fifty saponins only detected from both the two W-GS, 23 saponins of which found in ginseng for the first time might be the characteristic markers for wild ginseng (ginseng older than 50 years).

Among the 16 potential new compounds tentatively identified via HPLC-IT-TOF-MS^n^, 10 of them were found to contain potential new aglycones (nine), the structures of which need to be further demonstrated with purified substances separated from ginseng. Four of the nine potential new aglycones could be only found in C-GS (no. 25), including C_33_H_50_O_7_ (2), C_33_H_48_O_7_, C_30_H_44_O_7_, and C_30_H_44_O_6_, with the other 5 potential new aglycones found in W-GS (no. 19 and/or 20) and F-GS, including C_30_H_32_O_3_, C_31_H_52_O_7_, C_26_H_46_O_7_, C_27_H_48_O_4_, and C_26_H_44_O_5_, but not in C-GS. The other six potential new compounds were consisted of known aglycones including B3-b, (C3-b)/(C1-b)/(B10-b), B1-b, B8-b, B4-a, and PPD.

Among the three types of ginseng samples, wild ginseng had the highest diversity of saponins and the highest contents of seven ginsenosides (except Rg_2_). The orders of median total contents and mean total contents of the seven ginsenosides in the three ginseng type both were W-GS (50.52 mg/g, 55.51 mg/g) > F-GS (median 11.80 mg/g, mean 14.21 mg/g) > C-GS (median 4.46 mg/g, mean 9.06 mg/g), although only contents of Re and Rf in F-GS (Re 1.22–3.50 mg/g, Rf 0.29–1.19 mg/g) were significantly higher (*p* < 0.05) than those in C-GS (Re 0.30–3.45 mg/g, Rf 0.14–0.95 mg/g). It was, for the first time, the contents of ginsenosides were compared among W-GS, F-GS, and C-GS, the results of which showed that the contents of Re, Rh_1_, and Rd in W-GS were remarkably higher than those in F-GS and C-GS, which might be attributed to the long growing period for W-GS (ages estimated as 50 years for W-GS no. 19 and 100 years for W-GS no. 20, Table 8). By means of correlation analysis, it was found that the contents of Rg_2_, Re, and the total contents of the seven ginsenosides were related to growing ages of ginseng (correlation coefficient: 0.37–0.45, *p* < 0.05), with contents of Rg_1_, Rf, and Rb_1_ weakly related to growing ages (correlation coefficient: 0.29–0.33, *p* < 0.10). These results all implied that the difference in contents of ginsenosides among different ginseng types might be related to their growing periods. However, more ginseng samples with different ages might be needed to confirm the hypothesis of relationship between ginsenosides and growing ages. Additionally, in the present study, it was also found that the contents of Re, Rh_1_, or Rd could be used for identification of W-GS from F-GS or C-GS.

The similarities of chromatographic fingerprints of F-GSs to both wild ginseng samples (0.824 to W-GS no. 20, 0.781 to W-GS no. 19) were significantly higher than those of cultivated ginseng (0.745 to W-GS no. 20, 0.68 to W-GS no. 19), which implied that F-GS was more similar to W-GS than C-GS taking the whole chemical profiling of saponins in ginseng into consideration, although no remarkable difference was found between F-GS and C-GS in saponins identification via HPLC-IT-TOF-MS^n^ or contents of seven ginsenosides by QAMS method. Taking all results above together, it showed that F-GS was slightly closer to W-GS in comparison with C-GS quantitatively and qualitatively via HPLC-DAD.

Although 17 potential age-dependent markers have been reported to differentiated F-GS and C-GS by OPLS-DA [11], it was not well illustrated that how to use these markers to identify F-GS from C-GS. Therefore, in order to quickly identify characteristic chromatographic fingerprint, a correlation analysis was conducted, and finally a characteristic chromatographic fingerprint peak pattern consisting of five peaks was discovered within the retention time of 15–20 min, which could be used to differentiate F-GS from C-GS by comparing the number of peaks (≥4 peaks for F-GS and ≤3 peaks for C-GS).

The present research could also provide a simple, effective and applicable strategy for the first time to comprehensively evaluate the quality of ginseng, which included a fingerprints analysis combined quantitative analysis method for quality evaluation, and a correlation-assisted characteristic chromatographic profile identification for distinguishing ginseng under forest and cultivated ginseng. Additionally, the strategy established in the present study could be used in the quality evaluation or comparison researches of other TCMs as well.

## 4. Materials and Methods

### 4.1. Chemicals and Reagents

Methanol and acetonitrile (HPLC grade) were obtained from Thermo Fisher Scientific (Fair Lawn, NJ, USA). Deionized water was purified using a Milli-Q Water Purification System (Millipore, Billerica, MA, USA). Formic acid (HPLC grade) was purchased from ROE Scientific (Main St, Newark, DE, USA). Methanol and n-butanol (analytical grade) were purchased from Beijing chemical factory. Reference substances of ginsenosides Rg_1_, Re, Rf, Rb_1_, Rg_2_, and Rd were purchased from the Department of Organic Chemistry, College of Health Science, Jilin University. Reference substance of ginsenosides Rh_1_ was purchased from Chengdu Must Bio-Technology Co., LTD (Chengdu, China). Purities of all the reference substances were higher than 98%.

### 4.2. Plant Materials

**Figure 8:**
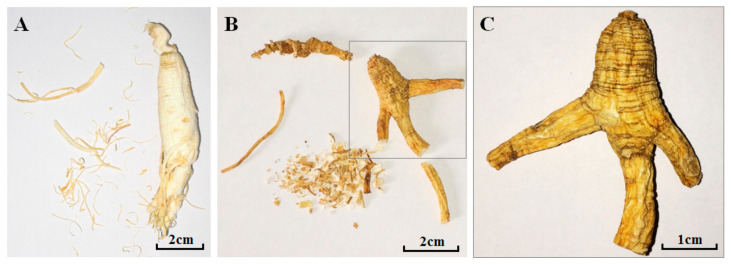
Photographs of cultivated ginseng ((**A**), no. 21, 6-year-old) and ginseng under forest ((**B**) no. 27, 9-year-old) and its amplified main root part with dark yellow color and deep cross striation ((**C**) no. 27).

In the present study, a total of forty ginseng samples (all contain root and rhizome; Figure 8) were used. Twenty-seven of all the samples (nos. 1–26 and no. 40 in Table 8) including seven C-GS, two W-GS and eighteen F-GS samples were provided and identified by one of the authors—Professor Yu-Shu Huo (University of Texas, San Antonio, TX USA), which were collected from Jilin province and had already been pulverized into powder before the samples assigned to our lab. Nine of the F-GS (nos. 27–35 in Table 8) samples were given as gifts and identified by one of the authors Prof. Da-Qing Zhao (Changchun University of Chinese Medicine, Changchun, China). The other four C-GS samples (nos. 36–39 in Table 8) were collected from different drug stores in Beijing and were identified by another author—Professor Shao-Qing Cai (Peking University, Beijing, China). Samples no. 27–39 were pulverized into powder, then completely sifted through a sieve with the hole diameter of 0.45 mm. Voucher specimens were deposited in the Herbarium of Pharmacognosy, School of Pharmaceutical Sciences, Peking University Health Science Center (Beijing, China).

**Table 8:** Detail information of forty ginseng samples used in the present study.

No.	Type	Age/Year	Production Area	No.	Type	Age/Year	Production Area
1	F-GS ^1^	12	Xintunzi village	21	C-GS ^5^	6	Fusong village
2	F-GS	12	Xintunzi village	22	C-GS	6	Fusong village
3	F-GS	12	Xintunzi village	23	C-GS	6	Fusong village
4	F-GS	12	Xintunzi village	24	C-GS	6	Fusong village
5	F-GS	12	Xintunzi village	25	C-GS	6	Fusong village
6	F-GS	22	Yanjiang village	26	C-GS	6	N
7	F-GS	17	Yanjiang village	27	F-GS	9	N
8	F-GS	12	Yanjiang village	28	F-GS	9	N
9	F-GS	16	Yanjiang village	29	F-GS	9	N
10	F-GS	15	Donggang village	30	F-GS	13	N
11	F-GS	8	Donggang village	31	F-GS	11	N
12	F-GS	9	Ningkuandian county	32	F-GS	11	N
13	F-GS	6	Ningkuandian county	33	F-GS	8	N
14	F-GS	13	Xintunzi village	34	F-GS	15	N
15	F-GS	17	Xintunzi village	35	F-GS	15	N
16	F-GS	17	Xintunzi village	36	C-GS	N	Jilin province
17	F-GS	17	Yanjiang village	37	C-GS	N	Jilin province
18	F-GS	25	Yanjiang village	38	C-GS	N	Ji’an city
19	W-GS ^2^	~50 ^3^	N ^4^	39	C-GS	N	N
20	W-GS	~100 ^3^	N	40	C-GS	6	Fusong village

Xintunzi, Yanjiang, Donggang, and Fusong villages all belongs to Fusong county, Jilin Province; ^1^ ginseng under forest; ^2^ wild ginseng; ^3^ estimated age; ^4^ unknown; ^5^ cultivated ginseng; Samples nos. 1–18 and no. 26 were collected in 2011; Samples nos. 21–25 and no. 40 were collected in 2012; Samples nos. 27–35 were collected in 2013; The collection time of samples nos. 19, 20, 36–40 was unknown.

### 4.3. Semi-Micro Sample Preparation Method for Ginseng Sample Preparation

As F-GS samples were very precious and had very small sample size, a previous published semi-micro sample preparation method was used with minor modification [24]. Each of the fine powdered samples (100 mg) was accurately weighed and extracted with 10 mL of n-butanol saturated with water by an ultrasonicator (250 W) for 60 min. After cooling, the extracts were filtered, and the filtrate was evaporated to dryness under vacuum. The residue was then dissolved in an appropriate volume of methanol and further diluted to 1.00 mL. The solution was centrifuged at 10,000× *g* for 10 min prior to HPLC-DAD and HPLC-IT-TOF-MS^n^ analysis. Due the rarity of W-GS and F-GS samples, each sample was prepared once and analyzed twice by HPLC-DAD and once by HPLC-IT-TOF-MS^n^.

### 4.4. Chromatographic Conditions

Considering the chemical similarity between *Panax ginseng* and *Panax notoginseng*, a chromatographic method for the detection of ginsenosides in notoginseng established in our previous study were used [23]. An Agilent 1200 (Agilent, Santa Clara, CA, USA) LC system equipped with a binary gradient pump, auto-sampler and DAD detector was used. The HPLC analysis was conducted using a reversed-phase column (Luna ODS-2, 250 × 4.6 mm, 5 μm). The binary gradient elution system consisted of solvent A (0.005% formic acid/water solution) and solvent B (0.005% formic acid/acetonitrile solution). Separation was achieved using the following elution gradient: 0–35 min, 21% B; 35–36 min, 21–30% B; 36–55 min, 30–40% B; 55–65 min, 40–85% B; 65–80 min, 85–100% B; 80–100 min, 100% B. The column temperature was set at 25 °C. The flow rate was 1 mL/min. A volume of 10 μL of each sample was injected into HPLC-DAD system. The DAD scanning range was from 190 to 400 nm.

### 4.5. HPLC-IT-TOF-MS^n^ and “5-Point Screening” Method

HPLC-IT-TOF-MS^n^ analysis was implemented on a Shimadzu HPLC system coupled to an IT-TOF mass spectrometer with an ESI interface (Shimadzu, Kyoto, Japan), using the same chromatographic condition with HPLC-DAD analysis (see Section 4.4). The MS was operated in both positive ion (PI) and negative ion (NI) modes with data acquisition range of *m/z* 200–1800 (MS^1^) and *m/z* 75–1800 (MS^2^, MS^3^, and MS^4^, data dependent program). All data were acquired by LCMSsolution software Ver. 3.61. The flow rate was 0.2 mL/min. The heat block and curved desolvation line temperature was 250 °C. The nebulizing nitrogen gas flow was 1.5 L/min. The interface voltage was (+), 4.5 kV; (−), −3.5 kV, and the detector voltage was 1.70 kV. The relative collision-induced dissociation energy was 50%.

A “5-point screening” method [3] was used to rapidly pick out the precursors of interest according to the principle of mass defect filter using the five points (x, y) of estimated lowest *m/z* (441, 0.3783), estimated highest mass defection (503, 0.3378), estimated lowest *m/z* with the lowest mass defection (591, 0.4630), estimated highest *m/z* with the highest mass defection (1421, 0.5505), and estimated highest *m/z* with the lowest mass defection (1467, 0.8150), in which x represented the integral part of *m/z*, and y represents the decimal part of *m/z* (Figure S20 in Supplemental Materials). Most potential precursors of saponins that were screened by “5-point screening” method were further analyzed manually. Only a small number of potential precursors, which could not be screened by the “5-point screening” method were directly analyzed manually.

### 4.6. “Quantitative Analysis of Multi-Components with Single Marker” (QAMS) Method

According to our previous research on the QAMS method for quantitative analysis of eleven saponins in notoginseng [22], the contents of seven ginsenosides, including ginsenoside Re, Rf, Rb_1_, Rg_2_, Rh_1_, and Rd, were assayed referred to ginsenoside Rg_1_ using relative correction factors, with the chromatographic peaks of the seven ginsenosides identified referring to chromatograms of reference substances (Figure S21 in Supplemental Materials) and HPLC-IT-TOF-MS^n^. The concentration of Rg_1_ in sample solution was calculated with standard curve Y = 4311.6X + 2.2 established with reference substance according to the method established in our previous publication [22], where Y represented the chromatographic peak area and X represented the concentration of Rg_1_. The concentrations of the other six ginsenosides were calculated with the formulations referring to correction factors established in our previous study [22], which were X = Y/3312.2 for Rb_1_, X = Y/3899.1 for Re, X = Y/3675.7 for Rd, X = Y/5034.8 for Rf, X = Y/4751.5 for Rg_2_, and X = Y/5333.6 for Rh_1_.

### 4.7. Data Analysis

The similarities of chromatographic fingerprints among W-GS, F-GS, and C-GS were analyzed via Similarity Evaluation System for Chromatographic Fingerprint of TCM (Chinese Pharmacopoeia Commission, version 2012), following with the correlation analysis between fingerprint similarities of all ginseng samples and all peak areas within the retention time of 5–70 min via R (version 4.0.2) with rcorr function in hmisc package. The correlation between contents of ginsenosides and growing ages was also analyzed via R (version 4.0.2) with rcorr function in hmisc package. All plots (including box plot and scattered plot) and *t*-tests were run by R (version 4.0.2), as well.

## 5. Conclusions

In the present study, 177 saponins were tentatively identified from two wild ginseng samples via HPLC-IT-TOF-MS^n^ with 162 saponins detected from wild ginseng for the first time. Additionally, 56 and 60 saponins were tentatively identified from ginseng under forest and cultivated ginseng, respectively, which were far less than the number detected in wild ginseng. Sixteen potential new compounds were tentatively identified from wild ginseng, ginseng under forest, cultivated ginseng, with five potential new aglycones only found in wild ginseng and ginseng under forest, and four potential new aglycones only found in cultivated ginseng. Saponin compositions were found to be accumulated along with the prolongation of growing age. Additionally, 23 of the 50 saponins only detected from both the two wild ginseng samples might be characteristic markers for wild ginseng (ginseng older than 50 years) to distinguish from ginseng younger than 25 years. The contents of Rg_1_, Re, Rf, Rb_1_, Rg_2_, and the total contents of the seven ginsenosides were significantly related to the growing ages of ginseng (*p* < 0.10). Additionally, all types of saponins (including glucosides, rhamnosides, and xylosides) tentatively identified showed the highest accumulation in wild ginseng. The contents of ginsenosides Re, Rh_1_, or Rd in wild ginseng were remarkably higher than those in ginseng under forest and cultivated ginseng, which could be used as markers for distinguishing from ginseng under forest or cultivated ginseng. Using the contents of Re, Rf or Rb_1_, approximately a half number of cultivated ginseng samples could be identified from ginseng under forest when those contents were remarkably below the corresponding contents lower limits of ginseng under forest. Additionally, the chromatographic fingerprints of ginseng under forest were more similar to wild ginseng comparing to cultivated ginseng. A characteristic peak pattern composed of five chromatographic peaks was discovered for distinguishing wild ginseng, ginseng under forest, and cultivated ginseng as well.

Collectively, wild ginseng was remarkably superior to ginseng under forest and cultivated ginseng in saponin compositions and contents. Ginseng under forest was slightly closer to wild ginseng in ginsenosides’ contents and chromatographic fingerprint comparing to cultivated ginseng. The differences among wild ginseng, ginseng under forest, and cultivated ginseng were mainly attributed to their growing ages.

## Figures and Tables

**Table 1:** Summary of saponins tentatively identified from wild ginseng (W-GS), ginseng under forest (F-GS), and cultivated ginseng (C-GS) via HPLC-IT-TOF-MS^n^.

Number of Saponins	W-GS (nos. 20 and 19)	F-GS (no. 18)	C-GS (no. 25)	Total ^1^
Total identified in each ginseng type	177 (161 in no. 20; 112 in no. 19)	56	60	199
First time found in each ginseng type	162	5	9	184
First time found in ginseng ^2^	54 (45 in no. 20; 41 in no.19)	5	9	62
Potential new compounds	11 (7 in no. 20; 10 in no. 19)	1	6	16
Rhamnosides identified	41 (39 in no. 20; 29 in no. 19)	8	11	48
Xylosides identified	41 (39 in no. 20; 27 in no. 19)	17	16	42

^1^ Total numbers were not the summation of numbers from the four samples, because there were saponins detected from more than one sample; ^2^ including potential new compounds. W-GS no. 20 was approximately 100 years old (estimated); W-GS no. 19 was approximately 50 years old (estimated); F-GS no. 18 was 25 years old; C-GS no. 25 was 6 years old (Table 8).

**Table 3:** Individual contents of seven ginsenosides in three types of ginseng samples assayed by “quantitative analysis of multi-component with single marker” (QAMS) method referring to ginsenoside Rg_1_ (mg/g).

Sample no.	Rg_1_	Re	Rf	Rb_1_	Rg_2_	Rh_1_	Rd	Total
Wild Ginseng (W-GS, *n* = 2)								
19	7.43	13.91	1.03	6.12	1.64	7.46	12.94	50.52
20	6.58	12.36	1.61	12.34	1.99	7.65	12.98	55.51
Ginseng Under Forest (F-GS, *n* = 27)								
1	1.00	1.78	0.29	1.88	0.32	0.84	0.52	6.63
2	1.49	1.85	0.39	1.95	1.36	0.73	0.66	8.44
3	1.58	1.22	0.39	2.11	0.10	0.93	0.96	7.29
4	5.20	2.41	1.03	5.24	2.67	1.10	0.64	18.30
5	4.20	2.91	0.90	5.30	2.71	0.82	1.04	17.88
6	2.46	2.30	0.53	3.29	0.27	1.43	0.75	11.04
7	4.24	2.45	0.78	4.41	2.26	0.83	0.80	15.77
8	2.57	2.43	0.83	6.08	2.95	1.45	0.84	17.16
9	3.50	2.57	0.75	4.39	2.37	1.49	0.90	15.97
10	3.08	3.42	0.87	4.36	2.46	1.26	0.77	16.22
11	2.14	2.42	0.55	3.71	0.29	1.16	0.76	11.02
12	1.26	1.62	0.34	1.72	0.88	0.28	0.19	6.31
13	2.09	2.43	0.51	3.37	1.22	0.64	0.31	10.57
14	1.01	1.49	0.37	1.24	0.65	0.25	0.68	5.69
15	1.84	2.71	0.49	2.52	1.31	0.83	0.67	10.36
16	2.09	2.69	0.49	3.57	1.89	0.55	0.52	11.80
17	2.99	1.90	0.57	2.53	1.85	0.51	0.56	10.89
18	4.48	2.92	0.79	5.39	3.36	1.35	0.45	18.74
27	2.16	2.22	0.57	4.64	1.22	0.20	1.00	12.01
28	2.56	3.26	0.66	3.80	1.30	0.26	0.92	12.76
29	1.61	2.78	0.44	3.33	0.29	1.19	0.67	10.31
30	3.01	2.28	0.76	6.99	0.28	1.22	0.90	15.44
31	2.91	2.26	0.77	3.25	0.95	0.28	0.91	11.32
32	4.59	2.54	0.79	7.57	1.23	0.38	0.77	17.86
33	6.22	2.22	1.19	4.51	0.62	0.37	0.70	15.83
34	2.49	2.18	0.81	2.03	0.95	0.15	0.71	9.31
35	4.11	3.50	0.74	5.39	2.02	0.51	1.49	17.76
Cultivated Ginseng (C-GS, *n* = 11)								
21	0.47	0.96	0.22	0.57	0.11	0.25	0.41	2.99
22	0.56	0.43	0.16	0.45	2.32	0.19	0.36	4.46
23	0.84	0.67	0.23	0.76	0.09	0.16	0.40	3.14
24	0.55	1.13	0.25	0.78	0.13	0.26	0.56	3.66
25	4.23	3.45	0.95	6.13	0.51	3.42	0.86	19.55
26	2.68	2.91	0.51	3.38	2.02	1.73	1.68	14.92
36	2.82	1.89	0.63	4.36	3.96	0.83	1.12	15.62
37	2.90	1.98	0.54	5.11	4.21	0.89	1.16	16.79
38	0.33	0.30	0.14	0.89	0.14	1.23	0.48	3.50
39	2.68	1.47	0.69	2.58	0.78	2.08	0.52	10.80
40	1.41	0.96	0.28	1.23	0.11	0.05	0.17	4.22

**Table 4:** Mean, median and range of seven ginsenosides’ contents (mg/g) in three types of ginseng samples.

Summary of Content	Rg_1_	Re	Rf	Rb_1_	Rg_2_	Rh_1_	Rd	Total
Wild Ginseng (W-GS, *n* = 2)								
Range	6.58–7.43	12.36–13.91	1.03–1.61	6.12–12.34	1.64–1.99	7.46–7.65	12.94–12.98	50.52–55.51
Ginseng Under Forest (F-GS, *n* = 27)								
Range	1.00–6.22	1.22–3.50	0.29–1.19	1.24–7.57	0.10–3.36	0.15–1.49	0.19–1.49	5.69–18.74
Mean ± SD	2.85 ± 1.33	2.40 ± 0.55	0.65 ± 0.22	3.87 ± 1.63	1.40 ± 0.94	0.78 ± 0.43	0.74 ± 0.25	14.21 ± 10.85
Median	2.56	2.42	0.66	3.71	1.23	0.82	0.75	11.80
Cultivated Ginseng (C-GS, *n* = 11)								
Range	0.33–4.23	0.30–3.45	0.14–0.95	0.45–6.13	0.09–4.21	0.05–3.42	0.17–1.68	2.99–19.55
Mean ± SD	1.77 ± 1.34	1.47 ± 1.01	0.42 ± 0.26	2.39 ± 2.06	1.31 ± 1.58	1.01 ± 1.05	0.70 ± 0.45	9.06 ± 6.53
Median	1.41	1.13	0.28	1.23	0.51	0.83	0.52	4.46

**Table 5:** Mean, median and range of seven ginsenosides’ contents (mg/g) in ginseng (including ginseng under forest and cultivated ginseng) with different growing ages.

Summary of Content	Rg_1_	Re	Rf	Rb_1_	Rg_2_	Rh_1_	Rd	Total
Ginseng Under Forest ≥ 15 years old (*n* = 10)								
Range	1.84–4.48	1.90–3.50	0.49–0.87	2.03–5.39	0.27–3.36	0.15–1.49	0.45–1.49	9.31–18.74
Mean ± SD	3.13 ± 0.93	2.66 ± 0.51	0.68 ± 0.14	3.79 ± 1.19	1.87 ± 0.86	0.89 ± 0.47	0.76 ± 0.29	13.79 ± 3.44
Median	3.04	2.63	0.74	3.96	1.96	0.83	0.73	13.78
Ginseng Under Forest and Cultivated Ginseng < 15 years old (*n* = 24)								
Range	0.47–6.22	0.43–3.45	0.16–1.19	0.45–7.57	0.09–2.95	0.05–3.42	0.17–1.68	2.99–19.55
Mean ± SD	2.34 ± 1.56	2.03 ± 0.82	0.56 ± 0.29	3.33 ± 2.12	1.01 ± 0.91	0.76 ± 0.74	0.70 ± 0.33	10.74 ± 5.43
Median	2.12	2.24	0.51	3.35	0.76	0.51	0.69	10.80
Ginseng Under Forest and Cultivated Ginseng between 10 to 15 years old (*n* = 13)								
Range	1.00–5.10	1.22–3.50	0.29–1.03	1.24–7.57	0.10–2.95	0.15–1.45	0.52–1.49	5.69–18.30
Mean ± SD	2.86 ± 1.37	2.33 ± 0.67	0.69 ± 0.24	4.10 ± 2.15	1.43 ± 1.01	0.76 ± 0.42	0.83 ± 0.24	13.02 ± 4.96
Median	2.91	2.28	0.77	4.36	1.23	0.82	0.77	15.44
Ginseng Under Forest ≥ 10 years old (*n* = 20)								
Range	1.00–5.20	1.22–3.50	0.29–1.03	1.24–7.57	0.10–3.36	0.15–1.49	0.45–1.49	5.69–18.74
Mean ± SD	2.94 ± 1.24	2.39 ± 0.57	0.67 ± 0.21	3.97 ± 1.82	1.60 ± 1.00	0.84 ± 0.43	0.78 ± 0.23	13.19 ± 4.36
Median	2.95	2.42	0.76	3.96	1.60	0.83	0.76	13.62
Ginseng Under Forest and Cultivated Ginseng < 10 years old (*n* = 14)								
Range	0.47–6.22	0.43–3.45	0.16–1.19	0.45–6.13	0.09–2.32	0.05–3.42	0.17–1.68	2.99–19.55
Mean ± SD	2.06 ± 1.58	1.96 ± 0.99	0.49 ± 0.29	2.74 ± 1.81	0.79 ± 0.73	0.72 ± 0.92	0.64 ± 0.40	9.41 ± 5.34
Median	1.85	2.22	0.48	3.35	0.56	0.27	0.62	10.44

Plots for comparisons between ginseng samples with ages below and above 6 years, 10 years, and 15 years were provided in Figures S22–S25 in Supplemental Materials.

**Table 6:** Correlations between contents of the seven ginsenosides and the growing ages for ginseng samples with certain growing ages (*n* = 34).

Contents—Ages	Rg_1_	Re	Rf	Rb_1_	Rg_2_	Rh_1_	Rd	Total
Correlation Coefficient	0.33	0.37	0.30	0.29	0.45	0.15	0.09	0.38
*p*-Value	0.0550	0.0297	0.0795	0.0986	0.0082	0.4108	0.6130	0.0247

**Table 7:** Chromatographic peaks recognized by correlation analysis between peak areas and fingerprint similarities referring to wild ginseng samples.

	cRT (min) ^1^	Correlation Coefficient (*p*-Value)		Identification
		Wild Ginseng no. 19	Wild Ginseng no. 20	
1	15.682	0.4572 (0.0030)	0.4510 (0.0046)	(B4-b)-glc-xyl ^2^
2	16.576	0.4096 (0.0092)	0.4389 (0.00461)	(B3-b)-glc-rha ^2^
3	17.491	0.3398 (0.0319)	NC ^4^	Ginsenoside Re_4_ or its isomer ^2^
4	32.061	0.6414 (<0.0001)	06259 (0.00002)	Ginsenoside Rg_1_^3^
5	34.474	0.6805 (<0.0001)	0.5963 (0.00005)	Ginsenoside Re ^3^
6	47.367	0.6188 (<0.0001)	0.6438 (<0.0001)	Ginsenoside Rf ^3^
7	47.630	0.5426 (0.0003)	0.4938 (0.0012)	Notoginsenoside Fc ^2^
8	48.778	0.6295 (<0.0001)	0.7030 (<0.0001)	Ginsenoside Rb_1_^3^
9	50.122	0.6753 (<0.0001)	0.6209 (<0.0001)	Ginsenoside F_3_^2^

^1^ Corrected retention time of the reference fingerprint automatically generated by similarity evaluation system; ^2^ tentatively identified via HPLC-IT-TOF-MS^n^; ^3^ identified with reference substances; ^4^ NC: no significant correlation (*p* > 0.05).

## Data Availability

Data is contained within the Supplemental Materials.

## Supplementary Materials

The following are available online. Figures S1–S19, MS^n^ ESI(−) fragmentation of ginsenosides Rg_1_, Re, Rf, Rb_1_, Rd, and 14 potential new compounds; Table S1, Detailed HPLC-MS information of 199 saponins tentatively identified from wild ginseng (nos. 19 and 20), ginseng under forest (no. 18) and cultivated ginseng (no. 25); Table S2, Saponins detected from “all the four ginseng samples” and “both the two wild ginseng samples” by HPLC-IT-TOF-MS^n^; Figure S20, Potential saponin precursors extracted by “5-point screening” method; Figure S21, Overlapped chromatograms of reference substances of ginsenosides Rg_1_, Re, Rf, Rb_1_, Rg_2_, Rh_1_, and Rd; Figures S21–25, Comparisons of contents of the seven ginsenosides; Figure S26, Chromatographic fingerprints obtained by HPLC-DAD of 40 ginseng samples with retention time correction by 10 common peaks using Similarity Evaluation System for Chromatographic Fingerprint of TCM version 2012; Table S3, Peak areas (manually integrated) of the five peaks within the characteristic peak pattern and the ratio of signal to noise.

## Sample Availability

Samples of the compounds are not available from the authors.
