# Supplementary material for: 177 Saponins, Including 11 New Compounds in Wild Ginseng Tentatively Identified via HPLC-IT-TOF-MSn, and Differences among Wild Ginseng, Ginseng under Forest, and Cultivated Ginseng
Source: Molecules. 2021 Jun 2;26(11):3371. doi: 10.3390/molecules26113371 (PMC8199768; doi:10.3390/molecules26113371)
Supplement: Supplementary file 1 [file molecules-26-03371-s001.zip › molecules-1214501-supplementary.pdf]

molecules

Supplementary Materials

177 Saponins, Including 11 New Compounds in Wild Ginseng Tentatively Identified via HPLC-IT-TOF-MS^n, and Differences among Wild Ginseng, Ginseng under Forest, and Cultivated Ginseng

Chao-Qun Wang, Li-Wei Yi, Lin Zhao, Yu-Zhen Zhou, Fang Guo, Yu-Shu Huo, Da-Qing Zhao, Feng Xu *, Xuan Wang * and Shao-Qing Cai *

Contents

Figure S1. MS^n ESI(-) fragmentation of ginsenoside Rg_1 (retention time in F-GS sample No. 18: 28.105 min).
Figure S2. MS^n ESI(-) fragmentation of ginsenoside Re (retention time in W-GS sample No. 20: 35.347 min).
Figure S3. MS^n ESI(-) fragmentation of ginsenoside Rf (retention time in W-GS sample No. 46.813 min).
Figure S4. MS^n ESI(-) fragmentation of ginsenoside Rb_1 (retention time in W-GS sample No. 19: 48.265 min).
Figure S5. MS^n ESI(-) fragmentation of ginsenoside Rd (retention time in W-GS sample No. 19: 54.120 min).
Figure S6. MS^n ESI(-) fragmentation of the potential new compound ($C_{52}H_{88}O_{23}$), and the locations and orders of sugar residues and substituent group (-$C_4H_6O_4$) might be different (retention time in W-GS sample No. 19: 3.938 min).
Figure S7. MS^n ESI(-) fragmentation of the potential new compound ($C_{51}H_{80}O_{21}$) with unknown aglycone predicted as $C_{33}H_{50}O_7$, and the locations and orders of sugar residues might be different (retention time in C-GS sample No. 25: 4.147 min).
Figure S8. MS^n ESI(-) fragmentation of the potential new compound ($C_{48}H_{80}O_{20}$) with possible aglycone of C1-b (shown in the figure), C3-b and B10, and the locations and orders of sugar residues might be different (retention time in W-GS sample No. 20: 6.350 min).
Figure S9. MS^n ESI(-) fragmentation of the potential new compound ($C_{45}H_{68}O_{16}$) with unknown aglycone predicted as $C_{33}H_{48}O_7$, and the locations and orders of sugar residues might be different (retention time in C-GS sample No. 25: 6.970 min).
Figure S10. MS^n ESI(-) fragmentation of the potential new compound ($C_{45}H_{70}O_{17}$) with unknown aglycone predicted as $C_{30}H_{44}O_7$ (retention time in C-GS sample No. 25: 9.168 min).
Figure S11. MS^n ESI(-) fragmentation of the potential new compound ($C_{54}H_{84}O_{26}$) with unknown aglycone predicted as $C_{30}H_{44}O_6$ (retention time in C-GS sample No. 25: 11.687 min).
Figure S12. MS^n ESI(-) fragmentation of the potential new compound ($C_{48}H_{80}O_{18}$) with aglycone predicted as PPD (retention time in W-GS sample No. 19: 19.317 min).
Figure S13. MS^n ESI(-) fragmentation of the potential new compound ($C_{36}H_{64}O_{10}$) with predicted aglycone of B1-b,

and the location of sugar residue might be different (retention time in W-GS sample No. 20: 20.045 min).

Figure S14. MS^n ESI(-) fragmentation of the potential new compound ($C_{43}H_{72}O_{16}$) with unknown aglycone predicted as $C_{31}H_{52}O_7$ (retention time in W-GS sample No. 20: 20.270 min).

Figure S15. MS^n ESI(-) fragmentation of the potential new compound ($C_{38}H_{66}O_{13}$) with unknown aglycone predicted as $C_{26}H_{46}O_4$ (retention time in W-GS sample No. 19: 26.555 min).

Figure S16. MS^n ESI(-) fragmentation of the potential new compound ($C_{41}H_{68}O_{13}$) with predicted aglycone of B8-b, and the locations of sugar residues might be different (retention time in W-GS sample No. 20: 40.932 min).

Figure S17. MS^n ESI(-) fragmentation of the potential new compound ($C_{54}H_{92}O_{24}$) with predicted aglycone of B4-a, and the locations and orders of sugar residues might be different (retention time in W-GS sample No.20: 41.082 min).

Figure S18. MS^n ESI(-) fragmentation of the potential new compound ($C_{39}H_{68}O_{14}$) with unknown aglycone predicted as $C_{27}H_{48}O_4$ (retention time in W-GS sample No. 20: 44.095 min).

Figure S19. MS^n ESI(-) fragmentation of the potential new compound ($C_{38}H_{64}O_{14}$) with unknown aglycone predicted as $C_{26}H_{44}O_5$ (retention time in W-GS sample No. 19: 45.002 min).**Table S1.** Detailed LC-MS information of 199 compounds tentatively identified from wild ginseng (No. 19 and No. 20), ginseng under forest (No. 18) and cultivated ginseng (No. 25)

Table S1. Detailed LC-MS information of 199 saponins tentatively identified from wild ginseng (nos. 19 and 20), ginseng under forest (no. 18) and cultivated ginseng (no. 25)

Table S2. Saponins detected from "all the four ginseng samples" and "both the two wild ginseng samples" by HPLC-IT-TOF-MS^n

Figure S20. Potential saponin precursors (red spots) extracted by "5-point screening" method with the 5 points (x, y) of estimated lowest *m*/*z* (441, 0.3783), estimated highest mass defection (503, 0.3378), estimated lowest *m*/*z* with the lowest mass defection (591, 0.4630), estimated highest *m*/*z* with the highest mass defection (1421, 0.5505), and estimated highest *m*/*z* with the lowest mass defection (1467, 0.8150), in which x represented the integral part of *m*/*z*, and y represents the decimal part of *m*/*z*.

Figure S21. Overlapped chromatograms of reference substances of ginsenosides Rg_1, Re, Rf, Rb_1, Rg_2, Rh_1, and Rd.

Figure S22. Comparison of the contents of seven ginsenosides and their total content between ginseng under forest with ages older than 6 years (n = 26), and ginseng under forest, cultivated ginseng with age of 6 years (n = 10; * p<0.05; ns, no significance).

Figure S23. Comparison of the contents of seven ginsenosides and their total content between ginseng under forest with ages older than 10 years (n = 20), and ginseng under forest, cultivated ginseng with age younger than 10 years (n = 16; ns, no significance).

Figure S24. Comparison of the contents of seven ginsenosides and their total content between ginseng under forest with ages older than 15 years (n = 10), and ginseng under forest, cultivated ginseng with age younger than 15 years (n = 26; ** p<0.01; ns, no significance).

Figure S25. Comparison of the contents of seven ginsenosides and their total content between ginseng under forest with ages older than 15 years (n = 7), between 10 to 15 years (n = 13) and ginseng under forest, cultivated ginseng with age younger than 10 years (n = 14; * p<0.01; ns, no significance).

Figure S26. Chromatographic fingerprints obtained by HPLC-DAD of 40 ginseng samples with retention time correction by 10 common peaks using Similarity Evaluation System for Chromatographic Fingerprint of TCM version 2012 (S1–S40: ginseng samples No. 1–40; R: reference fingerprint automatically generated by software)

Table S3. Peak areas (manually integrated) of the five peaks within the characteristic peak pattern and the ratio of signal-to-noise

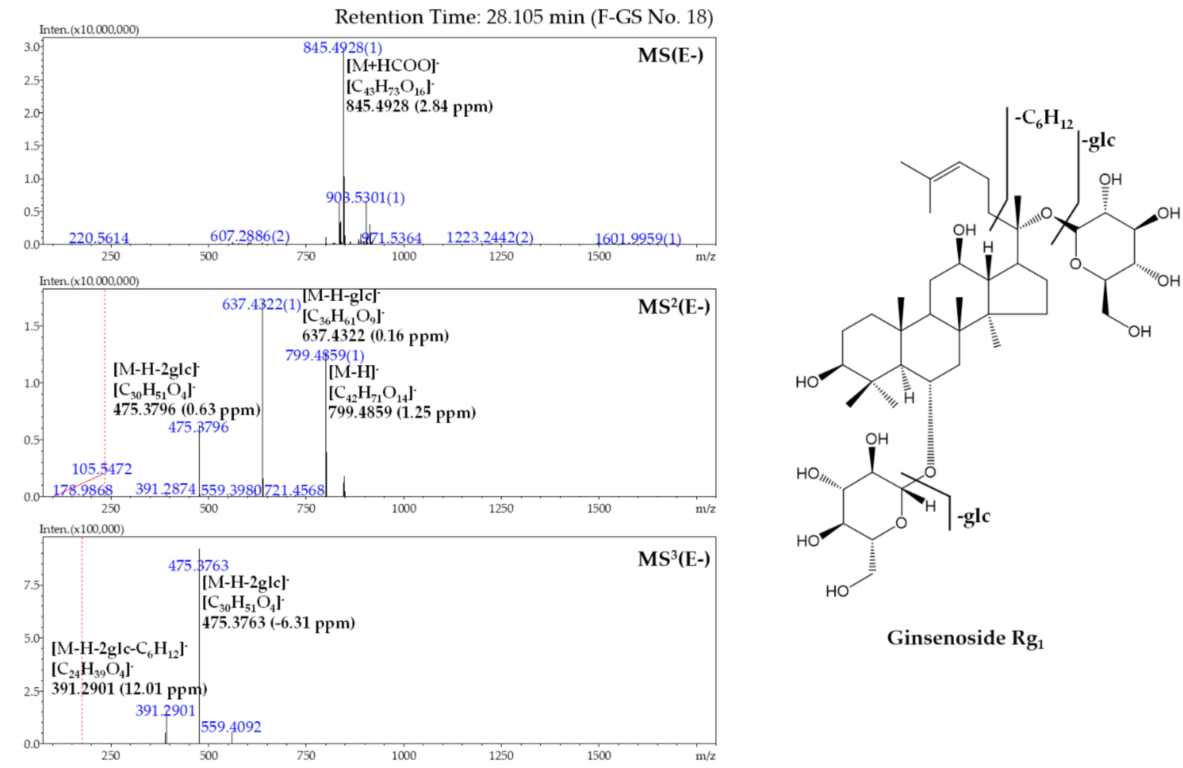

Figure S1. MS^n ESI(-) fragmentation of ginsenoside Rg$_1$ (retention time in F-GS sample No. 18: 28.105 min).

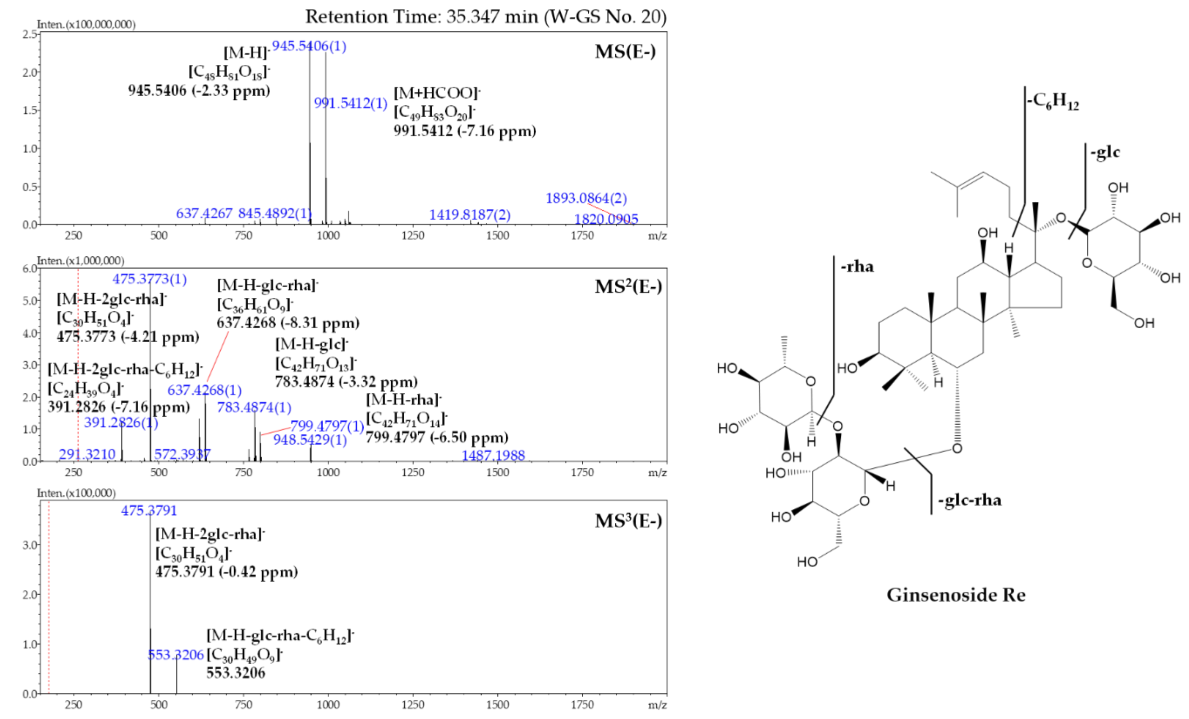

Figure S2. MS^n ESI(-) fragmentation of ginsenoside Re (retention time in W-GS sample No. 20: 35.347 min).

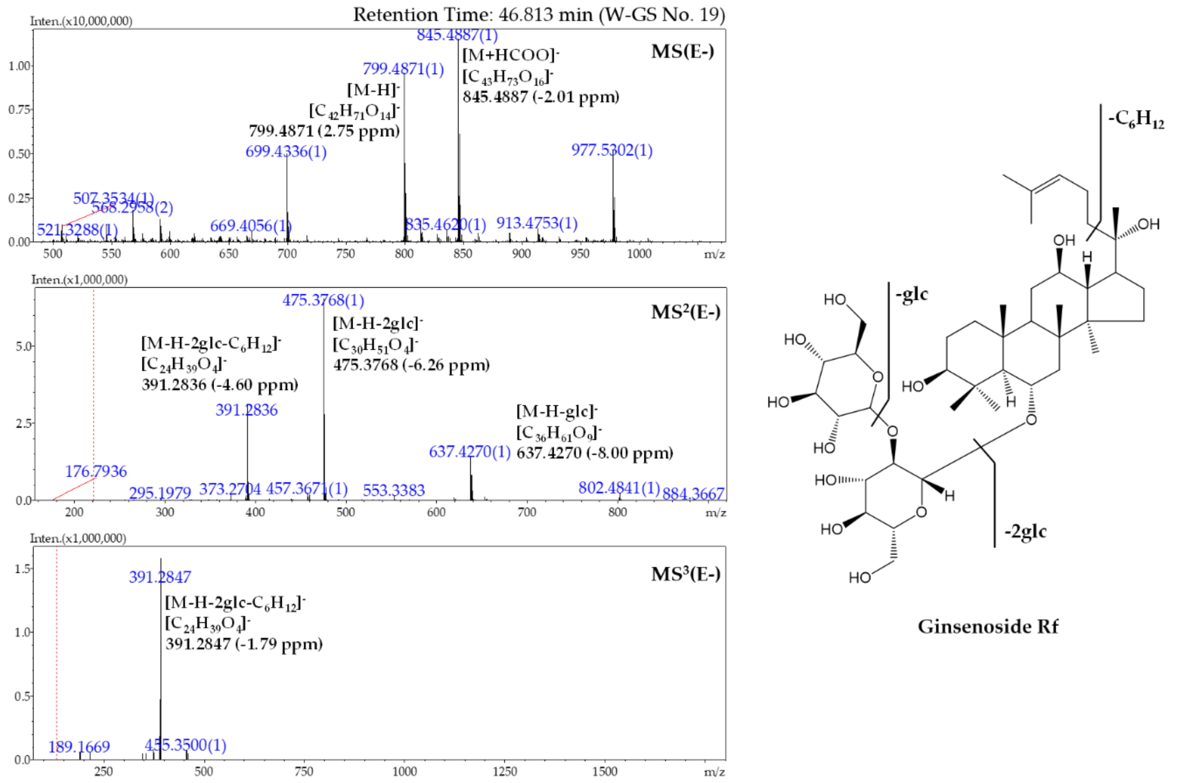

Figure S3. MS^n ESI(-) fragmentation of ginsenoside Rf (retention time in W-GS sample No. 46.813 min).

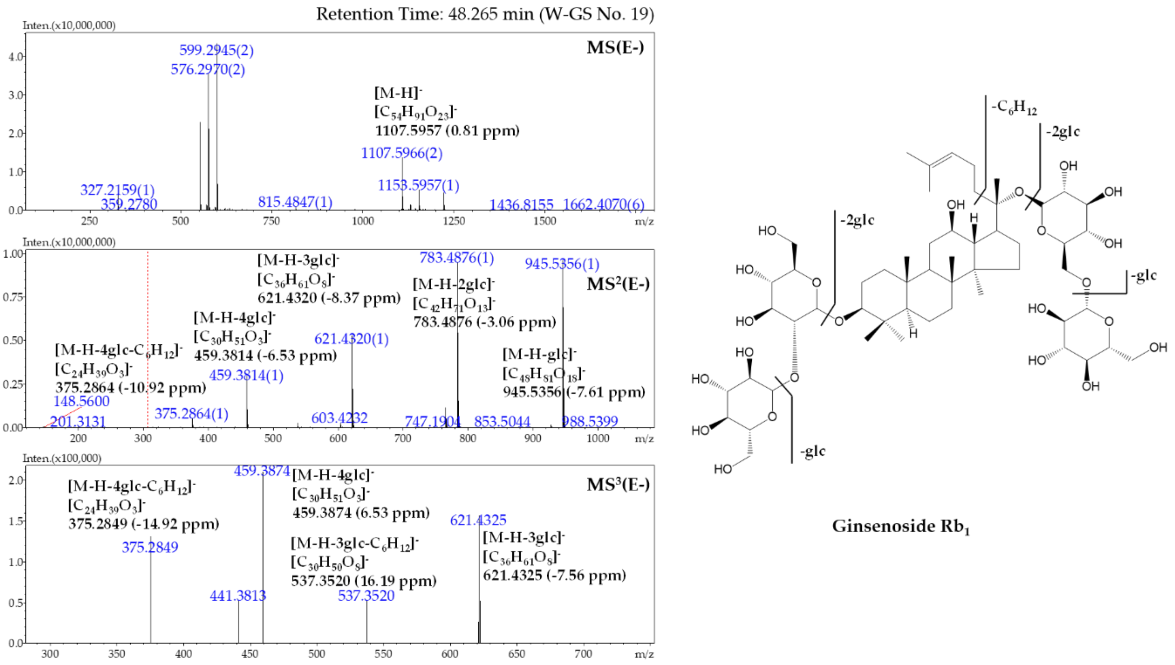

Figure S4. MS^n ESI(-) fragmentation of ginsenoside Rb1 (retention time in W-GS sample No. 19: 48.265 min).

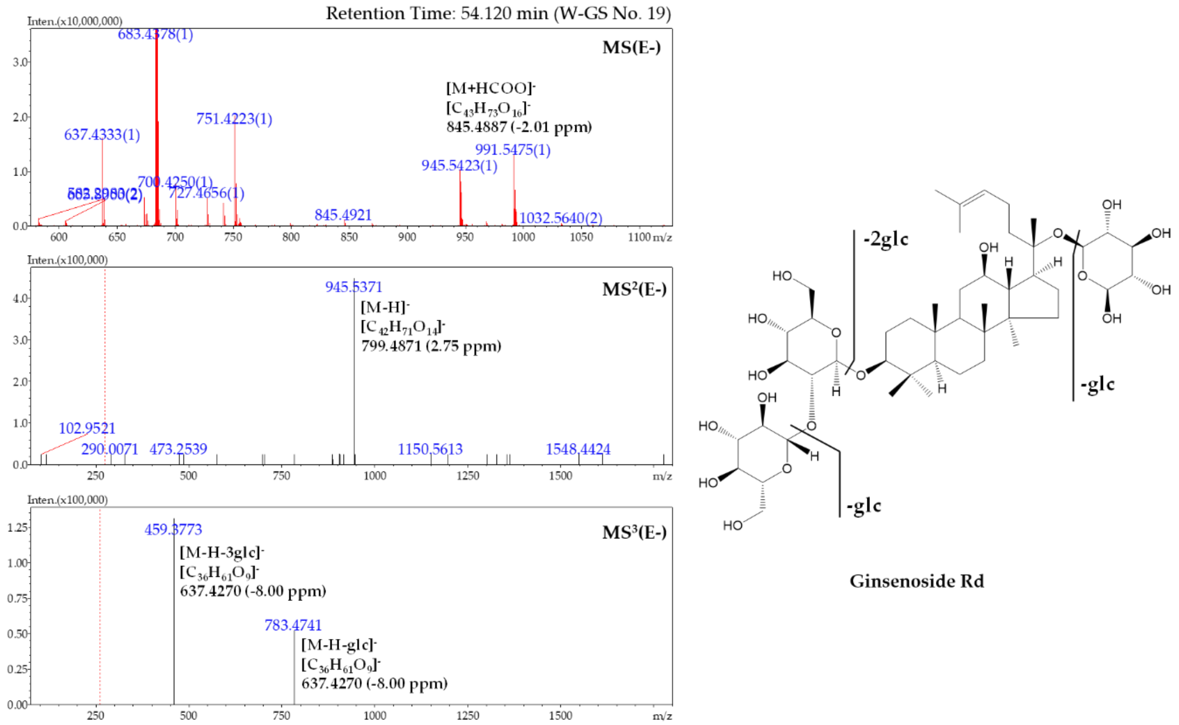

Figure S5. MS^n ESI(-) fragmentation of ginsenoside Rd (retention time in W-GS sample No. 19: 54.120 min).

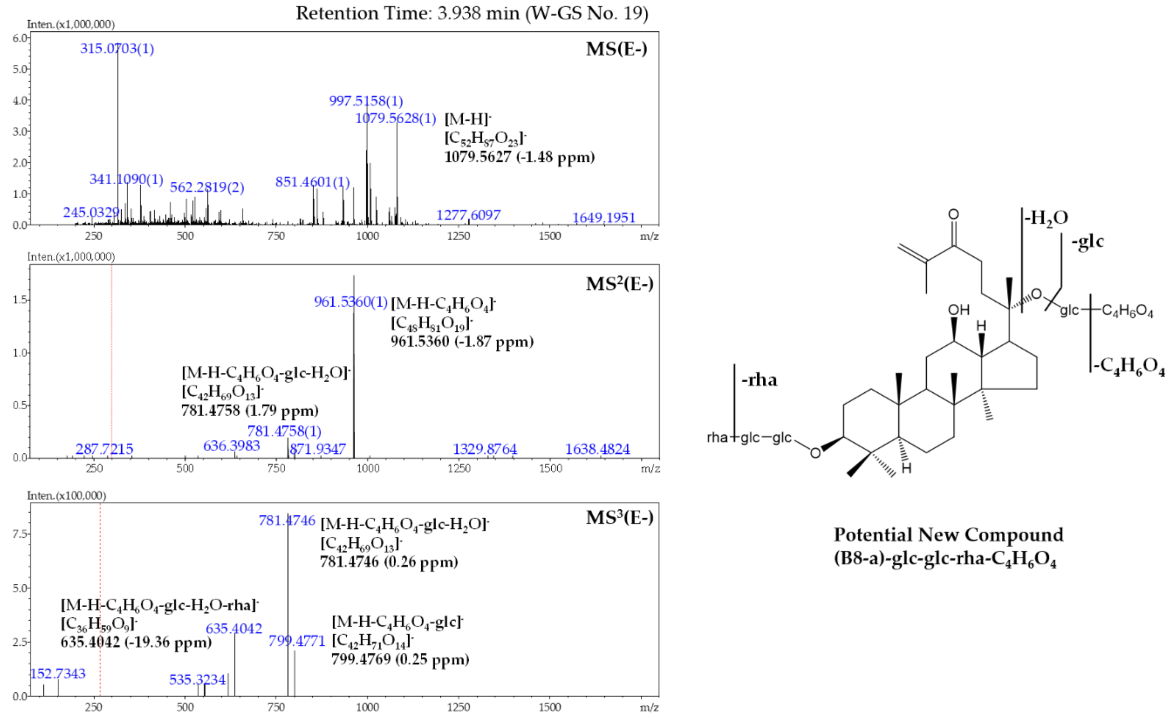

Figure S6. MS^n ESI(-) fragmentation of the potential new compound ($C_{52}H_{88}O_{23}$), and the locations and orders of sugar residues and substituent group ($-C_4H_6O_4$) might be different (retention time in W-GS sample No. 19: 3.938 min).

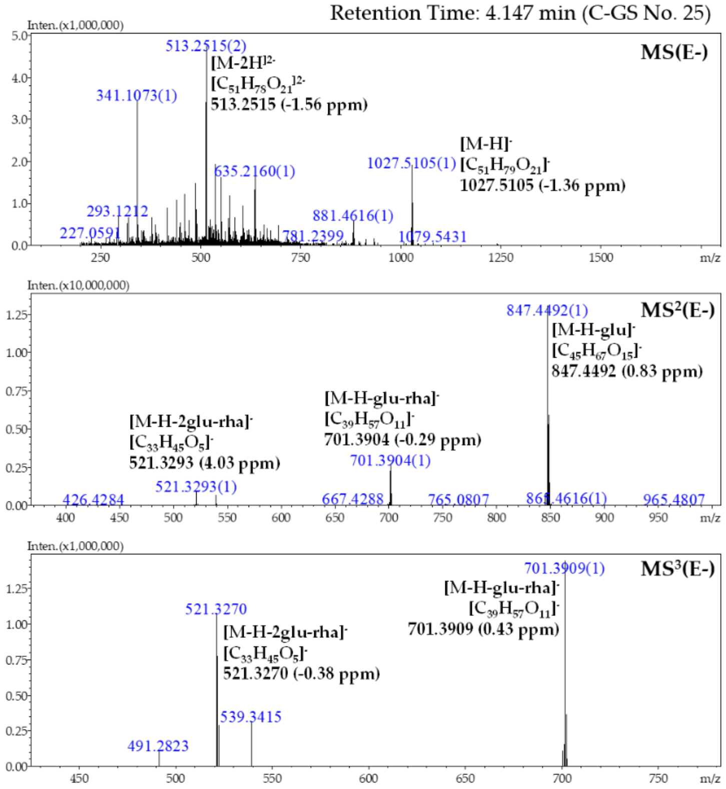

Figure S7. MS^n ESI(-) fragmentation of the potential new compound ($C_{51}H_{80}O_{21}$) with unknown aglycone predicted as $C_{33}H_{50}O_7$, and the locations and orders of sugar residues might be different (retention time in C-GS sample No. 25: 4.147 min).

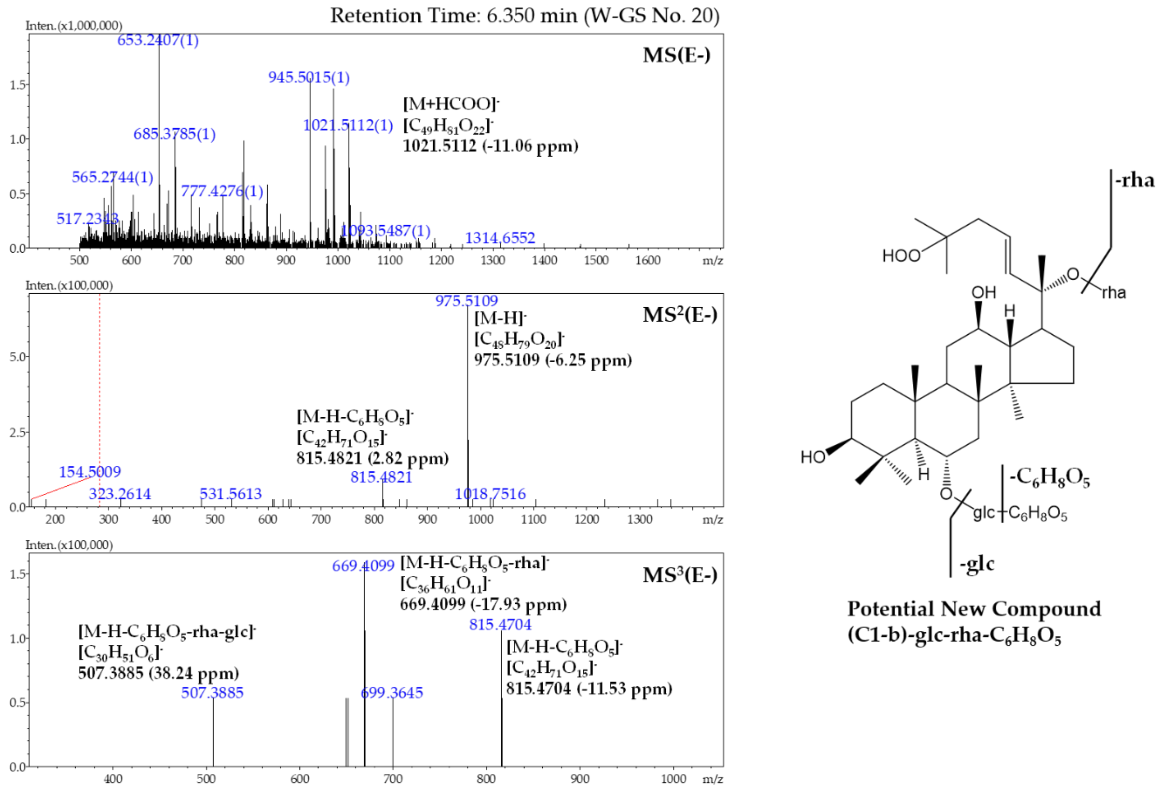

Figure S8. MS[n] ESI(-) fragmentation of the potential new compound ($C_{48}H_{80}O_{20}$) with possible aglycone of C1-b (shown in the figure), C3-b and B10, and the locations and orders of sugar residues might be different (retention time in W-GS sample No. 20: 6.350 min).

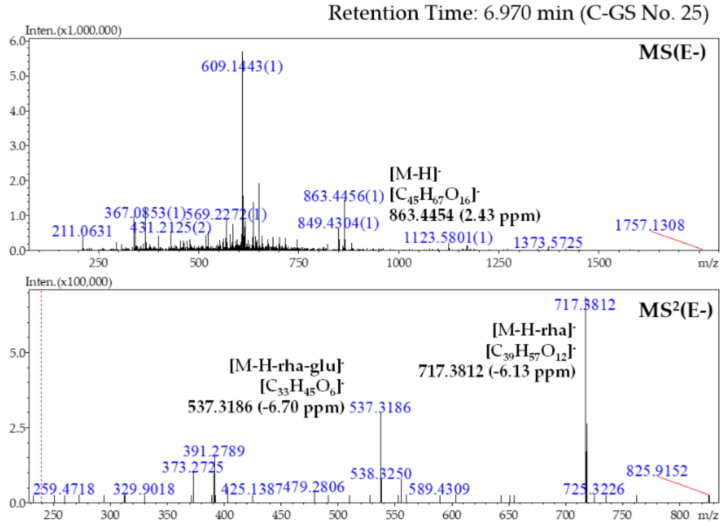

Figure S9. MS[n] ESI(-) fragmentation of the potential new compound ($C_{45}H_{68}O_{16}$) with unknown aglycone predicted as $C_{33}H_{48}O_7$, and the locations and orders of sugar residues might be different (retention time in C-GS sample No. 25: 6.970 min).

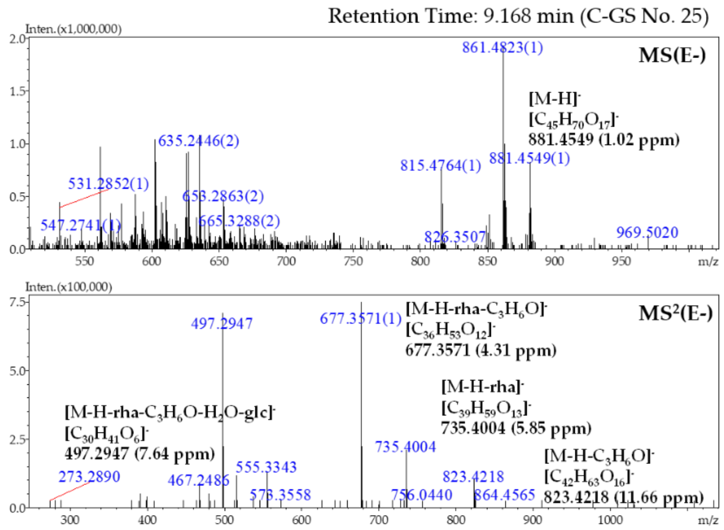

Figure S10. MSn ESI(-) fragmentation of the potential new compound ($C_{45}H_{70}O_{17}$) with unknown aglycone predicted as $C_{30}H_{44}O_7$ (retention time in C-GS sample No. 25: 9.168 min).

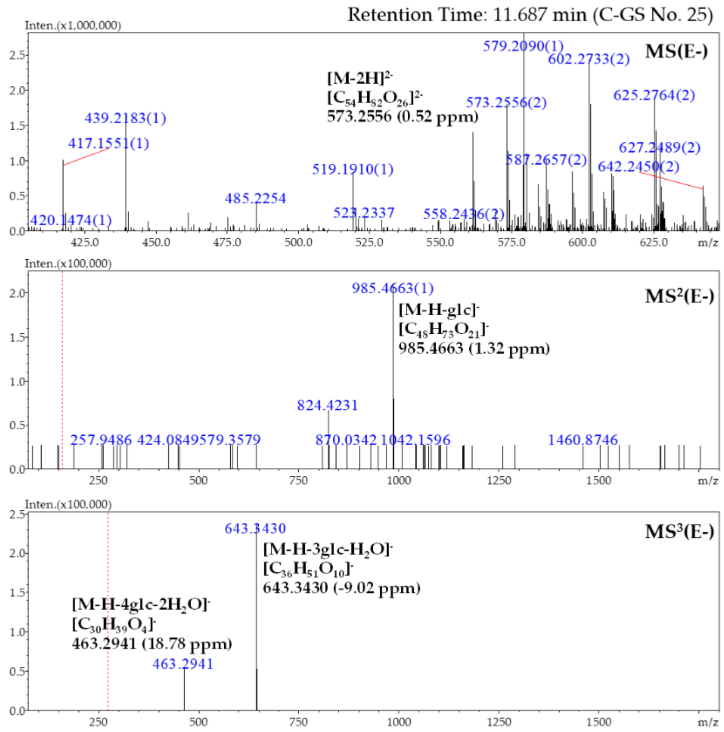

Figure S11. MSn ESI(-) fragmentation of the potential new compound ($C_{54}H_{84}O_{26}$) with unknown aglycone predicted as $C_{30}H_{44}O_6$ (retention time in C-GS sample No. 25: 11.687 min).

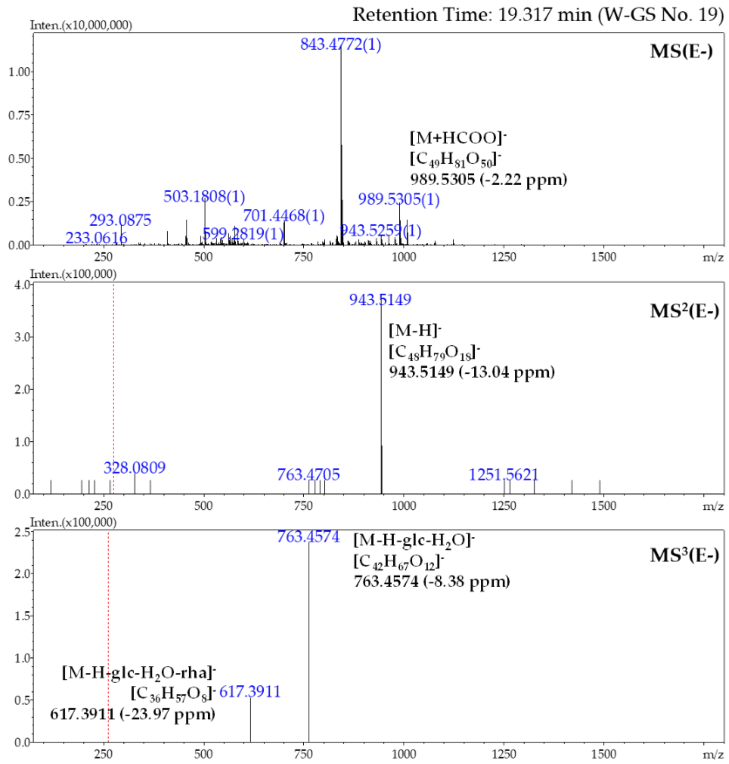

Figure S12. MS^n ESI(-) fragmentation of the potential new compound ($C_{48}H_{80}O_{18}$) with aglycone predicted as PPD (retention time in W-GS sample No. 19: 19.317 min).

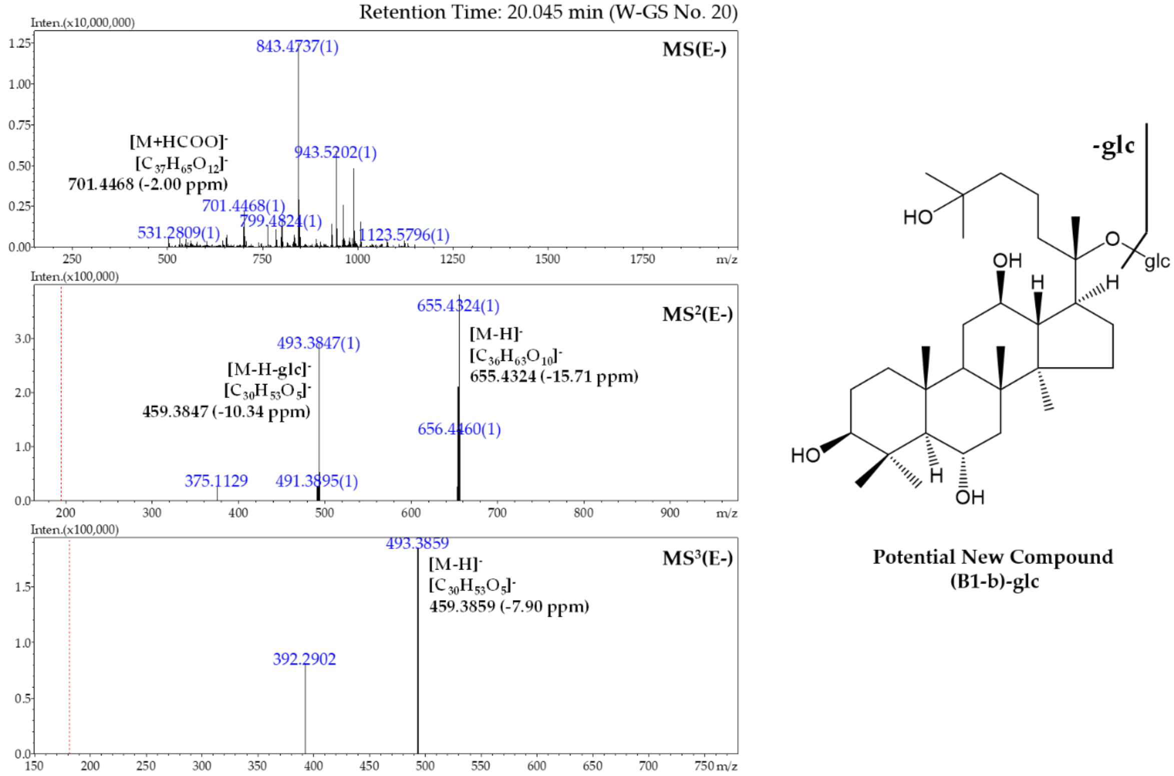

Figure S13. MS^n ESI(-) fragmentation of the potential new compound ($C_{36}H_{64}O_{10}$) with predicted aglycone of B1-b, and the location of sugar residue might be different (retention time in W-GS sample No. 20: 20.045 min).

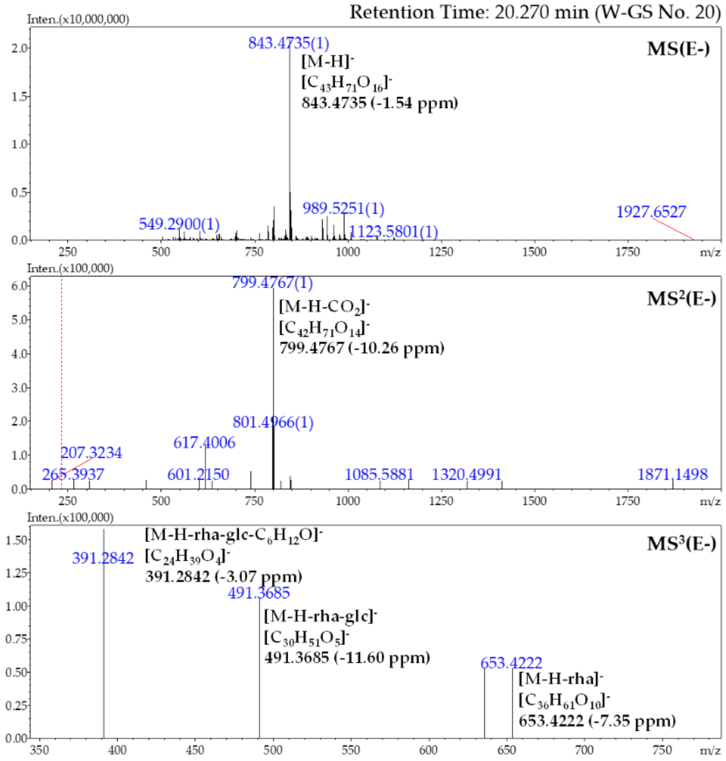

Figure S14. MS^n ESI(-) fragmentation of the potential new compound ($C_{43}H_{72}O_{16}$) with unknown aglycone predicted as $C_{31}H_{52}O_7$ (retention time in W-GS sample No. 20: 20.270 min).

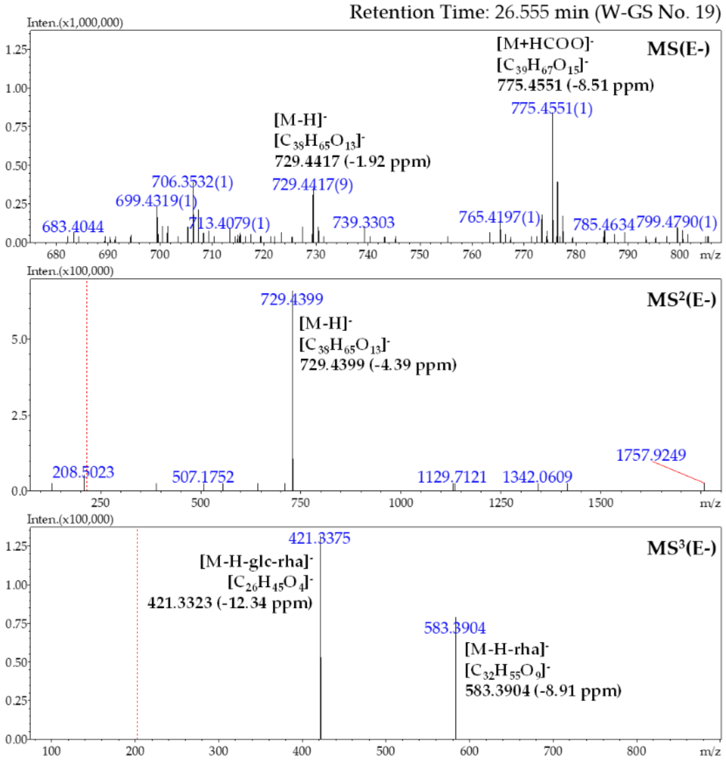

Figure S15. MS^n ESI(-) fragmentation of the potential new compound ($C_{38}H_{66}O_{13}$) with unknown aglycone predicted as $C_{26}H_{46}O_4$ (retention time in W-GS sample No. 19: 26.555 min).

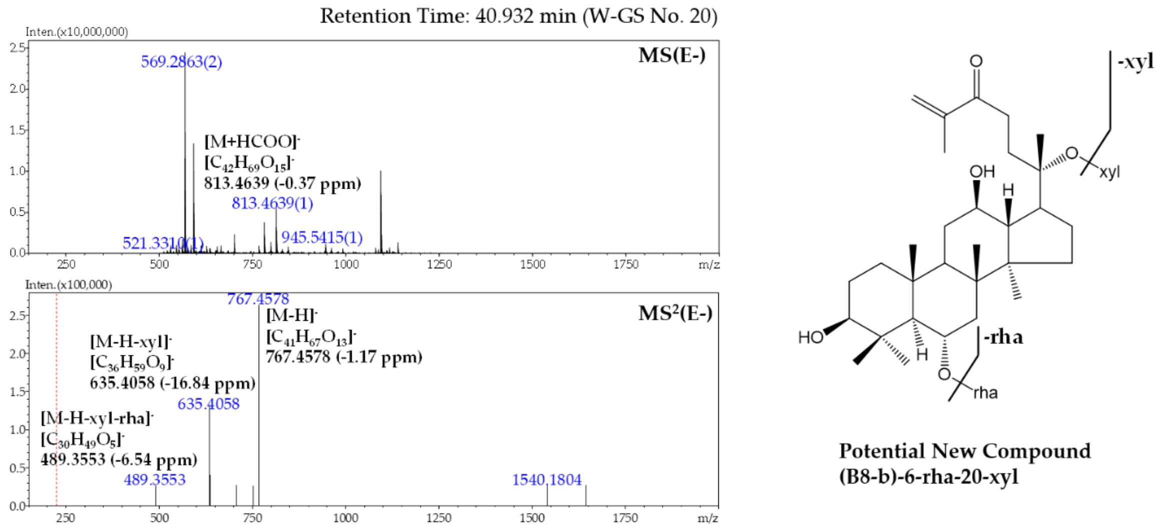

Figure S16. MSn ESI(-) fragmentation of the potential new compound ($C_{41}H_{68}O_{13}$) with predicted aglycone of B8-b, and the locations of sugar residues might be different (retention time in W-GS sample No. 20: 40.932 min).

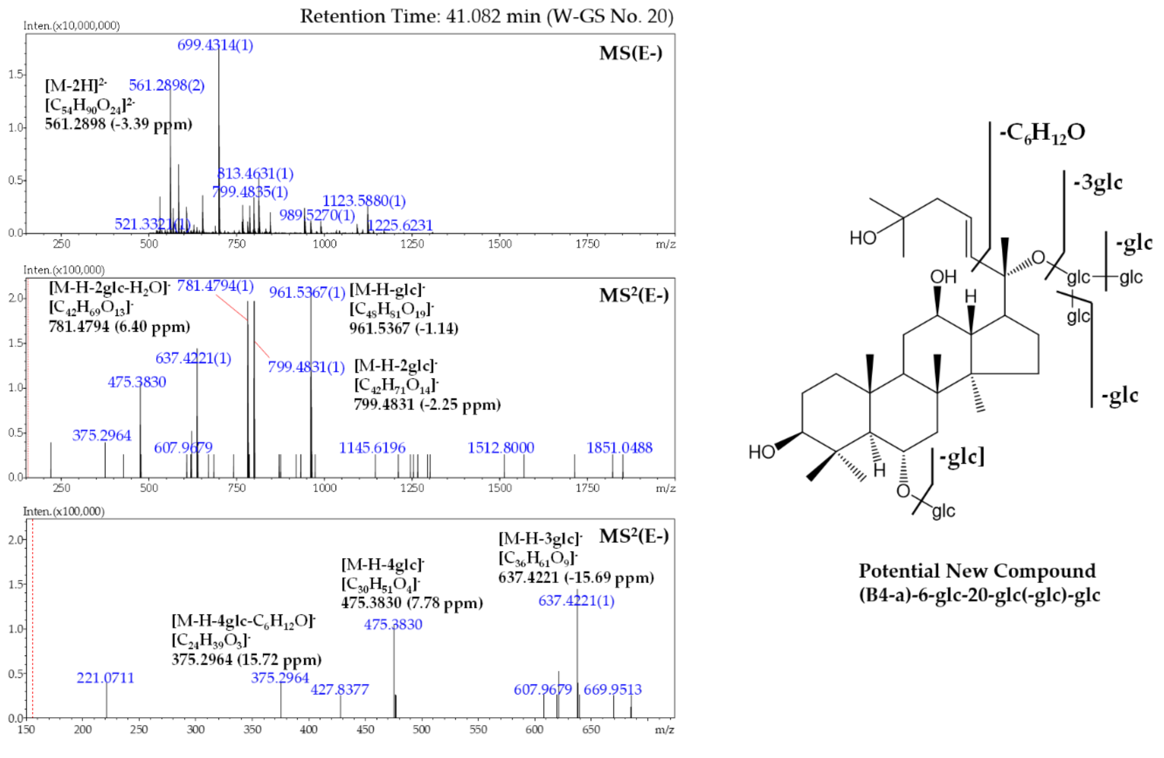

Figure S17. MSn ESI(-) fragmentation of the potential new compound ($C_{54}H_{92}O_{24}$) with predicted aglycone of B4-a, and the locations and orders of sugar residues might be different (retention time in W-GS sample No.20: 41.082 min).

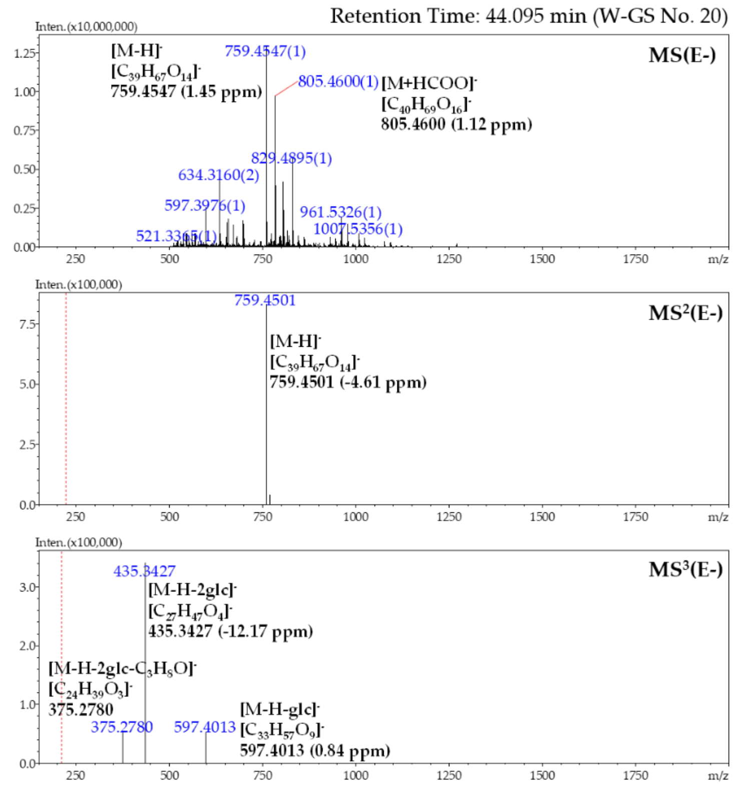

Figure S18. MS^n ESI(-) fragmentation of the potential new compound ($C_{39}H_{68}O_{14}$) with unknown aglycone predicted as $C_{27}H_{48}O_4$ (retention time in W-GS sample No. 20: 44.095 min).

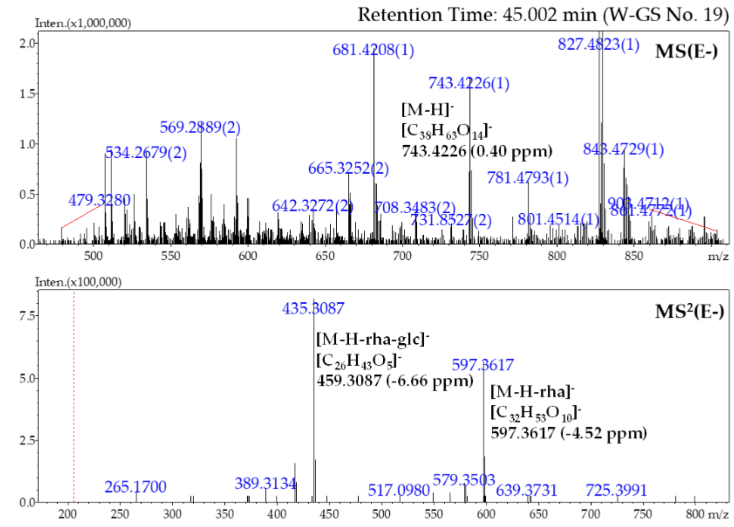

Figure S19. MS^n ESI(-) fragmentation of the potential new compound ($C_{38}H_{64}O_{14}$) with unknown aglycone predicted as $C_{26}H_{44}O_5$ (retention time in W-GS sample No. 19: 45.002 min).

Table S1 Detailed LC-MS information of 199 saponins tentatively identified from wild ginseng (nos. 19 and 20), ginseng under forest (no. 18) and cultivated ginseng (no. 25)

no.	Retention Time	Molecular Formula	Measured (*m/z*)	Predicted (*m/z*)	Diff (ppm)	Ion	ESI-MS^n Data	Identification	Sample	Ref
1	3.828[d]	$C_{51}H_{78}O_{21}$	1025.4949	1025.4693	-1.37	$[M-H]^-$	MS^2[1025.4944]: 845.4316(100), 699.3736(10.89), 537.3200(3.27)<br>MS^3[1025.4944→845.4311]: 699.3765(100), 521.3255(30.33)	unknown-glc-glc-rha[#]	no. 25	[1]
2**	3.938[b]	$C_{52}H_{88}O_{23}$	1079.5628	1079.5644	-1.48	$[M-H]^-$	MS^2[1079.5627]: 962.5320(100), 961.5308(76.47), 781.4758(11.26)<br>MS^3[1079.5627→961.5361]: 799.4771(4.42), 781.4756(100), 635.4042(23.49), 617.4012(5.15)	(B8-a)-glc-glc-rha-$C_4H_6O_4$<br>(Structure not found in SciFinder)	no. 19	New
3**	4.147[d]	$C_{51}H_{80}O_{21}$	1027.5105	1027.5119	-1.36	$[M-H]^-$	MS^2[1027.5104]: 847.4492(100)<br>MS^3[1027.5104→847.4492]: 701.3909(100), 521.3270(57.60), 491.2823(5.26)	Unknown($C_{33}H_{50}O_7$)-glc-glc-rha<br>(Formula not found in SciFinder)	no. 25	New
4*	4.202[b]	$C_{48}H_{82}O_{19}$	1007.5439	1007.5432	0.69	$[M+HCOO]^-$	MS^2[1007.5436]: 961.5367(100)<br>MS^3[1007.5436→961.5366]: 781.4740(100), 492.3685(100)	(B4-b)/B6-6-glc(-glc)-rha<br>(First found in ginseng)	no. 19	[1][Q]
5*	5.342[a]	$C_{48}H_{82}O_{20}$	977.5274	977.5327	-5.42	$[M-H]^-$	MS^2[977.5274]: 815.4743(10.94), 797.4658(100), 779.4522(31.93)<br>MS^3[977.5274→797.4655]: 489.3343(100)	(C3-b)-6-glc-20-glc-rha<br>(First found in ginseng)	no. 20<br>no. 19	[1][Q]
6*	5.953[a]	$C_{36}H_{64}O_{11}$	717.4382	717.4431	-6.83	$[M+HCOO]^-$	MS^2[717.4382]: 671.4384(100)<br>MS^3[717.4382→671.4385]: 509.3826(100), 391.0719(7.87)	(B2-b)-20-glc<br>(First found in ginseng)	no. 20<br>no. 19	[1][Q]
7*	6.005[a]	$C_{42}H_{74}O_{15}$	817.4922	817.4955	-4.04	$[M-H]^-$	MS^2[817.4919]: 671.4344(72.87), 653.4237(41.80), 509.3836(100)<br>MS^3[817.4919→509.3837]: 390.2670(100)	Quinquenoside L_9 or its isomer<br>(First found in ginseng)	no. 20	[1][N,Q]
8**	6.350[a]	$C_{48}H_{80}O_{20}$	1021.5112	1021.5225	-11.06	$[M+HCOO]^-$	MS^2[1021.5113]: 975.5109(100), 815.4821(11.98)<br>MS^3[1021.5113→975.5104]: 815.4704(47.05), 669.4099(100)<br>MS^4[1021.5113→975.5104→669.4099]: 507.3565(100)	(C1-b)/(C3-b)/(B10)-6-glc-$C_6H_8O_5$-20-rha<br>(Structure not found in SciFinder)	no. 20<br>no. 19	New
9**	6.970[d]	$C_{45}H_{68}O_{16}$	863.4456	863.4435	2.43	$[M-H]^-$	MS^2[863.4454]: 717.3812(100), 537.3186(31.79), 391.2789(14.69)	Unknown($C_{33}H_{48}O_7$)-glc-rha<br>(Structure not found in SciFinder)	no. 25	New
10*	7.487[b]	$C_{36}H_{64}O_{11}$	717.4433	717.4431	0.28	$[M+HCOO]^-$	MS^2[717.4435]: 671.4343(100)<br>MS^3[717.4435→671.4343]: 509.3846(100)	(B2-b)-glc[#]<br>(First found in ginseng)	no. 19	[1][Q]
11*	7.675[a]	$C_{48}H_{82}O_{19}$	961.5301	961.5378	-8.01	$[M-H]^-$	MS^2[961.5298]: 799.4790(57.41), 781.4657(81.63), 653.4219(100), 635.4123(89.70), 491.3708(40.63), 415.3250(8.70)	(C1-a)/C2/(C3-a)-3-glc-20-glc-rha<br>(First found in ginseng)	no. 20	[1][Q]
12*	7.770[a]	$C_{41}H_{72}O_{15}$	849.4806	849.4853	-5.53	$[M+HCOO]^-$	MS^2[849.4808]: 803.4776(100)<br>MS^3[849.4808→803.4774]: 509.3744(100)	(B2-b)-glc-xyl[#]<br>(First found in ginseng)	no. 20<br>no. 19	[1][Q]
13	7.903[a]	$C_{48}H_{80}O_{19}$	1005.5202	1005.5276	-7.36	$[M+HCOO]^-$	MS^2[1005.5198]: 959.5156(100)<br>MS^3[1005.5198→959.5151]: 779.4500(60.19), 471.3480(100)	(B8-b)-6-glc-rha-20-glc	no. 20	[1]
14**	7.965[d]	$C_{35}H_{44}O_{10}$	623.2878	623.2862	2.57	$[M+HCOO]^-$	MS^2[623.2879]: 577.2841(100)<br>MS^3[623.2879→577.2842]: 415.2188(100)	Unknown-glc	no. 20<br>no. 25	New
15	7.975[a]	$C_{42}H_{74}O_{15}$	863.4975	863.5010	-4.05	$[M+HCOO]^-$	MS^2[863.4973]: 817.4894(100)<br>MS^3[863.4973→817.4896]: 671.4397(66.07), 653.4237(29.55), 509.3843(100)<br>MS^4[863.4973→817.4896→509.3845]: 391.3000(100)	Quinquenoside L_9 or its isomer	no. 20<br>no. 19	[1]
16*	8.347[b]	$C_{36}H_{62}O_{11}$	715.4279	715.4274	0.70	$[M+HCOO]^-$	MS^2[715.4279]: 669.4187(100), 507.3682(84.53)	Ginsenoside SL_1 or its isomer	no. 19<br>no. 20	[2][S]
17*	8.468[c]	$C_{30}H_{54}O_5$	493.2291	-	-	$[M-H]^-$	MS^2[493.2291]: 407.1580(100), 331.1223(4.55), 263.0733(53.51), 221.0679(14.43), 179.0554(13.30)	(B1-b)/(B2-a)	no. 18<br>no. 19	[1]

no.	Retention Time	Molecular Formula	Measured (*m/z*)	Predicted (*m/z*)	Diff (ppm)	Ion	ESI-MS^n Data	Identification	Sample	Ref
									no. 20<br>no. 25	
18*	8.875[b]	$C_{30}H_{52}O_5$	491.2136	-	-	$[M-H]^-$	MS^2[491.2136]: 405.1436(100)	Isomer of hydroxyl PPT<br>(First found in ginseng)	no. 19	
19**	9.168[d]	$C_{45}H_{70}O_{17}$	881.4549	881.4540	1.02	$[M-H]^-$	MS^2[881.4550]: 823.4218(17.73), 735.4004(35.88), 677.3571(97.45), 555.3343(15.43), 515.3014(8.70), 497.2947(100)	Unknown($C_{30}H_{44}O_7$)-glc-rha-C_3H_6O<br>(Not found in SciFinder)	no. 25	New
20	9.183[b]	$C_{42}H_{72}O_{15}$	815.4788	815.4798	-1.23	$[M-H]^-$	MS^2[815.4790]: 653.4222(5.07), 553.3394(64.75), 491.3685(3.04), 391.2864(100)	B6/(B4-b)-glc-glc[#]	no. 19<br>no. 18<br>no. 25	[1]
21*	9.273[b]	$C_{36}H_{64}O_{11}$	717.4444	717.4431	1.81	$[M+HCOO]^-$	MS^2[717.4447]: 671.4352(100)<br>MS^3[717.4447→671.4354]: 509.3957(100)	Vinaginsenoside R_{12} or its isomer<br>(First found in ginseng)	no. 19<br>no. 20	[1][Q,]
22	9.335[a]	$C_{48}H_{82}O_{20}$	1023.5291	1023.5381	-8.79	$[M+HCOO]^-$	MS^2[1023.5289]: 977.5180(100)<br>MS^3[1023.5289→977.5179]: 491.3475(100)	B6/(B3-b)/(B4-b)-glc-glc-glc[#]	no. 20<br>no. 19	[1]
23**	9.447[b]	$C_{35}H_{40}O_7$	617.2671	617.2756	-13.77	$[M+HCOO]^-$	MS^2[617.2656]: 571.2617(100)<br>MS^3[617.2656→571.2617]: 439.1954(100), 277.1775(100)	Unknown($C_{30}H_{32}O_3$)-xyl<br>(Similar structure not found in SciFinder)	no. 19<br>no. 18	New
24	9.725[a]	$C_{42}H_{74}O_{15}$	863.4933	863.5010	-8.92	$[M+HCOO]^-$	MS^2[863.4932]: 817.4901(100)<br>MS^3[863.4932→816.4893]: 493.3853(100), 417.3250(8.59)	(C1-a)-glc-glc[#]	no. 20	[1]
25	10.165[c]	$C_{48}H_{82}O_{19}$	961.5394	961.5378	1.66	$[M-H]^-$	MS^2[961.5393]: 799.4859(20.96), 637.4331(69.96), 475.3793(100), 391.2783(6.76)	PPT-6-glc-glc-glc	no. 18	[1]
26	10.548[b]	$C_{42}H_{72}O_{15}$	861.4835	861.4853	-2.09	$[M+HCOO]^-$	MS^2[861.4831]: 815.4795(100)<br>MS^3[861.4831→815.4796]: 491.3663(100), 403.3212(13.27)	Ginsenoside Re_5 or its isomer	no. 18<br>no. 19<br>no. 20<br>no. 25	[1]
27*	10.965[a]	$C_{41}H_{72}O_{15}$	849.4805	849.4853	-5.65	$[M+HCOO]^-$	MS^2[849.4805]: 803.4790(100)<br>MS^3[849.4805→803.4790]: 509.3637(100), 293.0794(100)	(B2-b)-glc-xyl[#]<br>(First found in ginseng)	no. 20<br>no. 19	[1]
28**	11.687[d]	$C_{54}H_{84}O_{26}$	573.2556	573.2553	0.52	$[M-2H]^{2-}$	MS^2[573.2557(2)]: 985.4663(100)<br>MS^3[573.2557(2)→985.4663]: 643.3430(100), 463.2941(23.54)	Unknown($C_{30}H_{44}O_6$)-glc-glc-glc-glc<br>(Not found in SciFinder)	no. 25	New
29	11.828[a]	$C_{48}H_{82}O_{20}$	977.5263	977.5327	-6.55	$[M-H]^-$	MS^2[977.5259]: 815.4760(100), 797.4652(68.20), 653.4182(98.94), 491.3715(37.42), 403.3275(4.21)<br>MS^3[977.5259→815.4762]: 653.4205(16.70), 491.3712(100)<br>MS^4[977.5259→815.4762→653.4208]: 491.3790(100)	(B4-b)/B6-6-glc-20-glc-glc	no. 20	[1]
30	11.928[a]	$C_{54}H_{92}O_{23}$	1107.5863	1107.5957	-8.49	$[M-H]^-$	MS^2[1107.5868]: 945.5319(100), 783.4859(27.26), 765.4632(15.28), 637.4230(18.33), 475.3764(51.70)	PPT-6-glc(-glc)-rha-20-glc	no. 20	[1]
31	12.102[a]	$C_{42}H_{72}O_{15}$	861.4809	861.4853	-5.11	$[M+HCOO]^-$	MS^2[861.4807]: 815.4721(100)<br>MS^3[861.4807→816.4758]: 653.4464(56.98), 491.3775(100), 391.2779(85.47)<br>MS^4[861.4807→816.4758→653.4466]: 491.3685(100)	B6-6-glc-glc	no. 20<br>no. 19<br>no. 25	[1]
32	12.153[a]	$C_{42}H_{72}O_{14}$	845.4826	845.4904	-9.23	$[M+HCOO]^-$	MS^2[845.4824]: 799.4701(77.31), 637.4203(100)<br>MS^3[845.4824→637.4202]: 475.3866	PPT-20-glc-glc	no. 20<br>no. 18	[1]

no.	Retention Time	Molecular Formula	Measured (*m/z*)	Predicted (*m/z*)	Diff (ppm)	Ion	ESI-MS^n Data	Identification	Sample	Ref
33	12.637 [a]	$C_{53}H_{90}O_{23}$	1093.5715	1093.5800	-7.77	$[M-H]^-$	MS^2[1093.5709]: 961.5190(63.16), 931.5140(26.59), 799.4861(46.43), 637.4299(93.26), 475.3763(100)	PPT-6-glc-20-glc-(glc)-xyl	no. 20	[1]
34	12.660 [a]	$C_{52}H_{88}O_{22}$	1063.5652	1063.5694	-3.95	$[M-H]^-$	MS^2[1063.5652]: 931.4915(12.10), 799.4861(9.71), 637.4290(100), 475.3783(53.52)	PPT-6-glc-20-glc-(xyl)-xyl	no. 20	[1]
35*	13.443 [a]	$C_{36}H_{64}O_{11}$	717.4407	717.4431	-3.35	$[M+HCOO]^-$	MS^2[717.4410]: 671.4346(100), 509.3822(27.61)<br>MS^3[717.4410→671.4348]: 510.3794(100)<br>MS^4[717.4410→671.4348→510.3795]: 391.2779(100), 347.1508(100)	(B2-b)-glc#<br>(First found in ginseng)	no. 20<br>no. 19	[1]Q
36	13.832 [a]	$C_{48}H_{82}O_{19}$	961.5335	961.5378	-4.47	$[M-H]^-$	MS^2[961.5333]: 799.4800(54.30), 781.4852(31.32), 637.4369(28.01), 619.4211(100), 475.3777(93.45), 391.2904(9.93)	PPT-6-glc-glc-glc	no. 20<br>no. 25	[1]
37	14.317 [a]	$C_{41}H_{72}O_{14}$	787.4843	787.4849	-0.76	$[M-H]^-$	MS^2[787.4846]: 655.4427(14.49), 493.3866(100)<br>MS^3[787.4846→655.4429]: 493.3859(100)	(B2-a)/(B1-b)-glc-xyl<br>(First found in ginseng)	no. 20	
38	14.500 [a]	$C_{47}H_{78}O_{19}$	945.4996	945.5065	-7.30	$[M-H]^-$	MS^2[945.4994]: 765.4423(100), 603.3894(32.11), 441.3332(6.82)	Unknown($C_{29}H_{48}O_4$)-glc-glc-glc	no. 20<br>no. 19	
39	14.743 [c]	$C_{41}H_{70}O_{14}$	831.4758	831.4748	1.20	$[M+HCOO]^-$	MS^2[831.4760]: 785.4689(100)<br>MS^3[831.4760→785.4688]: 653.4302(62.93), 491.3745(84.73), 391.2857(100)	(B4-b)/B6-glc-xyl#	no. 18<br>no. 19	[1]
40	15.098 [d]	$C_{45}H_{70}O_{16}$	865.4600	865.4591	1.04	$[M-H]^-$	MS^2[865.4600]: 719.3996(93.02), 539.3384(100), 509.2889(5.33)	Unknown($C_{33}H_{50}O_7$)-glc-rha<br>(Structure not found in SciFinder)	no. 25	New
41*	15.560 [a]	$C_{36}H_{62}O_{10}$	699.4306	699.4325	-2.72	$[M+HCOO]^-$	MS^2[699.4308]: 653.4255(50.22), 553.3346(100), 491.3771(9.41), 391.2819(39.15)	B6/(B3-b)-glc#<br>(First found in ginseng)	no. 20<br>no. 19	[1]N
42	15.617 [a]	$C_{41}H_{70}O_{14}$	831.4698	831.4748	-6.01	$[M+HCOO]^-$	MS^2[831.4698]: 785.4672(100)<br>MS^3[831.4698→785.4670]: 653.4170(13.21), 491.3703(100)<br>MS^4[831.4698→785.4670→653.4171]: 491.3709(100)	(B4-b)-glc-xyl#	no. 20	[1]
43	15.880 [a]	$C_{54}H_{92}O_{23}$	1107.5895	1107.5957	-5.60	$[M-H]^-$	MS^2[1107.5896]: 945.5340(100), 637.4306(26.19), 619.4085(9.83), 475.3783(22.82)<br>MS^3[1107.5896→945.5336]: 799.4593(100), 475.3783(100)	Ginsenoside Re_8 or its isomer	no. 20<br>no. 19	[1]
44	16.285 [a]	$C_{42}H_{72}O_{15}$	861.4788	861.4853	-7.55	$[M+HCOO]^-$	MS^2[861.4785]:815.4720(35.20), 653.4178(100), 635.4057(38.06), 553.3378(77.63), 391.2779(11.22)<br>MS^3[861.4785→653.4181]: 553.3296(100), 391.2967(79.69)	(B3-b)/(B4-b)-6-glc-glc	no. 20<br>no. 19	[1]
45	16.305 [b]	$C_{36}H_{62}O_{10}$	699.4341	699.4325	2.29	$[M+HCOO]^-$	MS^2[699.4343]: 653.4219(100), 491.3742(35.38)<br>MS^3[699.4343→653.4220]: 491.3750(100), 403.3195(12.16), 391.2779(2.90)	(B4-b)/B4-glc	no. 20<br>no. 19	[1]
46	16.558 [c]	$C_{48}H_{82}O_{19}$	961.5321	961.5378	4.47	$[M-H]^-$	MS^2[961.5423]: 799.4776(29.77), 637.4300(68.66), 619.4210(6.15), 475.3791(100), 391.2808(20.04)<br>MS^3[961.5423→475.3791]: 391.2880(100)	20-glc-ginsenoside Rf or its isomer	no. 18	[1]
47	16.832 [a]	$C_{42}H_{72}O_{14}$	799.4806	799.4849	-5.38	$[M-H]^-$	MS^2[799.4807]: 699.3916(17.35), 553.3347(100), 535.3234(23.70), 391.2823(90.94)	(B3-b)-glc-rha#	no. 20<br>no. 19	[1]
48	16.855 [a]	$C_{41}H_{70}O_{14}$	831.4711	831.4748	-4.45	$[M+HCOO]^-$	MS^2[831.4711]: 785.4690(76.96), 715.4031(46.56), 553.3407(49.45), 391.2856(100)<br>MS^3[831.4711→785.4691]: 491.3601(100)	B6-glc-xyl#	no. 20<br>no. 19<br>no. 25	[1]
49*	17.322 [b]	$C_{36}H_{62}O_{10}$	699.4340	699.4325	2.14	$[M+HCOO]^-$	MS^2[699.4343]: 653.4283(100), 491.3772(57.96), 391.2815(33.28)<br>MS^3[699.4343→653.4285]: 491.3615(100), 391.2779(99.68)	(B4-b)/B6-glc<br>(First found in ginseng)	no. 19	[1]N

no.	Retention Time	Molecular Formula	Measured (*m*/*z*)	Predicted (*m*/*z*)	Diff (ppm)	Ion	ESI-MS^n Data	Identification	Sample	Ref
50	17.393[a]	$C_{47}H_{80}O_{18}$	931.5208	931.5272	-6.87	$[M-H]^-$	MS^2[931.5206]: 799.4806(55.68), 637.4231(100), 619.4164(6.68), 475.3733(52.78), 391.2779(3.32)<br>MS^3[931.5206→637.4230]: 391.2779(100)	Ginsenoside Re_4 or its isomer	no. 20<br>no. 25<br>no. 19<br>no. 18	[1]
51[*]	17.445[a]	$C_{42}H_{74}O_{14}$	801.4982	801.5006	-2.99	$[M-H]^-$	MS^2[801.4980]: 655.4430(10.89), 637.4291(8.43), 493.3864(100), 417.3395(6.44)<br>MS^3[801.4980→655.4429]: 493.3820(100)	25-hydroxy-ginsenoside Rg_2 or ginsenoside Rf_2, or its isomer (First found in ginseng)	no. 20<br>no. 19	[1][N]
52	17.468[a]	$C_{41}H_{70}O_{14}$	831.4727	831.4748	-2.53	$[M+HCOO]^-$	MS^2[831.4730]: 785.4711(100), 653.4262(2.40)<br>MS^3[831.4730→785.4713]: 653.4101(38.00), 491.3753(100), 391.2812(97.97)<br>MS^4[831.4730→785.4713→653.4101]: 391.2779(100)	(B3-b)/(B6)-glc-xyl	no. 20<br>no. 25	[1]
53	18.360[a]	$C_{48}H_{82}O_{19}$	961.5310	961.5378	-7.07	$[M-H]^-$	MS^2[961.5306]: 799.4853(35.59), 637.4322(34.89), 475.3773(100), 391.2779(12.07)	20-glc-ginsenoside Rf or its isomer	no. 20<br>no. 19<br>no. 25	[1]
54[*]	18.458[a]	$C_{36}H_{62}O_{10}$	699.4285	699.4325	-5.72	$[M+HCOO]^-$	MS^2[699.4287]: 653.4196(100), 553.3346(67.48), 491.3776(13.10), 391.2716(11.22)<br>MS^3[699.4287→653.4199]: 491.3685(100)	(B4-b)/(B6)-glc (First found in ginseng)	no. 19<br>no. 20<br>no. 25	[1][N]
55	18.697[a]	$C_{42}H_{72}O_{14}$	799.4838	799.4849	-1.38	$[M-H]^-$	MS^2[799.4839]: 653.4204(23.16), 635.4090(14.05), 491.3732(100), 403.3181(7.11)<br>MS^3[799.4839→653.4205]: 491.3699(100)	B6-glc-rha (First found in ginseng)	no. 20<br>no. 19	[1][Q,N]
56	19.308[a]	$C_{53}H_{90}O_{22}$	1077.5771	1077.5851	-7.42	$[M-H]^-$	MS^2[1077.5769]: 945.5353(100), 799.4827(14.20), 783.4806(37.04), 637.4246(80.32), 619.4138(16.61), 475.3769(57.48), 391.2779(3.51)<br>MS^3[1077.5769→945.5348]: 637.4110(100), 457.3610(100)	Floraginsenoside M/N or its isomer	no. 20	[1]
57[**]	19.317[b]	$C_{48}H_{80}O_{18}$	989.5305	989.5327	-2.22	$[M+HCOO]^-$	MS^2[989.5299]: 943.5149(100)<br>MS^3[989.5299→943.5148]: 763.4574(100), 617.3911(15.50)	PPD-gluA-glc-rha (Structure not found in SciFinder)	no. 19	New
58[**]	20.045[a]	$C_{36}H_{64}O_{10}$	701.4468	701.4482	-2.00	$[M+HCOO]^-$	MS^2[701.4473]: 655.4324(100), 493.3847(58.09)<br>MS^3[701.4473→654.4171]: 493.3859(100), 392.2902(25.12)	(B1-b)-glc (Structure not found in SciFinder)	no. 20<br>no. 19<br>no. 25	New
59[*]	20.143[a]	$C_{42}H_{74}O_{14}$	801.5008	801.5006	0.25	$[M-H]^-$	MS^2[801.5004]: 655.4418(9.89), 637.4144(7.05), 493.3878(100), 417.3273(3.54)	Ginsenoside Rf_2 or its isomer (First found in ginseng)	no. 20	[3][R]
60	20.240[b]	$C_{47}H_{80}O_{18}$	931.5275	931.5272	0.32	$[M-H]^-$	MS^2[931.5271]799.4876(28.20), 637.4284(98.78), 475.3754(100), 391.2885(23.75)<br>MS^3[931.5271→475.3753]391.2967(100)	Notoginsenoside R_1/Ginsenoside R_1, or its isomer	no. 18<br>no. 19<br>no. 20<br>no. 25	[1]
61	20.247[a]	$C_{41}H_{70}O_{14}$	785.4693	785.4693	0	$[M-H]^-$	MS^2[785.4695]: 653.4223(38.48), 491.3714(100), 403.3283(3.44)<br>MS^3[785.4695→653.4226]: 491.3625(100)	(B4-b)/(B6)-glc-xyl[#]	no. 20	[1]
62[**]	20.270[a]	$C_{43}H_{72}O_{16}$	843.4735	843.4748	-1.54	$[M-H]^-$	MS^2[843.4733]: 799.4767(100)<br>MS^3[843.4733→799.4764]: 653.4222(44.40), 635.4042(44.40), 491.3685(88.79), 391.4842(100)	Unknown($C_{31}H_{52}O_7$)-glc-rha (Not found in SciFinder)	no. 20<br>no. 19	New
63	20.400[d]	$C_{48}H_{82}O_{19}$	961.5321	961.5378	-5.93	$[M-H]^-$	MS^2[961.5319]: 799.4765(34.88), 637.4329(65.93), 475.3839(100)	20-glc-ginsenoside Rf or its isomer	no. 25	[1]

no.	Retention Time	Molecular Formula	Measured (*m/z*)	Predicted (*m/z*)	Diff (ppm)	Ion	ESI-MS^n Data	Identification	Sample	Ref
64*	21.028[a]	$C_{36}H_{62}O_{10}$	699.4287	699.4325	-5.43	$[M+HCOO]^-$	MS^2[699.4288]: 653.4216(100), 491.3714(21.32)<br>MS^3[699.4288→653.4218]: 491.3761(100), 403.3136(9.04)	(B4-b)/(B6)-glc<br>(First found in ginseng)	no. 20<br>no. 19	[1][N]
65*	21.107[b,*]	$C_{48}H_{80}O_{18}$	943.5289	943.5272	1.80	$[M-H]^-$	MS^2[943.5287]: 763.4603(100), 617.4051(98.99)	PPD-gluA-glc-rha<br>Ginsenoside R_{18} or its isomer<br>(First found in ginseng)	no. 19	[1][Q]
66	21.275[a]	$C_{42}H_{72}O_{14}$	799.4826	799.4849	-2.88	$[M-H]^-$	MS^2[799.4825]: 653.4245(25.23), 635.4147(11.41), 491.3727(100), 391.2811(10.88)	(B4-b)/(B6)/(B3-b)-glc-rha[#]	no. 20<br>no. 19<br>no. 25	[1]
67	23.073[d]	$C_{54}H_{94}O_{25}$	570.2956	570.2969	-2.28	$[M-2H]^{2-}$	MS^2[570.2955(2)]: 979.5417(30.29), 817.4942(66.12), 799.4868(100), 655.4459(32.56), 493.3806(5.60)<br>MS^3[570.2955(2)→817.4937]: 655.4330(100), 493.3771(99.84)	Quinquenoside L_{16} or its isomer	no. 25	[1]
68	23.330[d]	$C_{48}H_{82}O_{19}$	1007.5382	1007.5432	-4.96	$[M+HCOO]^-$	MS^2[1007.5378]: 961.5257(100)<br>MS^3[1007.5378→961.5253]: 781.4607(39.53), 475.3701(100)	20-glc-ginsenoside Rf or its isomer	no. 25	[1]
69*	23.362[b]	$C_{36}H_{62}O_{10}$	699.4321	699.4325	-0.57	$[M+HCOO]^-$	MS^2[699.4320]: 653.4256(100), 491.3747(18.89)<br>MS^3[699.4320→653.4258]: 491.3717(100), 392.2977(9.49)	(B4-b)/(B6)-glc<br>(First found in ginseng)	no. 19	[1][N]
70	23.482[a]	$C_{53}H_{90}O_{22}$	1077.5766	1077.5851	-7.89	$[M-H]^-$	MS^2[1077.5769]: 945.5338(65.00), 799.4820(23.27), 783.4856(32.87), 637.4265(87.76), 475.3785(100)<br>MS^3[1077.5769→637.4264]: 475.3852(100)	Floralginsenoside N or its isomer	no. 20<br>no. 19	[1]
71	23.535[b]	$C_{42}H_{72}O_{14}$	845.4901	845.4904	-0.35	$[M+HCOO]^-$	MS^2[845.4897]: 799.4784(100)<br>MS^3[845.4897→799.4784]: 653.4343(6.26), 635.4042(6.26), 491.3705(100)	Pseudo-ginsenoside F_{11}, or its isomer	no. 19	[1]
72	24.750[c]	$C_{47}H_{80}O_{18}$	977.5343	977.5327	1.64	$[M+HCOO]^-$	MS^2[977.5343]: 931.5263(100), 799.5080(2.90), 637.4298(2.90)<br>MS^3[977.5343→931.5259]: 637.4298(74.94), 475.3748(100)	Notoginsenoside R_1 or its isomer	no. 18<br>no. 20<br>no. 25	[1]
73	26.267[a]	$C_{48}H_{80}O_{18}$	943.5219	943.5272	-5.62	$[M-H]^-$	MS^2[943.5217]: 797.4791(33.41), 635.4244(100), 617.3994(94.79), 473.3569(44.69)	A3-glc-glc-rha[#]	no. 20<br>no. 19	[1]
74**	26.555[b]	$C_{38}H_{66}O_{13}$	775.4551	775.4485	8.51	$[M+HCOO]^-$	MS^2[775.4549]: 729.4399(100)<br>MS^3[775.4549→730.4454]: 583.3904(33.33), 421.3375(100)	Unknown($C_{26}H_{46}O_4$)-glc-rha<br>(Structure not found in SciFinder)	no. 19	New
75	26.843[a]	$C_{53}H_{92}O_{24}$	1111.5833	1111.5906	-6.57	$[M-H]^-$	MS^2[1111.5831]: 979.5278(15.84), 817.4868(100), 799.4827(89.05), 655.4439(44.95), 637.4230(14.41), 493.3739(10.01)<br>MS^3[1111.5831→817.4865]: 493.3991(100)	Chikusetsusaponin FM_1 or its isomer	no. 20	[1]
76	27.890[c]	$C_{42}H_{72}O_{14}$	845.4924	845.4904	2.37	$[M+HCOO]^-$	MS^2[845.4923]: 799.4835(100), 637.4334(89.49), 619.4203(4.39), 475.3816(28.85)<br>MS^3[845.4923→799.4835]: 637.4308(100), 391.2853(17.36)	PPT-6-glc-20-glc<br>(Ginsenoside Rg_1)	no. 18<br>no. 20<br>no. 19<br>no. 25	
77	28.292[a]	$C_{48}H_{82}O_{19}$	961.5287	961.5260	-9.46	$[M-H]^-$	MS^2[961.5287]: 637.4271(100), 475.3852(29.04)	20-glc-ginsenoside Rf or its isomer	no. 20<br>no. 25	[1]

no.	Retention Time	Molecular Formula	Measured (*m/z*)	Predicted (*m/z*)	Diff (ppm)	Ion	ESI-MS^n Data	Identification	Sample	Ref
78	29.890[b]	$C_{41}H_{70}O_{14}$	831.4746	831.4748	-0.24	[M+HCOO]-	MS²[831.4743]: 785.4668(100)<br>MS³[831.4743→785.4670]: 491.3685(100)	(B3-b)/(B4-b)/B6/C2-glc-xyl	no. 19<br>no. 20	[1]
79	29.052[a]	$C_{48}H_{80}O_{20}$	1021.5190	1021.5225	-3.43	[M+HCOO]-	MS²[1021.5186]: 975.5063(100)<br>MS³[1021.5186→975.5060]: 868.4707(21.18), 653.4157(100), 633.3767(21.18), 491.3790(21.18)<br>MS⁴[1021.5186→975.5060→653.4158]: 491.3790(100)	(B8-b)-6-glc-glc-20-glc	no. 20	[1]
80	29.308[a]	$C_{42}H_{72}O_{15}$	861.4796	861.4853	-6.62	[M+HCOO]-	MS²[861.4793]: 815.4704(96.15), 797.4598(100), 651.4050(48.79)<br>MS³[861.4793→797.4595]:391.2779(100)	(C3-b)-glc-rha	no. 20	[1]
81*	30.072[a]	$C_{54}H_{90}O_{24}$	1121.5694	1121.5749	-4.90	[M-H]-	MS²[1121.5692]: 959.5147(97.94), 797.4622(100), 635.4042(16.86), 473.3618(52.23)<br>MS³[1121.5692→797.4620]: 389.2760(100)	A3-6-glc-glc-20-glc-glc<br>(First found in ginseng)	no. 20	[1][Q]
82*	30.463[a]	$C_{42}H_{72}O_{14}$	845.4863	845.4904	-4.85	[M+HCOO]-	MS²[845.4859]: 799.4803(100), 637.4318(12.14)<br>MS³[845.4859→799.4802]: 637.4230(100), 491.3664(100)	(B3-b)/(B4-b)/(B6)-glc-rha<br>(First found in ginseng)	no. 20	[1][Q,N]
83*	30.790[a]	$C_{47}H_{80}O_{18}$	931.5213	931.5272	-6.33	[M-H]-	MS²[931.5212]: 769.4734(25.65), 637.4324(100), 475.3761(100)	Isomer of notoginsenoside R_1<br>(First found in ginseng)	no. 20	[1][N]
84	34.212[a]	$C_{48}H_{82}O_{18}$	991.5405	991.5483	-7.87	[M+HCOO]-	MS²[991.5402]: 946.5390(100), 945.5391(47.59), 799.4952(3.80)<br>MS³[991.5402→945.5388]: 559.2262(66.67), 475.3783(100)	Isomer of ginsenoside Re	no. 20<br>no. 19	[1]
85	35.347[a]	$C_{48}H_{82}O_{18}$	945.5406	945.5428	-2.33	[M+HCOO]-	MS²[945.5401]: 799.4797(18.11), 783.4874(36.33), 637.4268(49.07), 619.4155(25.51), 475.3773(100), 391.2826(20.99)<br>MS³[945.5401→637.4268]: 553.3206(12.83), 475.3791(100)	PPT-6-glc-rha-20-glc (Ginsenoside Re)	no. 18<br>no. 20<br>no. 19<br>no. 25	
86	38.815[a]	$C_{48}H_{84}O_{20}$	979.5424	979.5483	-6.02	[M-H]-	MS²[979.5419]: 817.4923(100), 799.4796(45.92), 655.4397(52.53), 637.4224(16.72), 493.3824(6.77)<br>MS³[979.5419→817.4922]: 655.4341(100), 493.3859(78.94)<br>MS⁴[979.5419→817.4922→655.4341]: 493.3876(100), 375.2964(47.62)	Vinaginsenoside R_{13} or its isomer	no. 20	[1]
87*	39.873[a]	$C_{36}H_{62}O_{10}$	653.4247	653.4270	-3.52	[M-H]-	MS²[653.4248]: 521.3275(33.49), 491.3738(100), 391.2842(37.52)	(B4-b)/(B6)-glc<br>(First found in ginseng)	no. 20<br>no. 19	[1][N]
88*	40.127[a]	$C_{35}H_{56}O_{9}$	665.3882	655.3906	-3.61	[M+HCOO]-	MS²[665.3881]: 619.3816(100), 457.3330(83.87)	Unknown($C_{29}H_{48}O_4$)-glc<br>(First found in ginseng)	no. 20	
89*	40.195[a]	$C_{36}H_{62}O_{10}$	699.4306	699.4325	-2.72	[M+HCOO]-	MS²[699.4307]: 653.4194(100), 491.3714(36.05), 391.2748(6.72)<br>MS³[699.4307→653.4197]: 491.3779(100)	(B4-b)/(B6)-glc<br>(First found in ginseng)	no. 20	[1][N]
90*	40.242[a]	$C_{48}H_{80}O_{18}$	943.5258	943.5272	-1.48	[M-H]-	MS²[943.5255]: 799.4829(28.22), 783.4897(55.64), 637.4265(100), 619.4152(41.13), 475.3760(76.80)<br>MS³[943.5255→637.4264]: 475.3694(100), 353.2473(56.34)	(A2-a)-glc-glc-glc<br>(First found in ginseng)	no. 20	
91*	40.478[a]	$C_{42}H_{70}O_{12}$	765.4433	765.4795	-47.29	[M-H]-	MS²[765.4331]: 619.3849(8.63), 457.3297(100), 415.3153(3.04)<br>MS³[765.4331→619.3849]: 457.3205(100)	Ginsenoside Rg_{31} or its isomer<br>(First found in ginseng)	no. 20<br>no. 19	[1][Q,N]
92	40.605[a]	$C_{42}H_{70}O_{14}$	843.4718	843.4748	-3.56	[M+HCOO]-	MS²[843.4717]: 797.4668(100), 635.4104(37.69), 617.3823(13.81), 473.3569(24.21)<br>MS³[843.4717→797.4666]: 635.4281(100), 473.3569(66.67)	A3-glc-glc<br>(First found in ginseng)	no. 20	[1]

no.	Retention Time	Molecular Formula	Measured (m/z)	Predicted (m/z)	Diff (ppm)	Ion	ESI-MS^n Data	Identification	Sample	Ref
93	40.677[a]	$C_{48}H_{82}O_{18}$	945.5376	945.5428	-5.50	$[M-H]^-$	MS^2[945.5373]: 799.4862(37.72), 783.4946(24.95), 637.4240(100), 619.4164(17.77), 475.3771(86.41), 391.2833(5.69)	Isomer of ginsenoside Re	no. 20	[1]
94*	40.913[b]	$C_{36}H_{62}O_{10}$	699.4335	699.4325	1.43	$[M+HCOO]^-$	MS^2[699.4340]: 653.4231(99.55), 491.3776(100)<br>MS^3[699.4340→491.3777]: 403.3212(100)	B6/(B4-b)-glc<br>(First found in ginseng)	no. 19	[1][N]
95**	40.932[a]	$C_{41}H_{68}O_{13}$	768.4657	768.466	-0.37	$[M+HCOO]^-$	MS^2[813.4639]: 767.4578(100), 635.4058(34.42), 489.3553(4.17)	(B8-b)-rha-xyl<br>(Structure not found in SciFinder)	no. 20<br>no. 19	New
96**	41.082[a]	$C_{54}H_{92}O_{24}$	561.2898	561.2917	-3.39	$[M-2H]^{2-}$	MS^2[561.2900(2)]: 961.5367(89.98), 799.4831(88.49), 781.4794(100), 637.4221(54.76), 475.3830(47.19), 375.2964(7.86)	(B4-a)-6-glc-20-glc(-glc)-glc<br>(Structure not found in SciFinder)	no. 20	New
97*	41.228[a]	$C_{48}H_{80}O_{19}$	959.5170	959.5221	-5.32	$[M-H]^-$	MS^2[959.5168]: 797.4654(74.48), 635.4148(22.34), 473.3640(100), 389.2699(23.22)<br>MS^3[959.5168→797.4651]: 635.4161(36.17), 473.3662(100), 389.2760(36.17)	Notoginsenoside G or its isomer	no. 20	
98	41.593[a]	$C_{53}H_{90}O_{23}$	1093.5762	1093.5800	-3.47	$[M-H]^-$	MS^2[1093.5757]: 961.5298(41.13), 799.4782(100), 781.4700(56.86), 637.4247(63.32), 475.3744(62.94)<br>MS^3[1093.5757→799.4780]: 637.4226(70.56), 475.3782(100), 391.2405(4.57)<br>MS^4[1093.5757→799.4780→637.4225]: 475.3700(100)	PPT-6-glc-xyl-20-glc-glc	no. 20<br>no. 19	[1]
99	41.617[c]	$C_{41}H_{70}O_{13}$	815.4827	815.4798	3.56	$[M+HCOO]^-$	MS^2[815.4829]: 769.4734(100), 619.4248(44.63)<br>MS^3[815.4829→769.4729]: 619.4254(100), 476.3833(66.45)	PPT-glc-xyl	no. 18<br>no. 20	[1]
100	42.017[a]	$C_{53}H_{90}O_{23}$	1093.5766	1093.5800	-3.11	$[M-H]^-$	MS^2[1093.5762]: 961.5317(29.12), 799.4778(90.05), 781.4718(75.72), 637.4244(100), 475.3760(81.60)<br>MS^3[1093.5762→637.4243]: 475.3769(100), 387.3239(13.62), 375.3872(12.74)	(B4-a)-6-glc-xyl-20-glc-glc	no. 20<br>no. 19	[1]
101	42.380[b]	$C_{41}H_{70}O_{13}$	815.4801	815.4798	0.37	$[M+HCOO]^-$	MS^2[815.4803]: 769.4711(51.08), 655.4431(15.47), 637.4266(100), 475.3777(35.95)<br>MS^3[815.4803→637.4264]: 475.3886(100)	PPT-glc-xyl	no. 18<br>no. 19<br>no. 20<br>no. 25	[1]
102	42.568[a]	$C_{44}H_{74}O_{15}$	887.4971	887.5010	-4.39	$[M+HCOO]^-$	MS^2[887.4969]: 841.4861(29.53), 799.4873(14.18), 781.4734(100), 637.4224(9.02), 619.4169(32.27)<br>MS^3[887.4969→781.4735]: 619.4142(100), 475.3989(23.27)	AcO-ginsenoside Rg_1	no. 20<br>no. 18<br>no. 25	[1]
103	42.743[a]	$C_{53}H_{90}O_{23}$	1093.5754	1093.5800	-4.21	$[M-H]^-$	MS^2[1093.5754]: 961.5257(20.12), 799.4768(74.57), 781.4682(59.33), 637.4233(100), 475.3762(76.80)<br>MS^3[1093.5754→637.4229]: 475.3789(100), 387.3351(13.70)	PPT-6-glc-glc-20-glc-xyl	no. 20	[1]
104	42.767[a]	$C_{48}H_{80}O_{18}$	943.5208	943.5272	-6.78	$[M-H]^-$	MS^2[943.5207]: 797.4645(100), 635.4053(33.15), 617.4119(24.18), 473.3672(12.09)	(B8-a)-3-glc-20-glc-rha	no. 20	[1]
105	42.860[a]	$C_{42}H_{74}O_{15}$	817.4945	817.4955	-1.22	$[M-H]^-$	MS^2[817.4943]: 655.4359(100), 493.3860(52.55), 375.2859(2.64)<br>MS^3[817.4943→655.4360]: 537.3410(4.23), 493.3903(100), 491.3708(13.53), 399.3244(6.26), 375.2903(29.94)	(B2-a)-3-glc-20-glc	no. 20	[1]
106	42.868[b]	$C_{48}H_{82}O_{19}$	961.5363	961.5378	-1.56	$[M-H]^-$	MS^2[961.5361]: 799.4816(88.88), 781.4715(37.96), 637.4258(100), 475.3777(76.25)<br>MS^3[961.5361→637.4255]: 475.3812(100), 387.3258(19.20), 375.2808(10.86)	PPT-6-glc-20-glc-glc	no. 19<br>no. 18	[1]
107	42.888[a]	$C_{48}H_{80}O_{19}$	959.5172	959.5221	-5.11	$[M-H]^-$	MS^2[959.5170]: 797.4630(58.15), 635.4143(44.11), 473.3577(100)	(A3)/(B8-a)-3-glc-20-glc-glc	no. 20	[1]

no.	Retention Time	Molecular Formula	Measured (*m/z*)	Predicted (*m/z*)	Diff (ppm)	Ion	ESI-MS^n Data	Identification	Sample	Ref
108	42.945[a]	$C_{48}H_{82}O_{19}$	961.5326	961.5378	-5.41	$[M-H]^-$	MS^2[961.5323]: 799.4811(100), 781.4726(36.88), 637.4254(97.21), 619.4170(13.93), 537.3452(10.32), 475.3755(66.10), 387.3289(5.43)<br>MS^3[961.5323→799.4811]: 637.4269(90.25), 475.3786(100), 375.3789(7.54)<br>MS^4[961.5323→799.4811→637.4266]: 475.3783(100), 387.3227(42.88), 375.2780(28.49)	B5-3-glc-glc-20-glc	no. 20<br>no. 19<br>no. 25	[1]
109	43.030[a]	$C_{42}H_{72}O_{14}$	799.4849	799.4849	0	$[M-H]^-$	MS^2[799.4847]: 637.4303(100), 475.3783(69.74)<br>MS^3[799.4847→637.4302]: 387.3165(100)	Isomer of ginsenoside Rf	no. 20	[1]
110*	43.527[a]	$C_{42}H_{70}O_{14}$	843.4712	843.4748	-4.27	$[M+HCOO]^-$	MS^2[843.4708]: 797.4677(100)<br>MS^3[843.4708→797.4674]: 635.4042(100), 473.3569(33.33)<br>MS^4[843.4708→797.4674→635.4041]: 473.3466(100)	(B8-a)/(A3)-3-glc-20-glc<br>(First found in ginseng)	no. 20<br>no. 19<br>no. 25	[1][Q,N]
111*	43.583[a]	$C_{36}H_{60}O_{10}$	697.4142	697.4169	-6.45	$[M+HCOO]^-$	MS^2[697.4125]: 651.4100(100), 489.3762(8.78)	(B8-b)-glc<br>(First found in ginseng)	no. 20<br>no. 19	[1][N]
112*	43.865[a]	$C_{36}H_{62}O_{10}$	699.3946	-	-	$[M+HCOO]^-$	MS^2[699.3948]: 653.4322(14.84), 537.3404(100), 483.3173(11.35), 375.2888(70.46)	(C2)/(C3-a)-glc	no. 20<br>no. 19	[1][N]
113	43.997[a]	$C_{42}H_{72}O_{13}$	783.4899	783.4900	-0.13	$[M-H]^-$	MS^2[783.4902]: 637.4299(100), 619.4354(26.75), 475.3783(30.48), 417.3342(39.72)	Isomer of ginsenoside Rg_2	no. 20	[1]
114**	44.095[a]	$C_{39}H_{68}O_{14}$	805.4600	805.4591	1.12	$[M+HCOO]^-$	MS^2[805.4597]: 759.4501(100)<br>MS^3[805.4597→759.4501]: 597.4013(31.25), 435.3427(100), 375.2780(7.99)<br>MS^4[805.4597→759.4501→597.4014]: 435.3394(100)	Unknown($C_{27}H_{48}O_4$)-glc-glc<br>(Structure not found in SciFinder)	no. 20<br>no. 19	New
115	44.303[a]	$C_{47}H_{80}O_{18}$	931.5238	931.5272	-3.65	$[M-H]^-$	MS^2[931.5237]: 799.4823(69.50), 769.4728(24.40), 637.4217(31.30), 537.3410(20.95), 475.3773(100), 353.1049(62.60), 329.1969(20.95)	Isomer of notoginsenoside R_1	no. 20<br>no. 19	[1]
116	44.393[c]	$C_{54}H_{86}O_{24}$	1117.5424	1117.5436	-1.07	$[M-H]^-$	MS^2[1117.5422]: 793.4384(66.23), 613.3742(100), 569.3845(39.88), 523.3779(65.17), 453.3377(44.76)	OA-gluA-glc-glc-glc	no. 18	[1]
117	44.545[d]	$C_{60}H_{102}O_{28}$	634.3178	634.3206	-4.41	$[M-2H]^{2-}$	MS^2[634.3178(2)]: 1107.5885(32.77), 945.5358(53.31), 783.4851(100), 765.4724(23.88), 621.4329(40.03)<br>MS^3[634.3178(2)→783.4855]: 459.3886(100)	Quinquenoside V or its isomer	no. 25<br>no. 20	[1]
118*	44.640[a]	$C_{42}H_{70}O_{13}$	827.4775	827.4798	-2.78	$[M+HCOO]^-$	MS^2[827.4774]: 781.4713(100)<br>MS^3[827.4774→781.4650]: 617.3911(100)	(A2-a)-glc-rha<br>(First found in ginseng)	no. 20<br>no. 19<br>no. 18	[1][Q,N]
119	44.745[a]	$C_{64}H_{108}O_{31}$	685.3338	685.3365	-3.94	$[M-2H]^{2-}$	MS^2[685.3341(2)]: 1209.6371(22.77), 1077.5868(36.83), 945.5383(82.81), 783.4909(100), 621.4332(22.77), 619.8172(34.15), 553.2947(65.74), 485.1597(95.04)	Notoginsenoside D or its isomer	no. 20	[1]
120	44.873[b]	$C_{48}H_{82}O_{17}$	975.5504	975.5534	-3.08	$[M+HCOO]^-$	MS^2[975.5504]: 929.5354(100)<br>MS^3[975.5504→929.5352]: 621.4332(66.45), 475.3852(100)	PPT-rha-glc-rha[#]	no. 20<br>no. 19	[1]
121	44.980[a]	$C_{42}H_{70}O_{14}$	843.4713	843.4748	-4.15	$[M+HCOO]^-$	MS^2[843.4710]: 797.4637(100)<br>MS^3[843.4710→797.4634]: 473.3569(100)	A3-glc-glc	no. 20<br>no. 19	[1]
122**	45.002[b]	$C_{38}H_{64}O_{14}$	743.4226	743.4223	0.40	$[M-H]^-$	MS^2[743.4225]: 597.3617(70.84), 435.3087(100), 417.2935(7.98)	Unknown($C_{26}H_{44}O_5$)-glc-rha<br>(Structure not found in SciFinder)	no. 19<br>no. 20	New

no.	Retention Time	Molecular Formula	Measured (*m/z*)	Predicted (*m/z*)	Diff (ppm)	Ion	ESI-MS^n Data	Identification	Sample	Ref
123	45.425^a	$C_{59}H_{100}O_{27}$	1239.6376	1239.6379	-0.24	$[M-H]^-$	MS²[1239.6375]: 1107.5838(100), 945.5309(83.40), 783.4846(52.15), 621.4318(23.25), 459.3808(11.46)<br>MS³[1239.6375→1107.5840]: 945.5307(100), 783.4961(43.05), 621.4271(70.40), 459.3886(37.68)<br>MS⁴[1239.6375→1107.5840→945.5305]: 460.3736(100)	Notoginsenoside R_4 or its isomer	no. 18<br>no. 20<br>no. 19<br>no. 25	[1]
124*	45.683^a	$C_{41}H_{70}O_{14}$	785.4694	785.4693	0.13	$[M-H]^-$	MS²[785.4698]: 653.4215(100), 491.3782(8.56), 415.3236(9.22)	Vinaginsenoside R_{11} or its isomer (First found in ginseng)	no. 20<br>no. 19<br>no. 18	
125	45.753^a	$C_{42}H_{74}O_{15}$	863.4962	863.5010	-5.56	$[M+HCOO]^-$	MS²[863.4960]: 817.4885(100)<br>MS³[863.4960→817.4882]: 655.4320(100), 493.3844(29.03)<br>MS⁴[863.4960→817.4882→655.4320]: 493.3971(100), 375.2859(46.83)	(B2-a)/C4-glc-glc	no. 20	[1]
126	45.922^a	$C_{54}H_{86}O_{24}$	1117.5414	1117.5436	-1.97	$[M-H]^-$	MS²[1117.5415]: 793.4316(75.86), 613.3667(100), 569.3816(47.60), 523.3750(74.36), 455.3489(42.25)	ginsenoside Ro_a or its isomer	no. 20	[1]
127	46.468^a	$C_{42}H_{72}O_{14}$	799.4835	799.4849	-1.75	$[M-H]^-$	MS²[799.7304]: 653.4207(100), 635.4084(40.75), 491.3700(52.80), 415.3195(66.50)	(B4-b)-3-glc-20-rha	no. 20<br>no. 19	[1]
128*	46.768^b	$C_{36}H_{62}O_{10}$	699.4324	699.4325	-0.14	$[M+HCOO]^-$	MS²[699.4326]: 654.4272(100)<br>MS³[699.4326→653.4243]: 491.3895(100)	(B3-b)/B6-glc (First found in ginseng)	no. 19<br>no. 20	[1]N
129	46.813^b	$C_{42}H_{72}O_{14}$	799.4871	799.4849	2.75	$[M-H]^-$	MS²[799.4871]: 637.4270(29.39), 475.3768(100), 391.2836(47.88)<br>MS³[799.4871→475.3769]: 391.2847(100)	PPT-6-glc-glc (Ginsenoside Rf)	no. 18<br>no. 19<br>no. 20<br>no. 25	
130	46.827^c	$C_{54}H_{90}O_{23}$	552.2868	552.2864	0.72	$[M-2H]^{2-}$	MS²[552.2867(2)]: 943.5224(100), 781.4754(96.11), 619.3990(6.77), 457.3568(5.12)<br>MS³[552.2867(2)→945.5223]: 782.4642(85.71), 457.3655(100)	(B7-a)-3-glc-glc-20-glc-glc	no. 18	[1]
131	47.788^a	$C_{58}H_{98}O_{26}$	1209.6248	1209.6274	-2.15	$[M-H]^-$	MS²[1209.6248]: 1077.5747(100), 1078.5756(69.03), 945.5338(28.46), 915.5240(14.02), 783.4842(34.05), 765.4759(11.79), 621.4310(23.88), 459.3793(4.82)<br>MS³[1209.6248→1077.5746]: 945.5284(53.33), 783.4818(100), 621.4302(84.66), 537.3392(8.36), 459.3795(50.57), 375.2857(5.28)<br>MS⁴[1209.6248→1077.5746→783.4822]: 622.4370(100), 460.3837(67.10), 376.2878(67.10), 375.2964(67.10)	Notoginsenoside Fc or its isomer	no. 20<br>no. 19<br>no. 25	[1]
132	48.038^a	$C_{59}H_{100}O_{27}$	1239.6355	1239.6379	-1.94	$[M-H]^-$	MS²[1239.6353]: 1107.5865(100), 1077.5757(20.28), 945.5373(66.13), 783.4870(47.48), 621.4329(17.37), 459.3828(8.87)<br>MS³[1239.6353→1107.5868]: 945.5374(100), 783.4876(75.67), 621.4463(38.56), 459.3886(11.71)	Notoginsenoside R_4 or its isomer	no. 20<br>no. 19<br>no. 25	[1]
133	48.095^a	$C_{48}H_{80}O_{18}$	943.5238	943.5272	-3.60	$[M-H]^-$	MS²[943.5235]: 781.4676(100), 619.4196(74.67), 601.4008(11.80)<br>MS³[943.5235→781.4678]: 620.4185(100), 619.3928(100), 458.3742(100)	(A2-a)/B9-glc-glc-glc#	no. 20	[1]
134	48.265^b	$C_{54}H_{92}O_{23}$	1107.5966	1107.5957	0.81	$[M-H]^-$	MS²[1107.5964]: 945.5356(98.28), 783.4876(100), 621.4320(47.67), 459.3814(26.18)<br>MS³[1107.5964→783.4879]: 621.4325(100), 537.3520(14.91), 459.3874(93.57), 441.3813(14.91), 375.2849(29.90)	PPD-3-glc-glc-20-glc-glc (Ginsenoside Rb_1)	no. 18<br>no. 19<br>no. 20<br>no. 25	

no.	Retention Time	Molecular Formula	Measured (m/z)	Predicted (m/z)	Diff (ppm)	Ion	ESI-MS^n Data	Identification	Sample	Ref
135	48.562[b]	$C_{41}H_{70}O_{13}$	815.4800	815.4798	0.25	$[M+HCOO]^-$	MS^2[815.4801]: 769.4729(100), 637.4260(33.18), 475.3813(17.67)<br>MS^3[815.4801→769.4729]: 637.4264(65.17), 476.3780(100), 475.3752(68.64), 391.2779(13.74)	Ginsenoside F_3, Notoginsenoside R_2, or their isomer	no. 18<br>no. 20<br>no. 19<br>no. 25	[1]
136	49.537[a]	$C_{58}H_{98}O_{26}$	1209.6171	1209.6274	-8.52	$[M-H]^-$	MS^2[1209.6169]: 1077.5698(100), 1047.5624(11.25), 945.5320(56.48), 915.5181(24.72), 783.4850(73.94), 621.4313(31.71), 459.3814(8.92)<br>MS^3[1209.6169→1077.5898]: 945.5375(54.18), 915.5164(14.81), 784.4817(100), 783.4964(96.50), 621.4309(35.87), 459.3886(21.81)	Ginsenoside Ra_1/Ra_2 or its isomer	no. 20<br>no. 19	[1]
137	49.583[b]	$C_{53}H_{90}O_{22}$	1077.5844	1077.5851	-0.65	$[M-H]^-$	MS^2[1077.5844]: 945.5385(69.94), 783.4885(100), 621.4330(66.91), 459.3818(28.27)<br>MS^3[1077.5844→783.4888]: 621.4308(82.78), 537.3338(9.92), 459.3815(100), 375.2811(48.02)	Ginsenoside Rc or its isomer	no. 18<br>no. 20<br>no. 19<br>no. 25	[1]
138	48.935[c]	$C_{57}H_{94}O_{26}$	1193.5982	1193.5961	1.76	$[M-H]^-$	MS^2[1193.5980]: 1150.6069(100), 1049.5938(26.85), 1108.6275(22.93)<br>MS^3[1193.5980→1149.5938]: 1108.5834(100), 1107.5558(80.46), 927.5289(80.46)	Malonyl-Ginsenoside Rb_1, or its isomer	no. 18	[1]
139	50.287[a]	$C_{58}H_{98}O_{26}$	604.3051	604.3100	-8.11	$[M-2H]^{2-}$	MS^2[604.3054(2)]: 945.5319(20.10), 783.4839(100), 765.4747(39.97), 621.4330(17.7), 539.2935(20.39), 459.3790(4.87)<br>MS^3[604.3054(2)→783.4839]: 621.4349(53.80), 459.3864(100)	Ginsenoside Ra_1, Ra_2, or their isomer	no. 20<br>no. 25	[1]
140	50.525[c]	$C_{48}H_{76}O_{19}$	955.4944	955.4908	3.77	$[M-H]^-$	MS^2[955.4945]: 793.4390(100), 731.4397(3.64), 613.3748(36.86), 569.3851(29.55), 523.3788(66.91), 455.3520(11.71)<br>MS^3[955.4945→793.4390]: 613.3725(100), 569.3812(51.96), 523.3794(80.95), 455.3520(35.06)	Ginsenoside Ro or its isomer	no. 18<br>no. 20<br>no. 25	[1]
141	50.775[a]	$C_{58}H_{98}O_{26}$	1209.6176	1209.6274	-8.10	$[M-H]^-$	MS^2[1209.6176]: 1077.5754(100), 945.5341(50.61), 783.4888(63.40), 621.4305(34.61), 459.3799(13.60)<br>MS^3[1209.6176→1077.5753]: 945.5356(28.64), 783.4847(23.82), 621.4233(100), 459.3799(33.36)	Ginsenoside Ra_1/Ra_2 or its isomer	no. 20<br>no. 25	[1]
142	50.932[b]	$C_{36}H_{62}O_9$	683.4387	683.4376	1.61	$[M+HCOO]^-$	MS^2[683.4391]: 637.4304(100), 475.3793(89.06)<br>MS^3[683.4391→637.4302]: 391.2779(100)	Ginsenoside Rh_1	no. 18<br>no. 19<br>no. 20<br>no. 25	
143	50.950[a]	$C_{42}H_{72}O_{13}$	783.4929	783.4900	3.70	$[M-H]^-$	MS^2[783.4929]: 637.4242(21.25), 619.4127(13.06), 475.3760(100), 391.2831(34.21)<br>MS^3[783.4929→637.4241]: 553.3407(13.74), 475.3793(100), 391.2967(27.47)	Ginsenoside Rg_2	no. 18<br>no. 20<br>no. 19<br>no. 25	
144	51.103[a]	$C_{53}H_{90}O_{22}$	1077.5817	1077.5851	-3.16	$[M-H]^-$	MS^2[1077.5819]: 945.5350(57.44), 783.4874(100), 621.4314(55.00), 459.3810(30.35)<br>MS^3[1077.5819→783.4875]: 621.4253(100), 459.3821(94.78), 375.2859(18.47)<br>MS^4[1077.5819→783.4875→621.4255]: 459.3683(100)	Ginsenoside Rb_2	no. 18<br>no. 19<br>no. 20<br>no. 25	[1]
145	51.155[a]	$C_{42}H_{72}O_{13}$	783.4870	783.4900	-3.83	$[M-H]^-$	MS^2[783.4872]: 637.4265(22.92), 475.3782(100), 391.2856(35.25)<br>MS^3[783.4872→637.4263]: 553.3519(66.67), 475.3680(100)	Isomer of ginsenoside Rg_2	no. 20	[1]

no.	Retention Time	Molecular Formula	Measured (*m/z*)	Predicted (*m/z*)	Diff (ppm)	Ion	ESI-MS^n Data	Identification	Sample	Ref
146	51.482[c]	$C_{36}H_{62}O_9$	683.4370	683.4376	-0.88	$[M+HCOO]^-$	MS^2[683.4368]: 637.4232(100), 549.7537(21.61), 475.3838(46.40)	Isomer of ginsenoside Rh_1	no. 18<br>no. 20<br>no. 19	[1]
147	52.018[c]	$C_{47}H_{74}O_{18}$	925.4810	925.4802	0.86	$[M-H]^-$	MS^2[925.4808]: 763.4280(71.25), 613.3742(100), 569.3845(29.67), 523.3789(25.36), 455.3492(20.05)	Pseudoginsenoside Rt_1 or its isomer	no. 18	[1]
148	52.190[a]	$C_{36}H_{62}O_{12}$	683.4248	683.4223	3.66	$[M+HCOO]^-$	MS^2[683.4245]: 637.4255(100), 475.3772(88.86)<br>MS^2[683.4245→637.4254]: 553.3318(21.91), 475.3813(100), 391.2873(5.83)	Ginsenoside F_1	no. 20<br>no. 19	[1]
149	51.910[a]	$C_{58}H_{98}O_{26}$	604.3042	604.3100	-9.60	$[M-2H]^{2-}$	MS^2[604.3046(2)]: 945.5371(17.37), 783.4873(100), 765.4705(10.48), 621.4317(12.70)<br>MS^3[604.3046(2)→783.4873]: 621.4355(20.09), 459.3826(100), 375.2780(16.14)	Ginsenoside Ra_1/Ra_2 or its isomer	no. 20	[1]
150*	52.235[a,*]	$C_{42}H_{74}O_{14}$	801.4942	801.5006	-7.99	$[M-H]^-$	MS^2[801.4942]: 639.4484(19.88), 477.3935(100), 401.3350(2.33)<br>MS^3[801.4942→638.4295]: 477.3937(100)	25-hydroxyl-ginsenoside Rg_3 or its isomer<br>(First found in ginseng)	no. 20	
151	52.315[a]	$C_{54}H_{92}O_{23}$	1107.5861	1107.5957	-8.67	$[M-H]^-$	MS^2[1107.5863]: 945.5317(55.40), 783.4852(100), 621.4295(91.07), 459.3814(7.25)<br>MS^3[1107.5863→783.4855]: 621.4355(100), 459.3845(29.69)	Ginsenoside B_1 or its isomer	no. 20	[1]
152	52.687[c]	$C_{47}H_{74}O_{18}$	925.4827	925.4802	2.70	$[M-H]^-$	MS^2[925.4826]: 763.4274(100), 613.3722(65.16), 569.3850(31.55), 523.3794(35.05), 455.3522(23.36)<br>MS^3[925.4826→763.4274]: 613.3679(100), 569.3860(12.55), 523.3864(21.97), 455.3478(44.00)	Pseudoginsenoside Rt_1 or its isomer	no. 18	[1]
153	52.782[a]	$C_{48}H_{80}O_{18}$	944.5307	944.5345	-4.03	$[M-H]^-$	MS^2[943.5230]: 781.4643(100), 619.4057(23.97), 457.3598(58.82)	B9-3-glc-20-glc-glc	no. 20	[1]
154	52.965[a]	$C_{52}H_{88}O_{21}$	1047.5636	1047.5745	-10.41	$[M-H]^-$	MS^2[1047.5637]: 915.5264(100), 753.4780(41.85), 735.4666(5.40), 621.4340(82.63), 459.3808(33.88)<br>MS^3[1047.5637→915.5262]: 537.3191(100), 459.3683(100)	PPD-3-glc-xyl-20-glc-xyl	no. 20<br>no. 18	[1]
155	53.087[a]	$C_{42}H_{70}O_{14}$	797.4703	797.4693	1.25	$[M-H]^-$	MS^2[797.4705]: 635.4079(100), 473.3595(82.77)<br>MS^3[797.4705→635.4080]: 473.3569(100)	(B8-a)/A3-6-glc-20-glc	no. 20	[1]
156	53.670[c]	$C_{36}H_{62}O_9$	683.4410	683.4376	4.97	$[M+HCOO]^-$	MS^2[683.4409]: 637.4280(100), 475.3742(73.51), 389.2277(4.27)	PPT-glc	no. 18	[1]
157	53.702[a]	$C_{53}H_{90}O_{22}$	1077.5779	1077.5851	-6.68	$[M-H]^-$	MS^2[1077.5776]: 945.5288(25.31), 915.5169(27.41), 783.4832(100), 621.4298(79.85), 459.3822(11.56)<br>MS^3[1077.5776→783.4833]: 621.4388(100), 459.3886(18.20)<br>MS^4[1077.5776→783.4833→621.4388]: 459.3886(100)	Isomer of ginsenoside Rc	no. 20	[1]
158	53.862[a]	$C_{48}H_{82}O_{17}$	929.5423	929.5479	-6.02	$[M-H]^-$	MS^2[929.5423]: 767.4948(60.29), 605.4325(100)	PPD-glc-glc-rha[#]	no. 20	[1]
159	54.120[b]	$C_{48}H_{82}O_{18}$	991.5475	991.5483	-0.81	$[M+HCOO]^-$	MS^2[991.5477]: 945.5371(100)<br>MS^3[991.5477→945.5369]: 783.4741(44.25), 459.3773(100)	Ginsenoside Rd	no. 18<br>no. 20<br>no. 19<br>no. 25	
160	54.488[a]	$C_{48}H_{82}O_{18}$	945.5392	945.5428	-3.81	$[M-H]^-$	MS^2[945.5392]: 783.4855(100), 621.4311(48.31), 459. 3791(36.72)<br>MS^3[945.5392→783.4857]: 621.4330(60.42), 459.3854(100), 375.2907(43.69)<br>MS^4[945.5392→783.4857→621.4330]: 375.3074(100)	Isomer of ginsenoside Rd	no. 20	[1]

no.	Retention Time	Molecular Formula	Measured (*m/z*)	Predicted (*m/z*)	Diff (ppm)	Ion	ESI-MSⁿ Data	Identification	Sample	Ref
161	55.133[c]	$C_{42}H_{66}O_{14}$	793.4402	793.4380	2.77	$[M-H]^-$	MS²[793.4402]: 631.3849(100), 455.3546(10.07)<br>MS³[793.4402→631.3849]: 455.3548(100)	OA-3-glc-28-glc	no. 18	[1]
162	55.617[a]	$C_{42}H_{72}O_{14}$	845.4878	845.4904	-3.08	$[M+HCOO]^-$	MS²[845.4877]: 799.4804(100)<br>MS³[845.4877→799.4805]: 637.4230(100)	Isomer of ginsenoside Rg_1	no. 20<br>no. 19	[1]
163	55.777[a]	$C_{55}H_{92}O_{23}$	1119.5922	1119.5957	-3.13	$[M-H]^-$	MS²[1119.5922]: 1077.5786(100), 1059.5667(72.08), 945.5346(12.22), 783.4875(9.73), 765.4759(9.56)<br>MS³[1119.5922→1077.5786]: 945.5334(80.77), 783.4894(100), 621.4307(87.40), 459.3825(39.61), 375.2964(7.99)	PPD-3-glc-glc-20-glc-xyl-AcO	no. 20<br>no. 19	[1]
164	56.507[a]	$C_{58}H_{96}O_{24}$	587.3034	587.3073	-6.64	$[M-2H]^{2-}$	MS²[587.3036(2)]: 945.5321(100), 783.4888(72.22), 621.4450(7.77), 553.2942(82.27)<br>MS³[587.3036(2)→945.5317]: 783.4741(100)	Ginsenoside IV or its isomer	no. 20<br>no. 19<br>no. 25	[1]
165	56.562[c]	$C_{47}H_{74}O_{18}$	925.4817	925.4802	1.62	$[M-H]^-$	MS²[925.4816]: 793.4427(100), 613.3709(10.56), 569.3851(22.63), 523.3783(52.83), 453.3377(9.71)<br>MS²[925.4816→793.4425]: 613.3835(100), 569.3747(84.62)	OA-gluA-glc-xyl[#]	no. 18	[1]
166	56.610[a]	$C_{41}H_{66}O_{13}$	765.4454	765.4431	3.00	$[M-H]^-$	MS²[765.4458]: 603.3934(26.34), 441.3342(100)	Unknown($C_{29}H_{46}O_3$)-glc<br>(First found in ginseng)	no. 20<br>no. 19	
167	56.767[b]	$C_{42}H_{72}O_{14}$	845.4894	845.4904	-1.18	$[M+HCOO]^-$	MS²[845.4892]: 799.4773(100)<br>MS³[845.4885→799.4772]: 475.3731(100)	Isomer of ginsenoside Rg_1	no. 19<br>no. 20	[1]
168	56.835[a]	$C_{48}H_{82}O_{18}$	945.5396	945.5428	-3.38	$[M-H]^-$	MS²[945.5397]: 783.4877(80.68), 621.4339(100), 459.3880(60.51)<br>MS³[945.5397→621.4340]: 460.4041(100)	PPD-6-glc-glc-20-glc	no. 20<br>no. 19<br>no. 25	[1]
169	57.313[a]	$C_{42}H_{66}O_{14}$	793.4404	793.4380	3.02	$[M-H]^-$	MS²[793.4404]: 631.3821(100), 455.3504(21.83)<br>MS³[793.4404→631.3819]: 455.3499(100)<br>MS⁴[793.4404→631.3819→633.3870]: 457.4015(100)	OA-2-gluA-28-glc	no. 20<br>no. 19<br>no. 25	[1]
170	57.385[c]	$C_{51}H_{84}O_{21}$	1031.5465	1031.5432	3.2	$[M-H]^-$	MS²[1031.5465]: 988.5526(100), 987.5593(75.36)<br>MS²[987.5459]: 945.5400(100), 927.5312(31.32), 783.4934(15.37), 765.4750(11.51)<br>MS³[987.5459→945.5405]: 783.4947(100)	PPD-glc-glc-glc-mal[#]	no. 18	[1]
171	57.430[b]	$C_{48}H_{76}O_{19}$	955.4861	955.4908	-4.92	$[M-H]^-$	MS²[955.4858]: 793.4378(100), 613.3683(34.71), 569.3837(41.66), 523.3772(66.57), 455.3529(10.11), 453.3321(12.25)<br>MS³[955.4858→793.4379]: 613.3738(100), 455.3581(87.44)	Isomer of ginsenoside Ro	no. 19<br>no. 20	[1]
172[*]	57.628[a]	$C_{42}H_{70}O_{13}$	781.4719	781.4744	-3.20	$[M-H]^-$	MS²[781.4721]: 635.4131(100), 475.3577(51.43)	Isomer of ginsenoside Rg_1	no. 20	[1]
173	57.777[c]	$C_{47}H_{80}O_{17}$	915.5381	915.5323	6.34	$[M-H]^-$	MS²[915.5378]: 753.4779(100), 621.4397(98.73), 603.4221(5.70), 459.3835(51.74), 375.2984(6.65)<br>MS³[915.5378→753.4779]: 621.4420(100), 373.2729(40.08)	PPD-glc-glc-xyl	no. 18<br>no. 20<br>no. 19<br>no. 25	[1]
174[*]	57.887[a]	$C_{41}H_{66}O_{13}$	765.4440	765.4431	1.18	$[M-H]^-$	MS²[765.4440]: 603.3800(59.04), 441.3398(100)	Unknown($C_{29}H_{46}O_3$)-glc-glc<br>(First found in ginseng)	no. 20<br>no. 19	
175	57.967[b]	$C_{47}H_{80}O_{17}$	961.4347	961.5378	-3.22	$[M+HCOO]^-$	MS²[961.5345]: 915.5190(100)<br>MS³[961.5345→915.5190]: 753.4780(100), 621.4687(50.36), 459.3804(62.71)	PPD-glc-glc-xyl	no. 19<br>no. 20<br>no. 25	[1]
176	58.115[a]	$C_{53}H_{90}O_{22}$	1077.5791	1077.5851	-5.57	$[M-H]^-$	MS²[1077.5791]: 945.5344(29.36), 915.5249(21.95), 783.4918(54.65), 621.4333(100), 459.3844(27.06)	Isomer of ginsenoside Rc	no. 20	[1]

no.	Retention Time	Molecular Formula	Measured (*m/z*)	Predicted (*m/z*)	Diff (ppm)	Ion	ESI-MSⁿ Data	Identification	Sample	Ref
							MS³[1077.5791→621.4333]: 459.3886(100), 374.2697(100)			
177	58.373[b]	$C_{57}H_{94}O_{23}$	618.3041	618.3075	-5.50	$[M+HCOO-H]^{2-}$	MS²[595.3010(2)]: 1145.6196(37.56), 1077.5837(100)<br>MS³[595.3010(2)→1145.6191]: 1077.5565(100), 1059.5664(36.56), 459.3886(36.56)	PPD-glc-xyl-glc-glc-but[#]	no. 19<br>no. 20<br>no. 18	[1]
178	58.602[a]	$C_{48}H_{82}O_{18}$	945.5639	945.5428	-6.24	$[M-H]^-$	MS²[945.5366]: 783.4861(100), 621.4294(56.04), 459.3821(20.52)	Isomer of Ginsenoside Rd	no. 18<br>no. 20<br>no. 19	[1]
179	58.708[c]	$C_{50}H_{78}O_{20}$	997.4961	997.5014	-5.31	$[M-H]^-$	MS²[997.4958]: 835.4599(6.75), 793.4380(100), 731.4357(9.55), 613.3750(30.88), 569.3865(68.78), 523.3828(93.92), 455.3531(18.19)	OA-gluA-glc-glc-AcO[#]	no. 18	[1]
180*	59.250[d]	$C_{48}H_{74}O_{20}$	1015.5035	1015.4755	27.57	$[M+HCOO]^-$	MS²[1015.5033]: 969.4981(58.46), 807.4443(100), 795.4479(7.14)<br>MS³[1015.5033→807.4439]: 455.3513(100)	OA-gluA-gluA-glc[#]<br>(First found in ginseng)	no. 18<br>no. 20<br>no. 19<br>no. 25	
181*	59.155[a]	$C_{36}H_{60}O_9$	681.4189	681.4219	-4.4	$[M+HCOO]^-$	MS²[681.4187]: 635.4126(100), 473.3630(99.79)<br>MS³[681.4187→635.4126]: 473.3592(100), 389.2573(14.61)	Ginsenoside Rh_7 or its isomer<br>(First found in ginseng)	no. 20<br>no. 19	
182	59.157[c]	$C_{47}H_{80}O_{17}$	961.5405	961.5378	2.81	$[M+HCOO]^-$	MS²[961.5407]: 915.5312(100)<br>MS³[961.5407→915.5309]: 783.4910(100), 621.4302(63.72), 459.3772(27.26)	PPD-3-glc-xyl-20-glc	no. 18<br>no. 20<br>no. 19<br>no. 25	[1]
183	59.375[a]	$C_{50}H_{84}O_{19}$	987.5471	987.5534	-6.38	$[M-H]^-$	MS²[987.5471]: 945.5318(100), 927.5255(23.90), 783.4851(28.51), 621.4324(10.64)<br>MS³[987.5471→945.5316]: 783.4866(100), 621.4312(39.74), 459.3859(16.47)<br>MS⁴[987.5471→945.5316→783.4869]: 459.3806(100)	Quinquenoside III or its isomer	no. 20<br>no. 19<br>no. 25	[1]
184	60.030[b]	$C_{36}H_{62}O_9$	683.4381	683.4376	0.73	$[M+HCOO]^-$	MS²[683.4383]: 637.4259(100), 537.3386(4.21), 475.3783(8.47)<br>MS³[683.4383→637.4257]: 537.3410(10.97), 475.3783(100), 387.3258(16.50)	Ginsenoside F_1 or its isomer	no. 18<br>no. 20<br>no. 19	
185*	60.048[a]	$C_{42}H_{70}O_{13}$	781.4727	781.4744	-2.18	$[M-H]^-$	MS²[781.4730]: 619.4193(100), 457.3731(20.87)	(A2-a)/(B7-b)/B9-glc-glc<br>(First found in ginseng)	no. 20<br>no. 19	[1][Q,N]
186	60.257[c]	$C_{51}H_{86}O_{21}$	987.5473	987.5534	-6.18	$[M-H]^-$	MS²[987.5474]: 945.5359(100), 783.4869(33.49), 927.5241(23.70), 765.4764(11.31), 621.4334(10.23), 459.3835(4.18)<br>MS³[987.5474→945.5355]: 784.4897(100), 783.4837(97.61), 621.4306(94.23), 459.3842(47.60)	Isomer of pseudo-ginsenoside Re_1	no. 18<br>no. 19	[1]
187	60.795[c]	$C_{48}H_{82}O_{17}$	929.5480	929.5479	3.28	$[M-H]^-$	MS²[929.5480]: 783.4949(91.23), 621.4327(100), 459.3805(46.57), 375.2880(7.35)<br>MS³[929.5480→621.4327]: 459.3840(100)	PPD-3-glc-20-glc-rha	no. 18<br>no. 20<br>no. 25	[1]
188	61.135[a]	$C_{42}H_{70}O_{12}$	765.4805	765.4795	1.31	$[M-H]^-$	MS²[765.9879]: 619.4194(100), 601.4092(51.03), 457.3683(20.59)	(B7-b)/(A2-a)-glc-rha	no. 20<br>no. 19<br>no. 18<br>no. 25	[1]

no.	Retention Time	Molecular Formula	Measured (*m/z*)	Predicted (*m/z*)	Diff (ppm)	Ion	ESI-MSⁿ Data	Identification	Sample	Ref
189	61.228[b]	$C_{42}H_{72}O_{13}$	829.4925	829.4955	-3.62	$[M+HCOO]^-$	MS²[829.4925]: 783.4878(69.33), 621.4319(100), 459.3801(18.76) MS³[829.4925→621.4318]: 537.3498(11.46), 459.3872(100), 375.2872(13.79)	Ginsenoside F_2 or its isomer	no. 18 no. 20 no. 19 no. 25	[1]
190	61.490[a]	$C_{42}H_{70}O_{12}$	765.4789	765.4795	-0.78	$[M-H]^-$	MS²[765.7203]: 619.4183(100), 621.4192(23.14), 601.4095(42.05), 457.3687(30.35)	(B7-b)/(A2-a)-glc-rha	no. 20 no. 19 no. 18	[1]
191	61.680[a]	$C_{52}H_{86}O_{19}$	1013.5581	1013.5691	-10.85	$[M-H]^-$	MS²[1013.5582]: 945.5358(100), 783.4784(13.01), 621.4308(1.05) MS³[1013.5582→945.5356]: 783.4861(100), 622.4212(54.53), 621.4332(36.23), 459.3828(63.59)	PPD-glc-glc-glc-but[#]	no. 20 no. 25	[1]
192[*]	61.837[a]	$C_{42}H_{70}O_{13}$	781.4763	781.4744	2.43	$[M-H]^-$	MS²[781.4765]: 619.4236(100), 457.3813(13.98)	(B7-b)/(A2-a)-glc-glc[#] (First found in ginseng)	no. 20	[1][Q,N]
193	62.745[a]	$C_{42}H_{72}O_{13}$	783.4896	783.4900	-0.51	$[M-H]^-$	MS²[783.4896]: 621.4308(89.65), 537.3368(17.85), 459.3811(100), 375.2873(46.39) MS³[783.4896→621.4309]: 537.3410(100)	Gypenoside LXXV or its isomer	no. 18 no. 20 no. 25	[1]
194	63.315[b]	$C_{42}H_{66}O_{14}$	793.4410	793.4380	3.78	$[M-H]^-$	MS²[793.4409]: 613.3699(100), 455.3511(49.33), 453.3356(84.76) MS³[793.4409→613.3701]: 455.3581(100)	OA-3-gluA-glc	no. 18 no. 19	[1]
195	63.885[a]	$C_{41}H_{70}O_{12}$	799.4837	799.4849	-1.50	$[M+HCOO]^-$	MS²[799.4836]: 753.4742(100), 621.4320(53.65), 459.3886(15.12)	Compound Y or its isomer	no. 20 no. 19	
196[*]	65.975[c,*]	$C_{36}H_{58}O_8$	663.4145	663.4114	4.67	$[M+HCOO]^-$	MS²[663.4144]: 617.4003(15.34), 455.3504(100)	OA-glc (First found in ginseng)	no. 18	
197	66.682[a]	$C_{36}H_{62}O_8$	667.4412	667.4427	-2.25	$[M+HCOO]^-$	MS²[667.4413]: 621.4311(100), 459.3821(47.18) MS³[667.4413→621.4313]: 459. 3886(100)	PPD-glc	no. 20 no. 19	[1]
198[*]	66.723[a]	$C_{36}H_{56}O_9$	632.3900	632.3924	-3.96	$[M-H]^-$	MS²[631.3826]: 455.3527(100)	OA-gluA (First found in ginseng)	no. 20	
199	67.482[a]	$C_{36}H_{62}O_8$	667.4396	667.4427	-4.64	$[M+HCOO]^-$	MS²[667.4398]: 621.4339(100), 459.3804(16.92) MS³[667.4398→621.4340]: 537.3410(100), 460.3939(100)	PPD-glc	no. 20 no. 19	[1]

glc, glucosyl; gluA, glucuronic acid moiety; rha, rhamnosyl; xyl, xylosyl; AcO, acetyl; mal, malonyl; but, butenoyl; pro, propiolyl.

[a] Retention time extracted from the chromatogram of wild ginseng (no. 20);
[b] Retention time extracted from the chromatogram of wild ginseng (no. 19);
[c] Retention time extracted from the chromatogram of ginseng under forest (no. 18);
[d] Retention time extracted from the chromatogram of cultivated ginseng (no. 25);
[N] Compound has already been detected from *Panax notoginseng*;
[O] Compound has already been detected from Shenshao Tablet;
[Q] Compound has already been detected from *Panax quinquefolium*;
[R] Compound has already been detected from Korea red ginseng;
[S] Compound has already been detected from steamed ginseng;
[*] Saponins were identified from *Panax ginseng* for the first time;
[**] Potential new compounds;
[#] Orders and/or locations of sugar residues and/or substituted groups could not be predicted.

Reference:

1. Yang, W.Z., Ye, M., Qiao, X., Liu, C.F., Miao, W.J., Bo, T., Tao, H.Y., Guo, D.A. A strategy for efficient discovery of new natural compounds by integrating orthogonal column chromatography and liquid chromatography/mass spectrometry analysis: Its application in *Panax ginseng*, *Panax quinquefolium* and *Panax notoginseng* to characterize 437 potential new ginsenosides. *Anal Chim Acta*. **2012**, 739, 56-66.
2. Tung, N.H., Song, G.Y., Minh, C.V., Kiem, P.V., Jin, L.G., Boo, H.J., Kang, H.K., Kim, Y.H. Steamed ginseng-leaf components enhance cytotoxic effects on human leukemia HL-60 cells. *Chem Pharm Bull*. **2010**, 58, 1111-1115.
3. Park, J.D., Lee, Y.H., Kim, S.I. Ginsenoside Rf_2, a new dammarane glycoside from korean red ginseng (*Panax ginseng*). *Arch Pharm Res.* **1998**, 21, 615-617.
4. Miao, W.J., Hu, Y., Jia, L., Zhang, C.X., Yang, W.Z., Zhang, P., Guo, D.A. Profiling and identification of chemical components of Shenshao Tablet and its absorbed components in rats by comprehensive HPLC/DAD/ESI-MS^n analysis. *Chinese Journal of Natural Medicines*. **2018**, 16, 791-800.

Table S2. Saponins detected from "all the four ginseng samples" and "both the two wild ginseng samples" by HPLC-IT-TOF-MS^n

**21 saponins detected from all the four ginseng samples** (including 4 rhamnosides and 8 xylosides)	**50 saponins detected from both the two wild ginseng samples** (including 17 rhamnosides and 11 xylosides)	
1. (B1-b)/(B2-a)	1. (C3-b)-6-glc-20-glc-rha[△]	26. Ginsenoside Rg_{31} or its isomer[△]
2. Ginsenoside Re_5 or its isomer	2. (B2-b)-20-glc[△]	27. (B8-b)-rha-xyl[*, #]
3. Ginsenoside Re_4 or its isomer	3. (C1-b)/(C3-b)/(B10)-6-glc-$C_6H_8O_5$-20-rha[#]	28. PPT-6-glc-xyl-20-glc-glc
4. Notoginsenoside R_1	4. (B2-b)-glc-xyl[△]	29. (B4-a)-6-glc-xyl-20-glc-glc
5. Ginsenoside Rg_1	5. Quinquenoside L_9 or its isomer	30. (B8-b)-glc[△]
6. Ginsenoside Re	6. Ginsenoside SL_1 or its isomer	31. (C2)/(C3-a)-glc
7. PPT-glc-xyl	7. Vinaginsenoside R_{12} or its isomer[△]	32. Unknown($C_{27}H_{48}O_4$)-glc-glc[#]
8. Notoginsenoside R_4 or its isomer	8. B6/(B3-b)/(B4-b)-glc-glc-glc	33. Isomer of notoginsenoside R_1
9. Ginsenoside Rf	9. (B2-b)-glc-xyl (isomer of no. 4) [△]	34. PPT-rha-glc-rha
10. Ginsenoside Rb_1	10. (B2-b)-glc (isomer of no. 2) [△]	35. A3-glc-glc
11. Ginsenoside F_3, Notoginsenoside R_2, or their isomer	11. Unknown($C_{29}H_{48}O_4$)-glc-glc-glc[△]	36. Unknown($C_{26}H_{44}O_5$)-glc-rha[#]
12. Ginsenoside Rc or its isomer	12. B6/(B3-b)-glc[△]	37. (B4-b)-3-glc-20-rha
13. Ginsenoside Rh_1	13. Ginsenoside Re_8 or its isomer	38. (B3-b)/B6-glc[△]
14. Ginsenoside Rg_2	14. (B3-b)/(B4-b)-6-glc-glc	39. Ginsenoside Ra_1/Ra_2 or its isomer
15. Ginsenoside Rb_2	15. (B4-b)/B4-glc	40. Ginsenoside F_1
16. Ginsenoside Rd	16. (B3-b)-glc-rha	41. Isomer of ginsenoside Rg_1
17. PPD-glc-glc-xyl	17. 25-hydroxy ginsenoside Rg_2 or its isomer	42. Unknown($C_{29}H_{46}O_3$)-glc[△]
18. OA-gluA-gluA-glc	18. B6-glc-rha[△]	43. Isomer of ginsenoside Rg_1
19. PPD-3-glc-xyl-20-glc (isomer of no. 17)	19. Unknown($C_{31}H_{52}O_7$)-glc-rha[#]	44. Isomer of ginsenoside Ro
20. (B7-b)/(A2-a)-glc-rha	20. (B4-b)/(B6)-glc[△]	45. Unknown($C_{29}H_{46}O_3$)-glc-glc[△]
21. Ginsenoside F_2 or its isomer	21. Floralginsenoside N or its isomer[*]	46. Ginsenoside Rh_7 or its isomer[△]
	22. A3-glc-glc-rha	47. (A2-a)/(B7-b)/B9-glc-glc[△]
	23. (B3-b)/(B4-b)/B6/C2-glc-xyl	48. Compound Y or its isomer
	24. Isomer of ginsenoside Re	49. PPD-glc
	25. (B4-b)/(B6)-glc (isomer of no. 20) [△]	50. PPD-glc (isomer of no. 48)

[*], saponins were both rhamnosides and xylosides; [#], potential new compounds; [△], first found in ginseng.

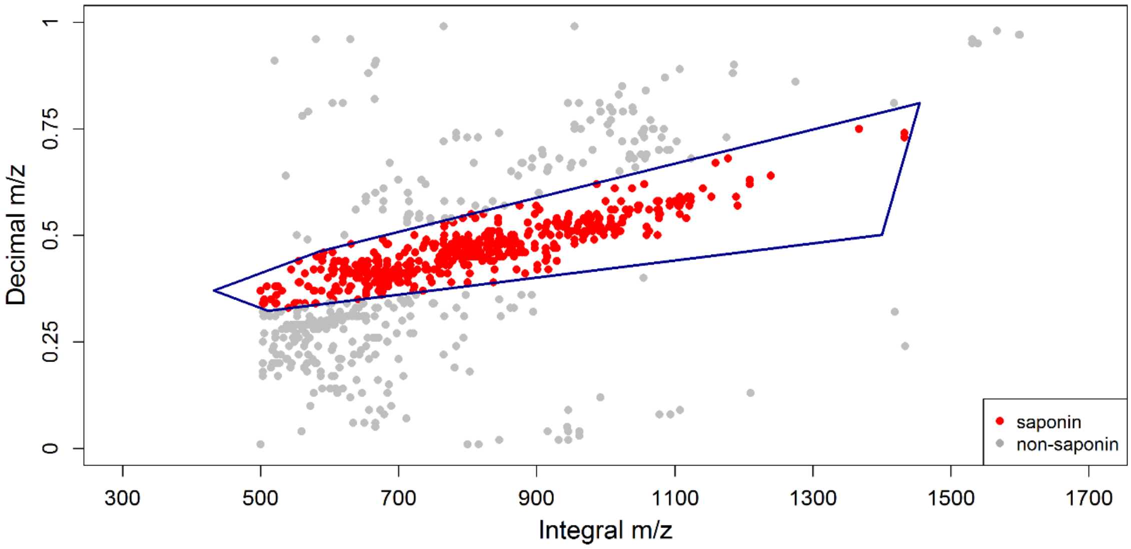

Figure S20. Potential saponin precursors (red spots) extracted by "5-point screening" method with the 5 points (x, y) of estimated lowest *m/z* (441, 0.3783), estimated highest mass defection (503, 0.3378), estimated lowest *m/z* with the lowest mass defection (591, 0.4630), estimated highest *m/z* with the highest mass defection (1421, 0.5505), and estimated highest *m/z* with the lowest mass defection (1467, 0.8150), in which x represented the integral part of *m/z*, and y represents the decimal part of *m/z*.

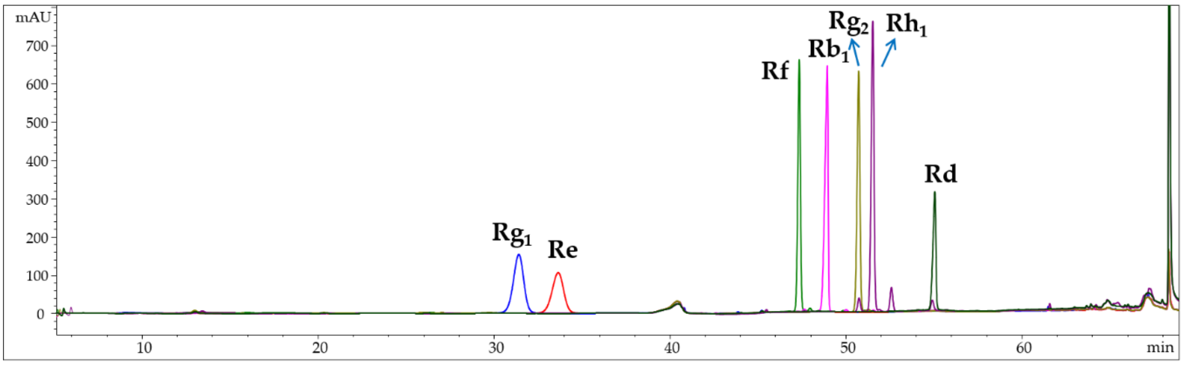

Figure S21. Overlapped chromatograms of reference substances of ginsenosides Rg_1, Re, Rf, Rb_1, Rg_2, Rh_1, and Rd.

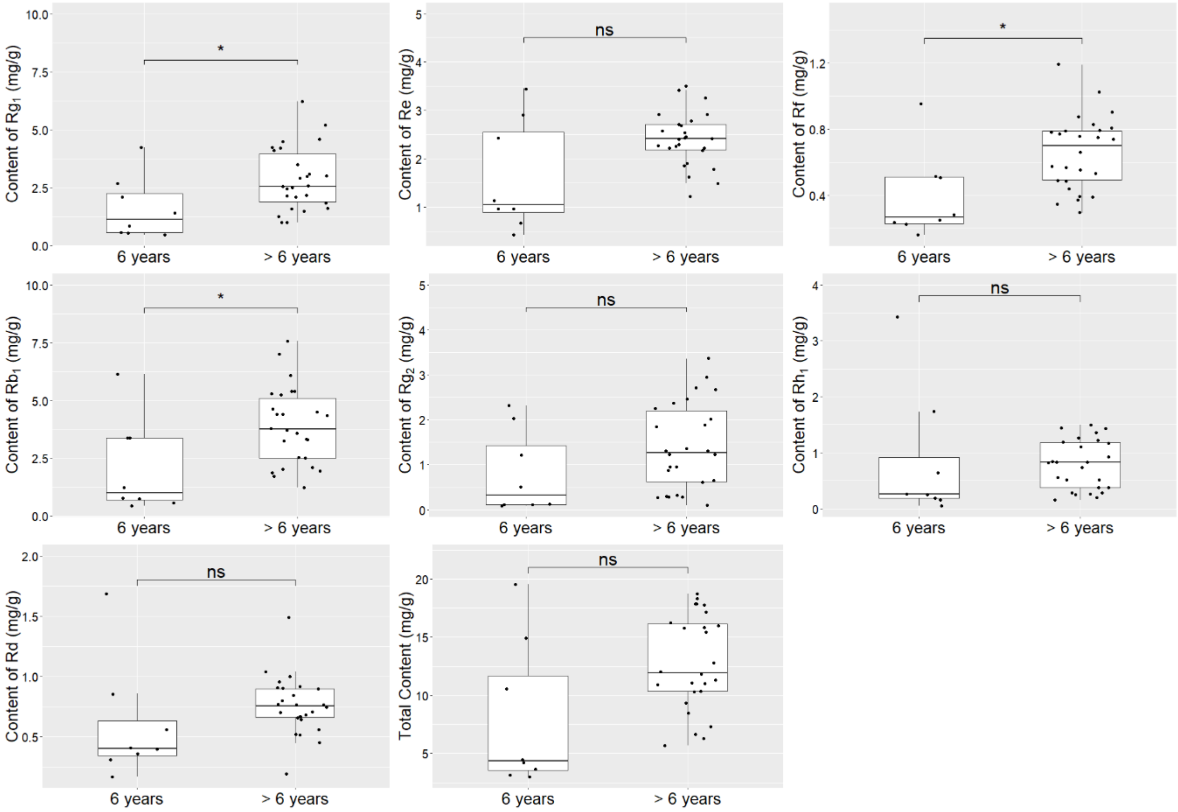

Figure S22. Comparison of the contents of seven ginsenosides and their total content between ginseng under forest with ages older than 6 years (n = 26), and ginseng under forest, cultivated ginseng with age of 6 years (n = 8; * p<0.05; ns, no significance).

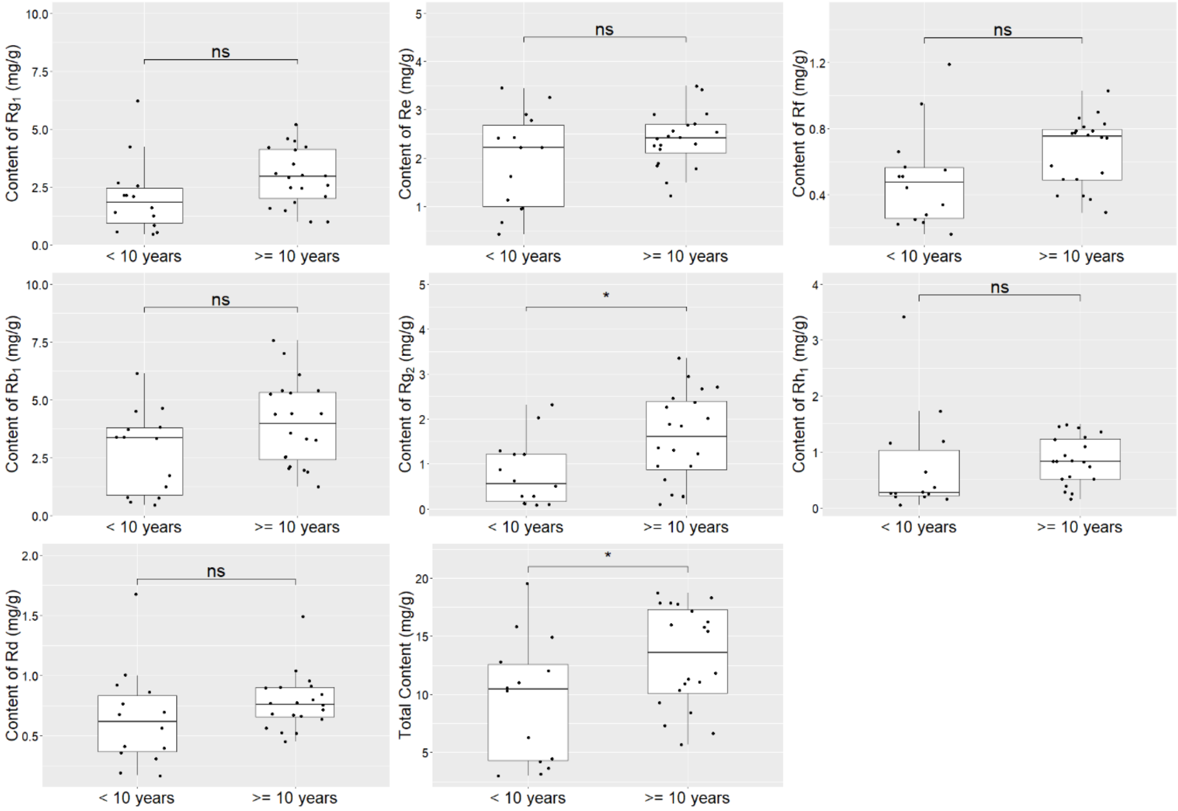

Figure S23. Comparison of the contents of seven ginsenosides and their total content between ginseng under forest with ages older than 10 years (n = 20), and ginseng under forest, cultivated ginseng with age younger than 10 years (n = 14; ns, no significance).

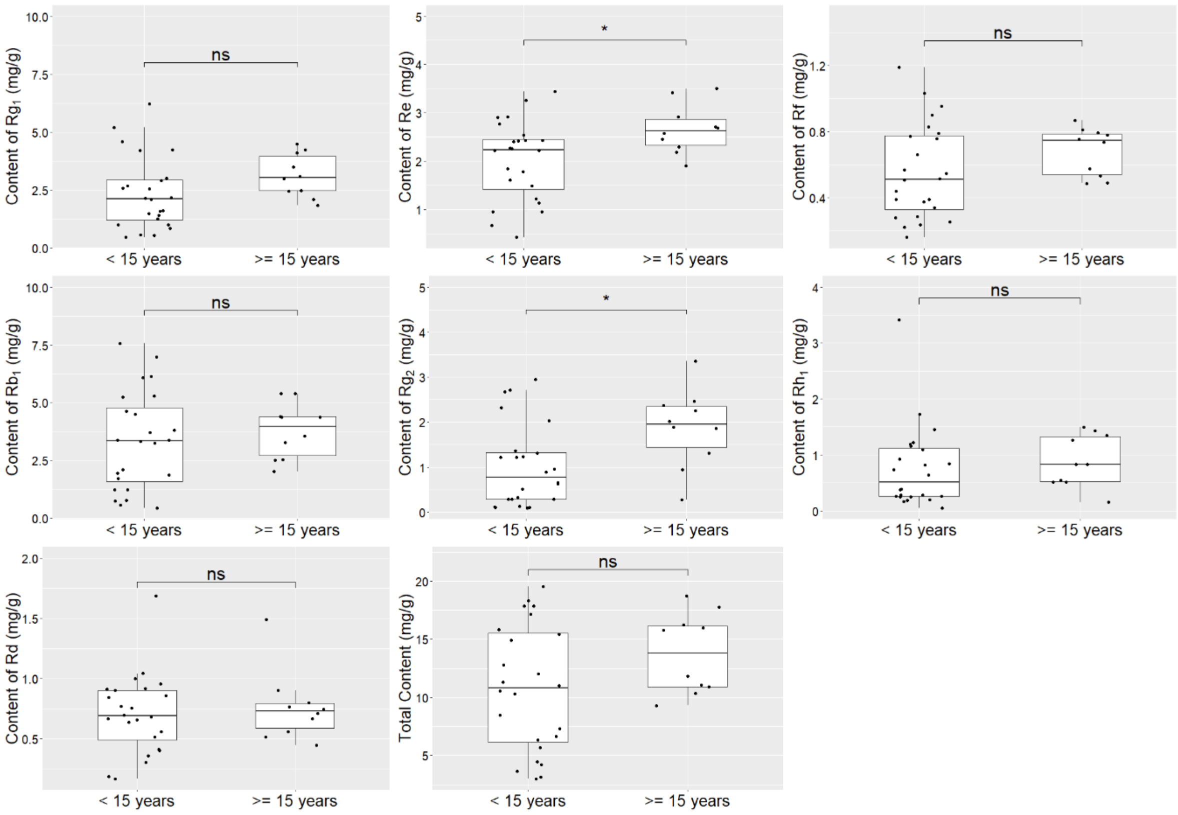

Figure S24. Comparison of the contents of seven ginsenosides and their total content between ginseng under forest with ages older than 15 years (n = 10), and ginseng under forest, cultivated ginseng with age younger than 15 years (n = 24; ** *p*<0.01; ns, no significance).

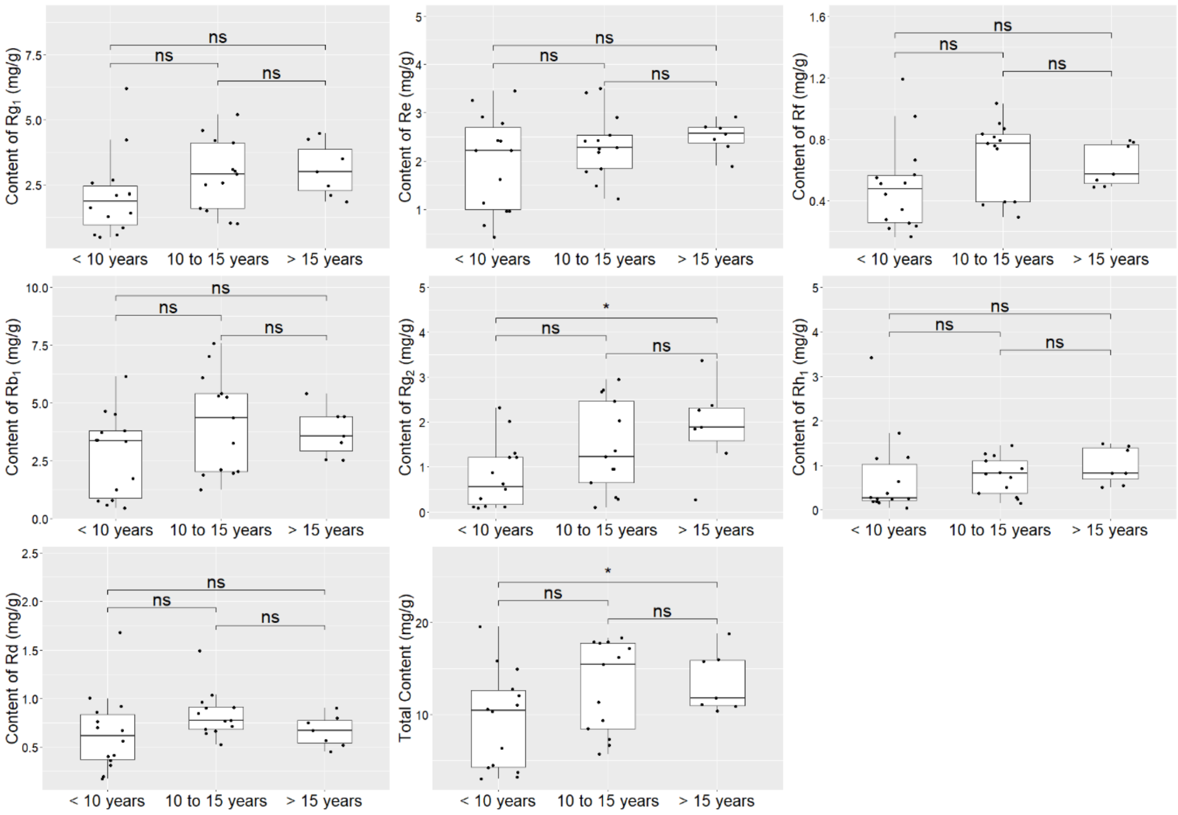

Figure S25. Comparison of the contents of seven ginsenosides and their total content between ginseng under forest with ages older than 15 years (n = 7), between 10 to 15 years (n = 13) and ginseng under forest, cultivated ginseng with age younger than 10 years (n = 14; * *p*<0.01; ns, no significance).

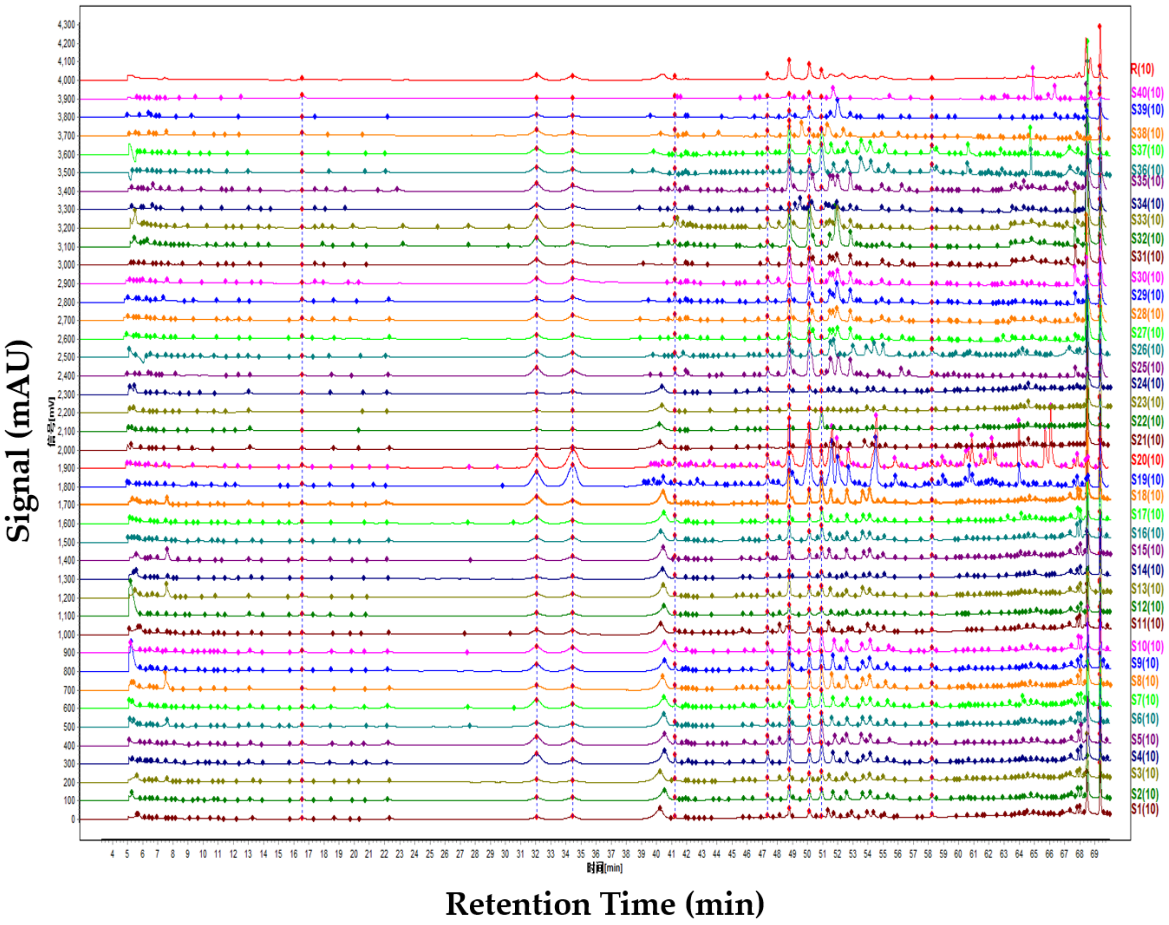

Figure S26. Chromatographic fingerprints obtained by HPLC-DAD of 40 ginseng samples with retention time correction by 10 common peaks using Similarity Evaluation System for Chromatographic Fingerprint of TCM version 2012 (S1–S40: ginseng samples nos. 1–40; R: reference fingerprint automatically generated by software)

Table S3. Peak areas (manually integrated) of the five peaks within the characteristic peak pattern and the ratio of signal-to-noise

Sample no.	Count of Peaks[a]	Noise (mAU)	Peak Area/mAU*s (S/N[b] Ratio)				
			Peak 1	Peak 2	Peak 3	Peak 4	Peak 5
**Ginseng under forest**							
1	4	0.09278	7.36 (3.5)	23.59 (14.0)	108.90 (60.7)	39.55 (13.3)	64.43 (25.0)
2	4	0.06339		29.81 (24.3)	132.32 (95.2)	35.03 (20.9)	67.13 (37.5)
3	4	0.09313	5.50 (3.4)	23.78 (13.7)	137.37 (67.9)	52.44 (17.3)	86.40 (33.0)
4	4	0.06900	6.09 (4.1)	43.45 (33.7)	235.36 (152.4)	45.96 (26.6)	172.58 (92.1)
5	4	0.1017	7.37 (4.1)	54.45 (26.5)	210.75 (100.8)	52.16 (16.9)	188.34 (65.8)
6	5	0.05127	15.32 (14.2)	24.11 (22.3)	141.60 (114.9)	22.19 (18.1)	85.91 (61.2)
7	4	0.08162	19.25 (12.3)	16.65 (9.0)	93.57 (51.2)	46.42 (22.9)	161.36 (73.8)
8	3	0.07752		17.48 (8.4)	180.73 (71.9)	24.53 (12.9)	115.27 (46.5)
9	4	0.1396	5.33 (2.2)	50.98 (17.7)	202.54 (66.9)	47.54 (12.7)	153.71 (39.7)
10	5	0.04883	12.15 (11.7)	31.65 (31.8)	197.74 (188.4)	35.79 (25.9)	140.42 (102.4)
11	4	0.08879	10.82 (5.9)	21.14 (12.8)	164.79 (89.8)	44.73 (14.9)	95.10 (37.8)
12	4	0.06145		39.39 (35.7)	180.33 (143.3)	49.40 (31.1)	44.30 (30.7)
13	4	0.05048		93.08 (63.4)	261.62 (175.3)	44.65 (35.2)	42.55 (24.8)
14	4	0.04109		26.97 (33.2)	106.17 (120.0)	26.24 (22.6)	48.48 (37.5)
15	5	0.06113	14.57 (11.8)	35.17 (21.9)	151.84 (93.4)	22.86 (15.5)	82.05 (43.4)
16	4	0.1012	8.21 (4.1)	37.01 (19.8)	174.42 (84.7)	61.70 (20.2)	123.99 (44.0)
17	4	0.1066	36.24 (18.8)	17.06 (8.2)	138.56 (59.3)	31.68 (11.3)	88.86 (32.9)
18	4	0.1026	10.10 (3.8)	41.26 (18.2)	187.00 (79.3)	36.41 (14.3)	180.16 (59.6)
27	4	0.1473	14.55 (5.2)	37.56 (10.2)	223.55 (73.9)	61.65 (15.8)	141.25 (35.2)
28	4	0.1546	16.65 (5.8)	38.43 (10.2)	209.74 (67.5)	68.88 (16.7)	170.69 (39.6)
29	3	0.1725	19.40 (4.8)	33.79 (7.2)	226.33 (60.3)	109.29 (17.6)	165.17 (32.4)
30	4	0.1753		52.76 (13.6)	286.23 (70.6)	101.30 (20.2)	155.20 (30.5)
31	4	0.1066	7.34 (2.7)	26.67 (12.5)	126.69 (54.6)	46.16 (14.8)	90.69 (28.9)
32	4	0.1039	45.19 (22.2)	12.33 (4.8)	211.31 (96.0)	92.47 (28.8)	191.21 (64.5)
33	5	0.1373	106.61 (37.7)	49.34 (14.1)	129.04 (44.8)	62.81 (14.7)	224.11 (58.7)
34	3	0.1536		31.81 (9.8)	130.13 (39.3)	102.35 (23.4)	126.36 (29.7)
35	3	0.1337	6.39 (2.8)	26.49 (9.2)	209.29 (71.7)	203.76 (52.8)	190.61 (50.6)
**Wild ginseng**							
19	5	0.1336	22.31 (11.3)	39.60 (14.3)	249.43 (84.0)	75.79 (21.4)	132.78 (37.0)
20	5	0.1198	34.56 (17.3)	54.32 (20.7)	298.45 (104.3)	84.70 (26.0)	80.89 (27.2)
**Cultivated ginseng**							
21	2	0.05706			44.70 (32.8)	11.28 (7.4)	37.84 (22.4)
22	0	0.1065		8.19 (3.5)	20.92 (9.2)		
23	1	0.03883			42.76 (49.1)		
24	2	0.07222		7.21 (4.7)	53.89 (33.0)	12.63 (6.8)	22.03 (11.0)
25	1	0.1691		14.72 (4.3)	128.79 (32.7)	13.95 (4.2)	11.03 (3.4)
26	3	0.08311		15.10 (9.8)	119.14 (66.7)	27.26 (14.4)	55.86 (26.6)
36	3	0.04377	5.09 (6.2)	11.14 (11.3)	58.66 (64.8)	5.89 (6.6)	61.46 (49.3)
37	2	0.2194		13.25 (2.7)	65.06 (14.1)	10.32 (2.1)	62.50 (10.3)
38	1	0.07459			330.71 (219.9)	6.50 (5.4)	
39	3	0.06371		6.85 (6.6)	298.93 (229.0)	17.26 (13.1)	67.44 (40.9)
40	2	0.1715		7.24 (1.7)	57.72 (14.3)	56.17 (10.3)	35.49 (7.5)

[a] Number of peaks with S/N over 10.
[b] S/N: signal-to-noise.
